# Tetrahydrobenzimidazole TMQ0153 targets OPA1 and restores drug sensitivity in AML via ROS-induced mitochondrial metabolic reprogramming

**DOI:** 10.1186/s13046-025-03372-0

**Published:** 2025-04-07

**Authors:** Su Jung Park, Claudia Cerella, Jin Mo Kang, Jinyoung Byun, David Kum, Barbora Orlikova-Boyer, Anne Lorant, Michael Schnekenburger, Ali Al-Mourabit, Christo Christov, Juyong Lee, Byung Woo Han, Marc Diederich

**Affiliations:** 1 Research Institute of Pharmaceutical Sciences & Natural Products Research Institute, College of Pharmacy, Seoul National University, Seoul, 08826 Republic of Korea; 2 Laboratoire de Biologie Moléculaire du Cancer, BAM3 Pavillon 2, 6A Rue Nicolas-Ernest Barblé, L-1210 Luxembourg, Luxembourg; 3 College of Pharmacy, Seoul National University, Seoul, 08826 Republic of Korea; 4 CNRS, Institut de Chimie des Substances Naturelles, Université Paris-Saclay, Gif-Sur-Yvette, 91190 France; 5 Service d’Histologie, Faculté de Médicine, Université de Lorraine, and INSERM U1256 NGERE, 54000 Nancy, France; 6 Department of Molecular Medicine and Biopharmaceutical Sciences, Graduate School of Convergence Science and Technology, College of Medicine, Seoul National University, 1 Gwanak-Ro, Gwanak-Gu, Seoul, 08826 Korea; 7 Present address: Department of Cancer Research, Luxembourg Institute of Health (LIH), BAM Pavillon 2, 6A Rue Nicolas-Ernest Barblé, L-1210 Luxembourg, Luxembourg; 8 Present address: Luxembourg Centre for Systems Biomedicine, Bioinformatics Core, Roudeneck, 1, Boulevard du Jazz, Esch-sur-Alzette, L-4370 Luxembourg

**Keywords:** Metabolic reprogramming, OXPHOS, Glycolysis, Drug resistance, Monocytic myeloid leukemia, Glutathione

## Abstract

**Background:**

Acute myeloid leukemia (AML) is a highly aggressive cancer with a 5-year survival rate of less than 35%. It is characterized by significant drug resistance and abnormal energy metabolism. Mitochondrial dynamics and metabolism are crucial for AML cell survival. Mitochondrial fusion protein optic atrophy (OPA)1 is upregulated in AML patients with adverse mutations and correlates with poor prognosis.

**Method:**

This study investigated targeting OPA1 with TMQ0153, a tetrahydrobenzimidazole derivative, to disrupt mitochondrial metabolism and dynamics as a novel therapeutic approach to overcome treatment resistance. Effects of TMQ0153 treatment on OPA1 and mitofusin (MFN)2 protein levels, mitochondrial morphology, and function in AML cells. In this study, we examined reactive oxygen species (ROS) production, oxidative phosphorylation (OXPHOS) inhibition, mitochondrial membrane potential (MMP) depolarization, and apoptosis. Additionally, metabolic profiling was conducted to analyze changes in metabolic pathways.

**Results:**

TMQ0153 treatment significantly reduced OPA1 and mitofusin (MFN)2 protein levels and disrupted the mitochondrial morphology and function in AML cells. This increases ROS production and inhibits OXPHOS, MMP depolarization, and caspase-dependent apoptosis. Metabolic reprogramming was observed, shifting from mitochondrial respiration to glycolysis and impaired respiratory chain activity. Profiling revealed reduced overall metabolism along with changes in the glutathione (GSH)/oxidized glutathione (GSSG) and NAD⁺/NADH redox ratios. TMQ0153 treatment reduces tumor volume and weight in MV4-11 xenografts in vivo. Combination therapies with TMQ0153 and other AML drugs significantly reduced the leukemic burden and prolonged survival in NOD scid gamma (NSG) mice xenografted with U937-luc and MOLM-14-luc cells.

**Conclusion:**

TMQ0153 targets mitochondrial dynamics by inhibiting OPA1, inducing metabolic reprogramming, and triggering apoptosis in AML cells. It enhances the efficacy of existing AML therapies and provides a promising combination treatment approach that exploits mitochondrial vulnerability and metabolic reprogramming to improve treatment outcomes in AML.

**Graphical Abstract:**

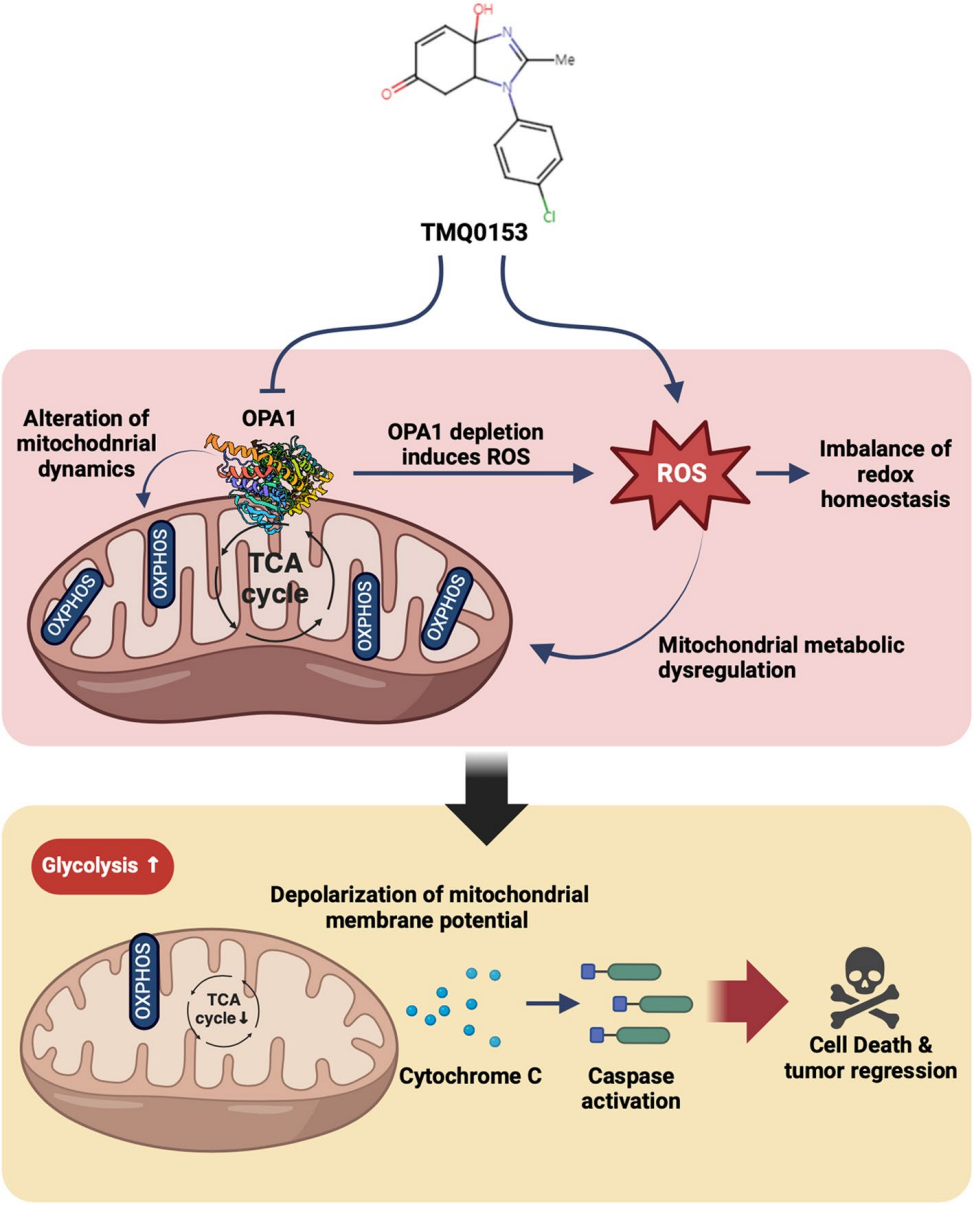

**Supplementary Information:**

The online version contains supplementary material available at 10.1186/s13046-025-03372-0.

## Background

Acute myeloid leukemia (AML) is a heterogeneous hematologic malignancy that originates from hematopoietic stem progenitor cells, and is caused by genomic alterations that are most commonly diagnosed in older individuals [1]. AML is the most prevalent form of acute leukemia in adults, with a 5-year overall survival rate of 30.5% [2]. 10% to 40% of patients with newly diagnosed acute myeloid leukemia (AML) do not achieve complete remission (CR) with intensive chemotherapy, and some may develop refractory AML [3].

Minimal residual disease (MRD) can persist after chemotherapy, thereby increasing the risk of relapse. Surviving leukemic blasts often rely on mitochondrial metabolism and oxidative phosphorylation (OXPHOS), highlighting the metabolic vulnerabilities in acute myeloid leukemia (AML) cells [4–6]. Many cancers are metabolic diseases characterized by disruption in energy production through respiration and glycolysis [7]. These metabolic abnormalities in tumor cells are frequently linked to structural and functional defects in the mitochondria, which play pivotal roles in cancer progression [8]. The involvement of mitochondria in metabolic reprogramming has spurred a growing interest in mitochondria-targeted therapies. Mitochondria-targeting drugs, often referred to as "mitocans" [9], include venetoclax, cytarabine (AraC), and 2-deoxyglucose (2-DG). Targeting mitochondrial metabolism presents a significant potential for the development of innovative therapeutic strategies for AML.

Mitochondria are highly dynamic organelles in eukaryotic cells that continuously reshape their structure through fusion and fission [10], and are essential for cristae remodeling and cell survival [11]. The ultrastructure of cristae enhances the energy conversion efficiency by scaffolding components of the respiratory chain, thereby improving the efficacy of oxidative phosphorylation (OXPHOS) [12, 13]. Mitochondrial dynamics regulate respiration, cytochrome c release during apoptosis, and reactive oxygen species (ROS) production in cancer cells [14–16]. Fusion is controlled by the GTPases optic atrophy 1 (OPA1), mitofusin (MFN)1, and 2 [17, 18], whereas fission is regulated by mitochondrial fission protein 1 (FIS1) and dynamin-related protein 1 (DRP1) [19, 20]. Drug-resistant cells rely on mitochondrial adenosine triphosphate (ATP) generation and exhibit elongation. In resistant cells, OPA1 levels are increased, and pharmacological inhibitors can re-sensitize resistant cells to drug treatment, ultimately inducing apoptosis [21]. OPA1 has emerged as a novel cancer biomarker associated with patient prognosis and plays a critical role in the regulation of apoptosis, proliferation, angiogenesis, and metastasis [22].

More than 90% of ATP is produced under aerobic conditions via the TCA cycle and OXPHOS [23]. NAD^+^ and NADH are essential cofactors in redox reactions throughout mitochondrial metabolism that maintain redox homeostasis [24, 25]. Thus, the NAD^+^/NADH ratio is a key marker of metabolic alterations and physiological status [26]. Cancer cells undergo metabolic reprogramming to meet their energy demands and support proliferation, often relying on the Warburg Effect [27]. Emerging evidence has linked mitochondrial metabolic alterations to AML tumorigenesis [28].

AML is characterized by elevated ROS levels, which are hallmarks of the disease [29]. To counteract ROS, AML cells rely on robust antioxidant defenses, including increased glutathione (GSH) production [30] and overexpression of superoxide dismutase (SOD) [31] and peroxiredoxins (PRDXs) [32], which protect against oxidative stress-induced cell death [33]. Chemotherapy-generated ROS can overwhelm these defenses and lead to AML cell death [34]. While most GSH resides in the cytosol, 10–15% is present in mitochondria [35]. During ROS scavenging, GSH is converted into oxidized glutathione (GSSG) by the glutathione peroxidase (GPx) family, which reduces hydrogen peroxide (H₂O₂) to water [36]. GSSG can be recycled back into GSH by GSSG reductase (GR), making the GSH/GSSG ratio a vital indicator of redox homeostasis in AML cells [37].

The tetrahydrobenzimidazole compound TMQ0153 was synthesized from para-benzoquinone [38]. TMQ0153 shares structural similarities with the herbicide diuron, which disrupts photosystem II in plant cells and causes cellular damage [39, 40]. We have previously investigated the anticancer properties of various tetrahydrobenzimidazole derivatives in hematopoietic cancer cell lines. Among these, TMQ0153 exhibited potent cytotoxicity against myeloid leukemia cells, inducing dose-dependent immunogenic necrosis in chronic myeloid leukemia models [41].

This study aimed to identify the molecular target of TMQ0153 and explore its effects on mitochondrial dynamics and metabolism in order to re-sensitize cancer cells to treatment. We will examine how these mechanisms promote AML cell death in vitro and in vivo and evaluate their efficacy as standalone or alongside the FLT3 inhibitors gilteritinib or venetoclax-azacitidine.

## Methods

### Compounds

Tetrahydrobenzimidazole (TMQ) 0153 was synthesized from *p*-benzoquinone as previously described [41]. The physicochemical properties of TMQ0153 are listed in Table S1. NAC (A7250), etoposide (VP-16–213, E1383), and midostaurin (M1213) were purchased from Sigma-Aldrich (St. Louis, MO, USA). Caspase inhibitor 1 (zVAD-FMK, 627610) was obtained from Calbiochem (San Diego, CA). Tiron (sc-253699), Trolox (sc-200810), and buthionine sulfoximine (BSO, sc-200824) were purchased from Santa Cruz Biotechnology (Dallas, TX, USA). Gilteritinib (ASP2215) was purchased from Sellekchem (Houston, TX). Venetoclax (HY-15531) and azacitidine (HY-10586) were purchased from MedChemExpress (Monmouth Junction, NJ).

### Cell lines

AML cell lines MV4-11 (mutated FLT3-ITD) and U937 (wild-type FLT3-ITD, t(10;11)(p13;q14)), U937-Luciferase, THP-1, HL60, MOLM-14-Luciferase, and chronic myeloid leukemia (CML) cell lines K562 and multiresistant K562 (K562R) were used. MV4-11, U937, THP-1, HL60, and K562 cells were purchased from Deutsche Sammlung von Mikroorganismen und Zellkulturen (DSMZ, Braunschweig, Germany). The MOLM-14-Luciferase and U937-Luciferase cell lines were kindly provided by Dr. Jean-Emmanuel Sarry (Université Toulouse III-Paul Sabatier, France). Table S2 summarizes the cell lines used in this study.

MV4-11 and MOLM-14-Luciferase cells were cultured in RPMI 1640 medium (Lonza, Walkersville, MD, USA) supplemented with 10% (v/v) fetal bovine serum (FBS; Opti-Gold, GenDEPOT, Katy, TX, USA) and 1% penicillin–streptomycin (Lonza, Basel, Belgium) at 37 °C with 5% CO₂. U937, U937-Luciferase, HL-60, and THP-1 cells were grown in RPMI 1640 medium (Lonza) with 10% (v/v) FBS (Biowest, Riverside, MO, USA) and 1% penicillin–streptomycin. K562 cells were maintained in RPMI 1640 with 10% (v/v) FBS and 1% (v/v) antibiotic–antimycotic solution (Lonza) at 37 °C and 5% CO₂. The multi-resistant K562 (K562R) cell line was provided by Professor Dong-Wook Kim (Catholic University of Seoul, Republic of Korea) with specific cell culture conditions. After thawing, K562R cells were resuspended in 10 mL of RPMI 1640 supplemented with 25 mM HEPES, 10% fetal bovine serum (FBS), and 1% antibiotic-antimycotics. After 48 h, cell viability was assessed, and all living cells were resuspended in 20 mL of fresh, complete cell culture medium in a 75 cm^2^ flask. After 48 h, cells were counted and seeded at 500,000 cells/mL in 30 mL of fresh, complete growth medium supplemented with 1 µM imatinib. After 48 h, the cells were centrifuged, counted, and seeded for a second time at 500,000 cells/mL in 30 of mL fresh, complete growth medium supplemented with 1 µM imatinib. After 48 h, viability was assessed, and cells were washed three times in 1X PBS and resuspended in 30 mL of fresh, complete growth medium at a density of 300,000 cells/mL, without imatinib, for experimental use. All cell lines were cultured according to standard protocols and mycoplasma testing was performed monthly using the MycoAlert™ PLUS Mycoplasma Detection Kit (Lonza, Walkersville, MD, USA).

### Confocal microscopy

Cells (7 × 10^4^) were embedded in glass slides using a cytocentrifuge (Wescor, Utah, USA) before immunofluorescence staining. Cells were fixed with 10% formalin for 10 min, followed by permeabilization with 0.25% Triton X-100 for 15 min. The primary antibody against γH2AX (9718S, Cell Signaling Technology, Danvers, MA, USA) was diluted 1:200 and incubated overnight at 4 °C. After washing, the cells were incubated with a secondary anti-rabbit Alexa488 antibody (A11034, Invitrogen, Grand Island, NY, USA) for 1 h at room temperature. Fluorescence images were captured using a TCS8 Confocal Microscope (LEICA, Wetzlar, Germany). The antibodies used in this study are listed in Table S3.

For co-staining of cytochrome c and MitoTracker Green, cells were incubated with 200 nM MitoTracker Green FM (Thermo Fisher Scientific, Waltham, MA, USA) for 30 min, following the manufacturer’s guidelines. Subsequently, cytochrome c staining was performed using the same method as that described for quantifying cytochrome c release using FACS analysis (Supplementary Data). Quantification was performed using the ImageJ 1.54d software ( National Institutes of Health, Bethesda, MD, USA).

### Mitoplate and Mitocheck analysis

Mitoplate S-1 assay (#14105; BIOLOG, Hayward, CA, USA) was performed to assess mitochondrial metabolism. Cells (4 × 10^4^) were harvested and resuspended in the Biolog MAS solution (72303, BIOLOG). MV4-11 cells were permeabilized with 70 μg/ml saponin and 30 μg/ml U937. Color formation was monitored kinetically using a SpectraMax i3x microplate reader (Molecular Devices, San Jose, CA, USA) at OD590. The MitoCheck Complex Activity Assay Kits (Complex I: 700,930, Complex II: 700940, Complex II/III: 700950, Complex IV: 700990, and Complex V: 701000; Cayman, MI, USA) were used according to the manufacturer’s instructions.

### In vivo bioluminescence imaging (BLI)

NOD-SCID gamma (NSG) mice were purchased from JA Bio Inc. (Seoul, Korea). All animal experiments were approved by the Seoul National University Institutional Animal Care and Use Committee (SNU-220704–6-3). Each mouse was intravenously injected with 1 × 10⁶ MOLM-14-luc cells in 200 μL PBS. On day 4, mice were randomly divided into four groups: control (*n* = 5), TMQ0153 (35 mg/kg) (*n* = 5), gilteritinib (3 mg/kg) (*n* = 5), and TMQ0153 + gilteritinib (*n* = 5). TMQ0153 was administered intraperitoneally (i.p.) every three days, and gilteritinib was administered via oral gavage. U937-Luc cells (1 × 10^5^) were injected intravenously. On day 4, mice were randomly divided into four groups: control (*n* = 5), TMQ0153 (35 mg/kg) (*n* = 5), venetoclax (70 mg/kg) combined with azacitidine (1.5 mg/kg) (*n* = 5), and TMQ0153 combined with venetoclax and azacitidine (*n* = 5). TMQ0153 was administered intraperitoneally (i.p.) every three days, venetoclax was administered orally daily, and azacitidine was injected subcutaneously daily. Mice received an i.p. injection of 150 mg/kg D-luciferin (PerkinElmer, Waltham, MA, USA), and bioluminescence imaging (BLI) was performed using an IVIS Spectrum In Vivo Imaging System (PerkinElmer). Image analysis was conducted using Caliper Life Sciences software (PerkinElmer).

### Molecular docking simulation of TMQ compounds with OPA1

The chemical structures of the TMQ compounds were generated using an online tool (https://www.molinspiration.com/cgi/properties) and optimized using the Avogadro software. Molecular docking simulations were performed using AutoDock 4.2.6 and AutoDock Vina 1.1.5. The GDP-bound structure of OPA1 (PDB ID: 6JTG) was used as the reference. The Autogrid4 tool in AutoDock 4.2.6 determined the precise location of the small molecule binding site on GDP-bound OPA1. The Gasteiger partial charge method was employed to calculate the partial charges for TMQ compounds, GDP, and OPA1. Docking simulations were conducted with the GDP binding center as a control, followed by simulations with TMQ compounds. The binding energies were calculated using AutoDock Vina 1.1.5.

### Bioinformatics analysis

Gene expression data from healthy and tumor samples were compared using the TNMplot database (https://tnmplot.com/analysis/) [42]. Gene expression correlations were analyzed using GEPIA (http://gepia.cancer-pku.cn/) [43]. Log2 normalized RPKM values of 73 healthy bone marrow BM (*N* = 73), 542 AML, and 206 myelodysplastic syndrome (MDS) patients included in the MILE Study (GSE13159) [44] were retrieved from BloodSpot (https://www.fobinf.com) [45]. The results for pediatric AML patients are, in whole or part, based on the data generated by the Therapeutically Applicable Research to Generate Effective Treatments (https://ocg.cancer.gov/programs/target) initiative, phs000465 (TARGET AML). Primary non-redundant samples were selected for analysis (*N* = 145), except for the paired analyses. These data are available from https://portal.gdc.cancer.gov/projects. Processed expression data (log2(FPKM-Uq + 1)) were retrieved from the UCSC Xena database, Xenabrowser (www.xenabrowser.net). The results of TCGA LAML cohorts are in whole or part based on data generated by TCGA Research Network (https://www.cancer.gov/tcga). Clinical features and RPKM expression data for the TCGA LAML cohort (TCGA Legacy, *N* = 179) were retrieved from the cBioPortal (https://www.cbioportal.org). The clinical and expression processed data for the Beat AML 2.0 cohort [46], corresponding to 19 bone marrow mononuclear cell (BM MNC) specimens from healthy individuals and 596 non-redundant patient specimens taken at the diagnosis or the earliest follow-up, were made available from the Vizome database (www.vizome.org). For the paired analyses, multiple patient samples from different stages of disease progression were selected from the same cohort. The RNA-seq raw data of the Leucegene cohort were generated by the Leucegene group primarily located at IRIC in Montreal (Canada) and supported by Genome Québec, using human AML specimens provided by BCLQ, Montreal (Canada). The RPKM-processed data available from the authors were retrieved from the Gene Expression Omnibus (GEO) database (GSE49642, GSE52656, GSE62190, GSE66917, and GSE67039). After matching the GSM ID number with the patient ID, the gene expression values were integrated with the disclosed clinical features retrieved from several publications of the Leucegene group [47–51]. Consistent with the strategy adopted for all other cohorts, only non-redundant AML samples at diagnosis were selected for our analyses (*N* = 387) [52]. The raw (.CEL) files of the Verhaak cohort (GSE6891) were downloaded from the GEO database to process microarray datasets. The 461 patient specimens used in this study included both AML and MDS patients (indicated by the old nomenclature RAEB-T (refractory anemia with excess blasts (RAEB) or RAEB with transformation]). The count matrix of the available RNA-seq raw counts was analyzed using the DEseq2 R package. Raw (.CEL) files were normalized separately using the robust multichip average (RMA) implemented in R package affy [53]. Differential gene expression analysis was performed using the Bioconductor R software (R studio Version 4.3.1, 2024.09.0 + 375). Gene ontology (GO) enrichment analyses, including Biological Processes (BP), Molecular Functions (MF), and Cellular Components (CC), were computed using Bioconductor R software (packages; AnnotationDBI, clusterProfiler, org.Hs.eg.db) and significantly upregulated genes (*P* adj < 0.05) with a log2Foldchange > 0.5.

To investigate the metabolomic differences among the AML cell lines, we utilized the Cancer Cell Line Encyclopedia (CCLE) dataset obtained from the portal (https://depmap.org/portal/ccle/). The metabolite datasets of MV4-11 (Depmap ID: ACH000045) and U937 (Depmap ID: ACH000406) were examined to facilitate a comparative analysis.

### RNA‑Seq library preparation and sequencing

To capture gene alterations both preceding and during the execution of cell death, two distinct time points were selected for each cell line: 1 h after treatment to observe early molecular events prior to cell death initiation and a later time point prior to the onset of significant cell death: 6 h for MV4-11 and 48 h for U937 cells.

The total RNA concentration was calculated using Quant-IT RiboGreen (Invitrogen, #R11490). To assess the integrity of total RNA, samples were run on a TapeStation RNA screentape (Agilent, #5067–5576). Only high-quality RNA preparations with RIN greater than 7.0 were used for RNA library construction. A library was independently prepared with 1 µg of total RNA from each sample using an Illumina TruSeq Stranded mRNA Sample Prep Kit (Illumina, Inc., San Diego, CA, USA, #20020595). The first step in the workflow involved the purification of poly A-containing mRNA molecules using poly‐T‐attached magnetic beads. Following purification, the mRNA was fragmented into small pieces using divalent cations at elevated temperatures. Cleaved RNA fragments were copied into first-strand cDNA using SuperScript II reverse transcriptase ( #18064014; Invitrogen) and random primers, followed by second-strand cDNA synthesis using DNA Polymerase I, RNase H, and dUTP. These cDNA fragments then go through an end repair process, adding a single ‘A’ base and ligating the adapters. The products were purified and enriched by PCR to create the final cDNA library.

The libraries were quantified using KAPA Library Quantification kits for Illumina Sequencing platforms according to the qPCR Quantification Protocol Guide (KAPA BIOSYSTEMS, #KK4854) and qualified using TapeStation D1000 ScreenTape (Agilent Technologies, # 5067–5582). Indexed libraries were then submitted to Illumina NovaSeq (Illumina, Inc., San Diego, CA, USA), and paired-end (2 × 100 bp) sequencing was performed by Macrogen, Inc.

Raw sequencing reads in the FASTQ format were first assessed for quality using FastQC (version v0.12.1, Babraham Institute, UK). Low-quality bases and adapter sequences were removed using Trim Galore (v0.6.10; Babraham Institute, UK) to ensure that clean reads were obtained for further analysis. Trimmed sequencing data were aligned to the reference human genome (hg38) using HISAT2 (v. 2.2.1, Center for Computational Biology, Johns Hopkins University, MD, USA). The resulting SAM files were then converted to BAM format and sorted using SAMtools (v 1.21, Wellcome Sanger Institute, UK). Gene-level quantification was performed using FeatureCounts (v 1.5.3, University of South Carolina, USA) with GENCODE Release 38 as the reference annotation. Differential expression analysis was conducted using the DESeq2 package in R, utilizing gene count data calculated from feature counts. Genes with an adjusted *p*-value < 0.05 were considered significantly differentially expressed. Gene Ontology (GO) enrichment analysis was performed using the PANTHER classification tool. The top 100 enriched GO terms based on *p*-values were further summarized and visualized using REViGO to reduce redundancy and provide a clear representation of biological functions.

### Statistics

All data are expressed as mean ± SD. Statistical significance was evaluated using the Student’s *t*-test and one-way ANOVA, as indicated in the Figure legends. Post-hoc analyses were performed using GraphPad Prism 10 software (La Jolla, CA, USA). Statistical significance was reported as follows:* *P* < 0.05, **P < 0.01, ***P < 0.001, and ****P < 0.0001, with post-hoc analyses conducted using Dunnett’s or Tukey’s tests (see figure legends for details).

## Results

### OPA1 expression in AML patients and disease progression

**Fig. 1:**
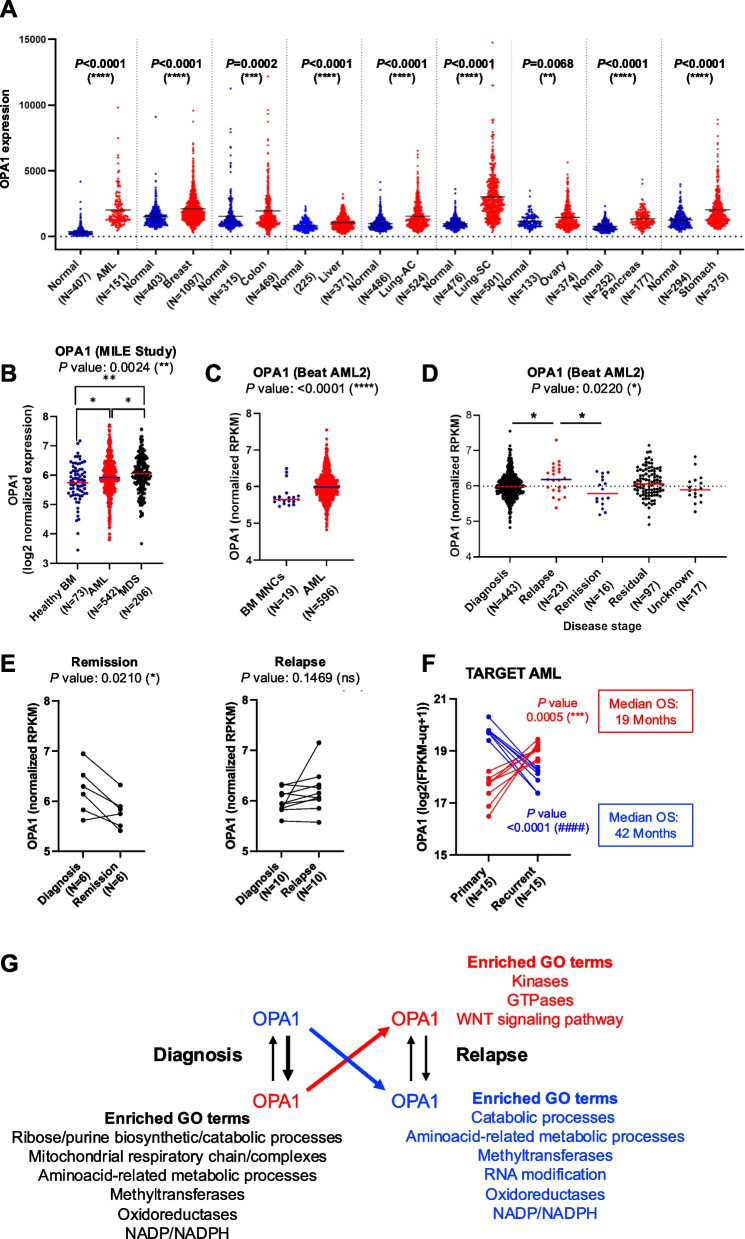
Pro-cancer features of increased OPA1 gene expression.** A** Differential gene expression of OPA1 in hematological and solid tumor patients was compared with healthy samples using data available from the TNMplot website. AC: adenocarcinoma, AML: acute myeloid leukemia, SC: squamous cell carcinoma. **B** Comparative analysis between specimens from healthy donors (BM: bone marrow; MNCs: mononuclear cells) and AML patients included in the MILE Study. The study also included samples available in the MILE Study from patients affected by myelodysplastic syndrome (MDS). **C** Comparative analysis between healthy donors and AML patients using Beat AML 2.0 cohorts. **D** OPA1 expression in AML patient samples from Beat AML 2.0 cohort stratified by the disease stage at the specimen collection. **E** Paired analysis of AML patient samples taken at the diagnosis and evolving to remission (left) or relapse (right) from the Beat AML 2.0 cohort. **F** Paired diagnostic vs. relapse analysis of 15 pediatric AML patients from the TARGET AML cohort showing distinct OPA1 expression trends between the two disease stages; median overall survival (OS) is reported on the right. **G** An overview of the gene ontology (GO) terms found to be enriched between selected patient groups of panel (**F**) is shown in Fig. S3. Red and blue colors of panels (**F**) and (**G**) identify the same groups of patients. In panel (**G**), the bolder arrows indicate the sample groups selected for the comparative analysis. GO terms enriched in a specific subgroup are reported in proximity. Red: GO terms enriched in relapsed AML samples with OPA1^high^ expression; Blue: GO terms enriched in relapsed AML samples with OPA1^low^ expression; black: GO terms enriched in diagnostic OPA1^high^ vs. diagnostic OPA1^low^ AML samples. Medians were compared by the Kruskal–Wallis test; comparisons between two subgroups or between the median of each subgroup and the overall median (dashed line) were performed applying the Mann–Whitney test (**A-D**) or paired t-test (**E**&**F**) (*P < 0.05; **P < 0.01, ***P < 0.001, ****P < 0.0001); ns: not significant

Given the importance of mitochondria in AML progression [54], we investigated the gene expression of mitochondrial dynamics factors, focusing on the pro-fusion factors OPA1 (Fig. 1A), mitofusin (MFN) 1 and 2, along with the pro-fission factors dynamin 1 like (DNM1L; encoding the protein DRP1), FIS1, and MFF, using the TNMplot database (Fig. S1A-E). Overall, OPA1 expression was significantly elevated in all cancers, with a 5.5-fold increase in AML compared to controls. DNM1L and MFF were also overexpressed in most tumor samples, including AML, relative to normal tissues. In AML cells, we observed a strong correlation between OPA1 and DNM1L expression (Fig. S1F). The expression of MFN1, MFN2, and FIS1 was also altered; MFN1 levels increased, whereas MFN2 and FIS1 levels decreased.

To validate these findings in AML, we examined gene expression in samples from the MILE Study and Beat AML 2.0, comparing healthy and AML patient data (Fig. 1B&C, Fig. S2A-B, and Table S4). Our results confirmed that OPA1 and DNM1L were consistently upregulated, whereas MFN1, MFN2, and FIS1 were downregulated, with no significant changes observed in MFF. Elevated OPA1 expression was also observed in MDS patients (Fig. 1B, Fig. S2A), suggesting that OPA1-targeted strategies may represent a promising therapeutic avenue for multiple cancer types, including hematological malignancies and AML.

Further analysis revealed that OPA1 expression levels varied according to disease stage. In samples from relapsed AML patients in Beat AML 2.0, OPA1 levels were significantly higher than in diagnostic or remission samples, with similar trends in DNM1L and FIS1 expression. Notably, the remission samples exhibited the most pronounced changes in gene expression, distinguishing them from the diagnostic and relapse stages (Fig. 1D, Fig. S2C, and Tables S5-6).

To assess whether lower OPA1 expression might correlate with favorable outcomes, we conducted paired analyses of different disease stages in Beat AML 2.0. Three comparisons (diagnostic vs. relapse, diagnostic vs. remission, and diagnostic vs. residual) indicated that OPA1 expression was significantly reduced in remission samples, with no significant differences in relapse or residual samples (Fig. 1E, Fig. S2D). This finding suggests that decreased OPA1 expression is associated with remission, although further studies are required to confirm this association.

Focusing on the pediatric TARGET AML cohort, we categorized the patients into OPA1^high^ and OPA1^low^ groups based on OPA1 changes at relapse (Fig. 1F). Although survival analysis lacked statistical significance due to the limited cohort size, the OPA1^low^ group exhibited a longer median overall survival (42 vs. 19 months) than the OPA1^high^ group (Fig. 1F, Table S7). Although not statistically significant, this trend suggested a potential link between low OPA1 expression and extended survival. DNM1L exhibited a similar modulation (Fig. S3A). Gene ontology (GO) analysis revealed enrichment of biosynthetic/catabolic and amino acid-related processes, oxidoreductases, and NADP/NAD(P)H in OPA1^low^ patients, irrespective of diagnosis or relapse stage (Fig. 1G, Fig. S3B-D). The OPA^low^ diagnostic group was enriched in mitochondrial respiratory chain processes compared with the OPA1^high^ diagnostic group. No mitochondrial or metabolic enrichment was detected in OPA1^high^ relapsed samples, suggesting a compensatory OPA1 mechanism during relapse to maintain metabolic stability. Finally, we assessed OPA1 expression across the AML cohorts, including cytogenetic risk, blast maturation stage, and FLT3 mutational status (Fig. S4A-C), without consistent modulation. Our bioinformatic analyses suggest that decreased OPA1 expression may correlate with a more favorable response in patients with AML. Given OPA1's role in mitochondrial fusion and fission, which are crucial for mitochondrial homeostasis [11], we further examined the effects of OPA1 modulation in AML cells.

### TMQ0153 induces mitochondrial dynamic alterations and morphological changes

**Fig. 2:**
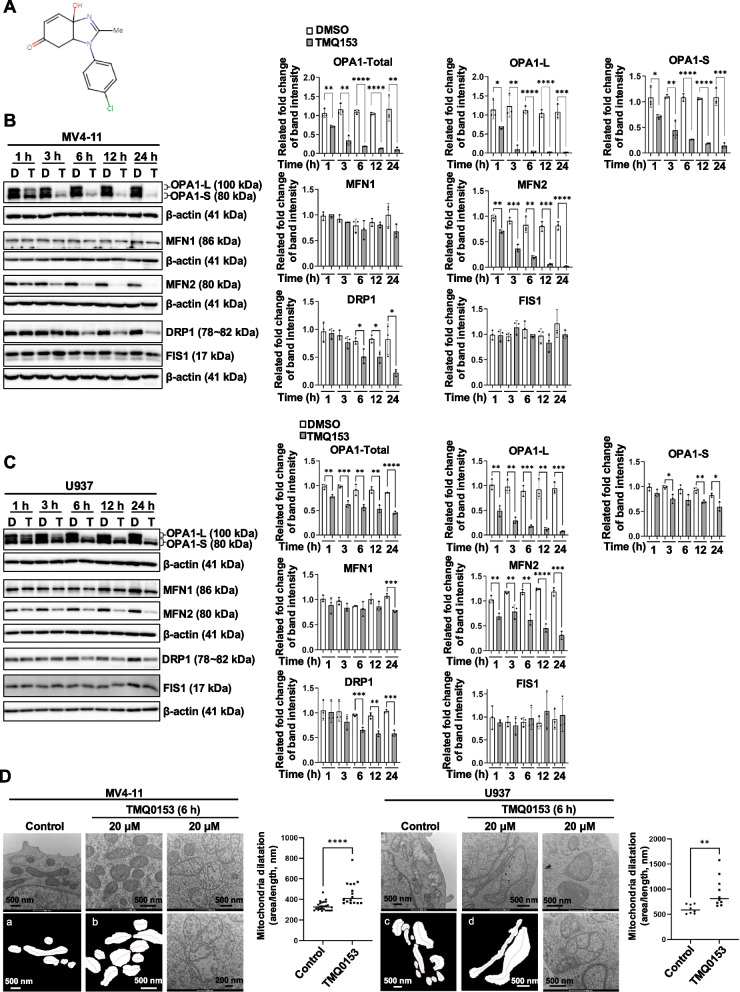
TMQ0153 alters the expression of mitochondrial dynamic-related proteins in AML cell lines. **A** Chemical structure of TMQ0153. **B** Western blot analysis of mitochondrial fission (DRP1 and FIS1) and fusion proteins (MFN1, MFN2, and OPA1) in MV4-11. **C** Western blot analysis of mitochondrial proteins in U937 cells. D: DMSO, T: 20 μM of TMQ0153 treatment. Immunoblot pictures (left panels) are representative of three independent experiments, and the quantification of the band intensity was performed using Image J program (right panels). **D** Representative images of electron microscopy pictures from MV4-11 and U937 cells treated as indicated in the figures (left panels) and the corresponding quantification of mitochondrial dilatation (right panels). The principle of the Area per Unit of Length (AUL) parameter was shown in a ~ d. Both cell lines show extreme dilatation (and cristolysis) in the mitochondria after TMQ0153 (20 µM) treatment. All data represent three independent experiments *P < 0.05, **P < 0.01, ***P < 0.001, ****P < 0.0001. Data were analyzed using unpaired two-tailed t-test (**B**&**C**) or Mann Whitney *t*-test (**D**)

Since mitochondrial dynamics encompasses the processes of fission, fusion, and metabolism [55], we investigated how tetrahydrobenzimidazole TMQ0153 (Fig. 2A) treatment alters mitochondrial dynamics in the AML cell lines MV4-11 and U937. TMQ0153 treatment significantly reduced the levels of mitochondrial fusion-related proteins within 1 h, including total OPA1 (1.5-fold), OPA1-long (1.7-fold), and OPA1-short (1.6-fold). MFN2 levels also showed a rapid reduction (1.4-fold) compared to untreated MV4-11 controls (Fig. 2B). In contrast, the mitochondrial fission protein DRP1 exhibited a 2.0-fold decrease after 6 h of treatment, whereas the FIS1 and MFN1 levels remained unchanged.

Similarly, in U937 cells, TMQ0153 treatment induced a rapid reduction in the OPA1 levels: total OPA1 (1.3-fold), OPA1-long (2.0-fold), and MFN2 (1.5-fold) within 1 h (Fig. 2C). DRP1 levels declined 1.8-fold after 6 h, with no significant effect on FIS1.

To examine the effect of TMQ0153 on mitochondrial morphology, we used TEM after 6 h of treatment 20 µM TMQ0153 of MV4-11 and U937 cells with 20 µM TMQ0153 (Fig. 2D). The effect on the mitochondrial network was quantified by measuring the area of the dilated mitochondria (white masks) per unit length (red lines). In MV4-11 cells, extreme mitochondrial dilation was observed following TMQ0153 treatment, whereas U937 cells showed enlarged and dilated mitochondria after 6 h (Fig. 2D). After 24 h of treatment with TMQ0153, the mitochondria exhibited shrinkage and disorganization of the mitochondrial matrix in MV4-11 and U937 cells (Fig. S5). Mitochondrial shrinkage is associated with ferroptosis, which is a form of programmed cell death [56].

Together, these findings demonstrate that TMQ0153 induces morphological changes in the mitochondrial network accompanied by an imbalance in mitochondrial fusion and fission proteins.

### TMQ0153 induced metabolic dysfunctions in AML cell lines

**Fig. 3:**
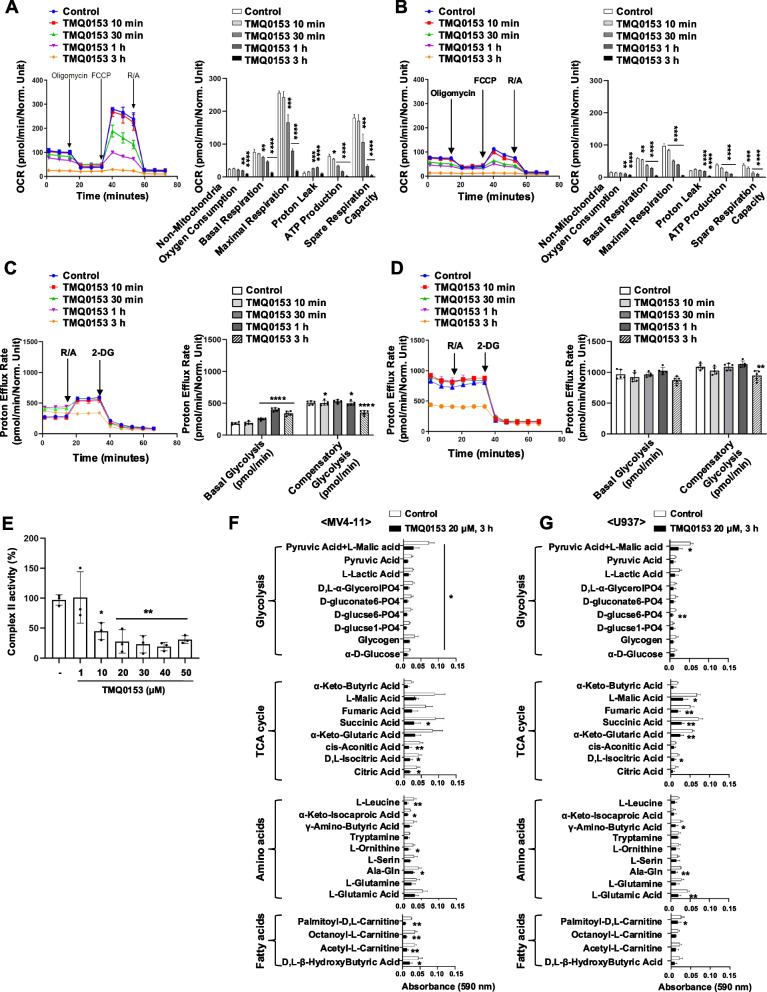
TMQ0153 inhibits mitochondrial respiration in AML and induces a metabolic shift in MV4-11 cells. **A-D** The metabolic profile of AML cells was evaluated using oxygen consumption rate (OCR) and proton efflux rate (PER) measurements to assess mitochondrial respiration and glycolysis, respectively. MV4-11 and U937 cells treated with 20 μM TMQ0153 for 10 min, 30 min, 1 h, and 3 h were analyzed using a Seahorse XFe96 analyzer. OCR levels (left panels) after the sequential addition of oligomycin (mitochondrial respiratory complex V inhibitor), carbonylcyanide p-trifluoromethoxyphenylhydrazone (FCCP; protonophore), and rotenone (R)/antimycin A (A) (mitochondrial respiratory complex I and III inhibitors, respectively) to analyze respiratory parameters (right panels) in MV4-11 (**A**) and U937 (**B**). The quantification graph on the right panel shows non-mitochondrial oxygen consumption, basal respiration, maximal respiration, proton leak, ATP production, and spare respiratory capacity. PER levels after the sequential addition of R/A and 2-deoxy-D-glucose (2-DG) to analyze the glycolytic parameters using a Glycolysis Rate Assay (GRA) in MV4-11 (**C**) and U937 cells (**D**). All values represent normalized (Norm.) units as computed by the Seahorse Wave software. **E** Mitochondrial respiratory complex II activity from isolated mitochondria exposed to increasing concentrations of TMQ0153. **F-G** Metabolic profile analysis using the Mitoplate™ S-1 assay was performed on MV4-11 (**F**) and U937 cells (**G**) after 3 h of treatment with 20 μM TMQ0153. All data represent three independent experiments. Statistical significance is indicated as *P < 0.05, **P < 0.01, ***P < 0.001, ****P < 0.0001 compared to control cells. Data were analyzed using one-way ANOVA with Dunnett’s test (**A–E**) and unpaired two-tailed t-test (**F**&**G**)

Since abnormalities in mitochondrial dynamics are linked to metabolic alterations [57] and given the importance of OPA1 in organizing the respiratory chain [58], we measured the mitochondrial oxygen consumption rate (OCR) after TMQ0153 treatment in MV4-11 (Fig. 3A) and U937 (Fig. 3B) cell lines.

In MV4-11 cells, ATP production (87.4%) was rapidly abrogated within 10 min of treatment. Basal respiration (84.1%), maximal respiration (71.0%), and spare respiratory capacity (65.7%) were significantly reduced after 30 min compared with the control (Fig. 3A). The proton leakage gradually increased and peaked after 1 h. After 3 h of TMQ0153 treatment, the OCR levels were completely abolished, indicating severe mitochondrial dysfunction.

After 10 min of treatment with U937, basal respiration (92.3%), maximal respiration (86.4%), ATP production (76.3%), and spare respiratory capacity (77.2%) were significantly reduced compared the control (Fig. 3B). MV4-11 cells exhibited high OCR levels, which are characteristic of relapse-initiating drug-tolerant persister cells (DTPs). Notably, after TMQ0153 treatment, a metabolic shift occurred in MV4-11 cells, with basal glycolysis significantly increasing, reaching a 2.1-fold increase after 1 h (Fig. 3C). In contrast, the U937 cells showed no comparable shift in glycolysis (Fig. 3D).

OCR levels were much lower in U937 cells than in MV4-11 cells, but the proton efflux rate (PER) was considerably higher in U937 cells. These results suggest that U937 cells rely more on glycolysis than on mitochondrial respiration.

We evaluated the effect of TMQ0153 on mitochondrial complex activity and found that it significantly reduced complex II activity at concentrations > 10 μM (Fig. 3E). The activity of complex I remained unaffected, and those of complexes II/III, IV, and V were significantly affected, but to a lesser extent (Fig. S6). Because complex II is crucial for respiratory capacity and links the TCA cycle to the ETC, targeting OXPHOS may help overcome drug resistance in relapsed AML [59, 60].

Next, we assessed the effect of TMQ0153 on the ability of the respiratory chain to convert energetic metabolites into efficient electron flux. In MV4-11 cells (Fig. 3F), TMQ0153 significantly impaired glycolysis and fatty acid metabolism. In addition, the conversion of several TCA cycle intermediates was reduced [succinic acid (-32.4%), cis-aconitic acid (-31.9%), D,L-isocitric acid (-42.9%), and citric acid (-44.8%)]. The U937 cells showed a significant decrease in glycolysis and TCA cycle intermediates (Fig. 3G).

RNA sequencing was used to analyze 69 genes in four categories (Metabolism, Mitochondria, OXPHOS, and ROS) in MV4-11 and U937 cells. We determined the treatment time after the assessment of cell death induction in both cell lines. MV4-11 cells underwent significant cell death after 6 h of treatment at 20 µM, whereas U937 cells required 48 h for comparable cytotoxic effects (Fig. S7). To assess early gene alterations and transcriptional changes preceding cell death, which are likely to cause subsequent cellular events, we harvested cells from both cell lines after one hour of treatment. We selected a second time point immediately preceding the onset of cell death, 6 h for MV4-11 and 48 h for U937 cells. This approach ensured the identification of key transcriptional changes relevant to the distinct kinetics of TMQ0153-induced cell death in each cell line. The heat map indicated reduced metabolic gene expression after 6 h of TMQ0153 treatment in MV4-11 cells (Fig. S8A-B). In contrast, these categories were not significantly affected in U937 cells. Tree and bubble plots of GO terms highlighted the most relevant biological processes and molecular functions affected by TMQ0153 treatment in MV4-11 (Fig. S9A-B) and U937 cells (Fig. S9C-D).

### TMQ0153-induced cell death in AML cell lines

Given the impact of TMQ0153 on mitochondrial function and OXPHOS, we further validated its anticancer effect by treating MV4-11 and U937 cells with different concentrations of TMQ0153 for up to 72 h. Trypan blue staining revealed dose- and time-dependent reductions in cell viability and proliferation in both cell lines (Fig. S7). In MV4-11 cells, viability significantly decreased at 10 μM (80.6%) after 72 h, whereas U937 cells showed a significant reduction at 20 μM (77.0%) after 48 h (Fig. S7A-B). Cytotoxic effects were observed at concentrations above 10 μM in both cell lines (Fig. S7C-D).

Cell death was quantified by annexin V/propidium iodide (PI) staining, with z-VAD-FMK (a pan-caspase inhibitor) used to assess the involvement of caspase-dependent apoptosis after 6 and 24 h of treatment (Fig. S7E–H). Pretreatment with z-VAD-FMK significantly restored the viability of MV4-11 cells treated with 10 μM TMQ0153, increasing it from 69.3% to 90.1% at 6 h (Fig. S7E). However, no rescue effect was observed in the U937 cells after 24 h of treatment (Fig. S7H). Etoposide (30 µM) was used as a positive control for the induction of cell death in both the MV4-11 and U937 cell lines. Additionally, midostaurin (500 nM), an FLT3-targeting drug, was used as a positive control to induce cell death, specifically in MV4-11 cells. The anti-leukemic effect of TMQ0153 was generalized through colony formation assays using the AML cell lines MV4-11, U937, HL60, and THP-1 (Fig. S10A-D).

### Docking simulation between OPA1 and TMQ compounds

We identified the crucial role of OPA1 using bioinformatic data (Fig. 1 and Fig. S1-4) and observed a significant reduction in OPA1 expression in AML cells after treatment with TMQ0153 (Fig. 2B-C). Based on this, we hypothesized that OPA1 is a molecular target of TMQ0153. P2Rank predicted four potential binding sites on a given protein structure, each of which was visualized using a surface representation (Fig. S11A). Binding probability reflects the likelihood of identifying a reliable site within the OPA1 protein, with values ranging from 0 to 1. The top-ranked GDP-binding site (colored red) had a probability of 0.858. The second site, colored green, had a probability of 0.291, while the third site, colored orange, had a probability of 0.163. The 4th site, depicted in magenta, has a probability of 0.042. Given the significantly higher probability of the top-ranked binding site, it is anticipated that the TMQ derivatives will bind at this location.

**Fig. 4:**
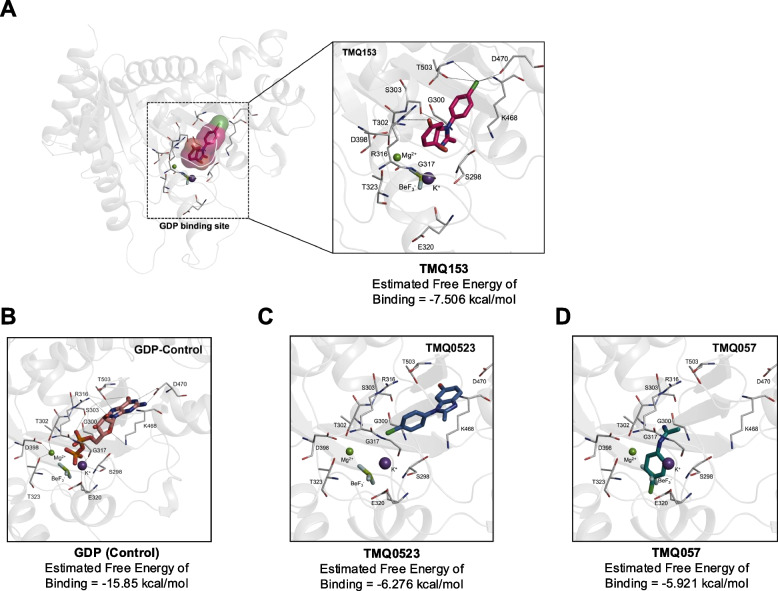
Structural analysis of the molecular docking simulations for TMQ candidates with OPA1 at the GDP binding site. **A** The optimal binding modes of TMQ0153 on OPA1 are illustrated in stick configuration. OPA1 residues involved in interactions are annotated in white. Dotted lines indicate potential interactions. Key cofactors, including Mg^2^⁺, K⁺, and BeF₃⁻, are colored in green, purple, and light green, respectively. **B-D** GDP (control) is represented in salmon, and various TMQ candidates are depicted in distinct colors. Detailed structural representations of the optimal binding models for GDP (**B**), TMQ057 (**C**), and TMQ0523 (**D**) with OPA1 are shown. The affinity energy was calculated by Vina

We conducted molecular docking simulations of various TMQ derivatives targeting the GDP-binding site of OPA1. Our findings linked the docking simulation results to the cytotoxic activity of the TMQ compounds, as shown by the IC50 analysis. Specifically, TMQ0153 emerged as the most potent cytotoxic compound, with the calculated binding free energy aligning with our expectations (Table S8). We used the GDP-bound OPA1 structure (PDB ID: 6JTG) for further molecular docking studies of the TMQ derivatives (Fig. 4A).

In these simulations, residues D470 and T503 formed ionic and polar interactions with the guanine base of GDP, whereas residues R316 and K468 engaged with the ribose ring of GDP within the nucleotide pocket. The presence of R316, K468, D470, and T503 in the upper portion of the nucleotide pocket creates a polar and ionic environment conducive to GDP binding. Conversely, residues S298, T302, G317, E320, T323, and D398 located in the lower portion of the pocket appeared to enhance OPA1’s enzymatic activity by interacting with catalytic ions rather than by direct binding (Fig. 4B) [61].

The TMQ0153-OPA1 (Fig. 4A) and TMQ0523-OPA1 (Fig. 4C) complexes showed higher binding energies than the TMQ057-OPA1 (Fig. 4D) complex, likely because of their positions in the nucleotide pocket. The chlorine atom in TMQ0153 forms a weak hydrogen bond with T503 and a noncovalent interaction with D470 [62]. Additionally, K468, with its positive charge, engages in a cation–π interaction with the aromatic ring of TMQ0153 (Fig. 4A) [63]. Thus, TMQ0153 was identified as the most potent cytotoxic compound, whereas TMQ057 showed no cytotoxic effect at the tested concentrations. These findings highlight OPA1's potential as a pharmacological target for TMQ0153.

To clarify the binding stability of ligands in the active site of the protein, we conducted molecular dynamics (MD) simulations for 500 ns (Fig. S11B). Analysis of ligand trajectories over time revealed distinct binding behaviors among the three ligands. Ligand TMQ057 dissociated from the binding site during the simulation, whereas ligands TMQ0153 and TMQ0523 remained bound throughout. This observation aligns well with the experimental IC50 values, where ligand TMQ057 exhibited the lowest inhibitory potency, suggesting that it had weaker binding interactions with the protein.

To assess the stability of ligand binding within the active site of the protein, we calculated RMSD values over time for TMQ153, TMQ523, and TMQ57 with OPA1. The results indicated that TMQ153 exhibited stable binding conformations, with RMSD values showing less fluctuation and remaining consistent throughout the simulation. This suggests that TMQ0153 maintains strong interactions with the binding site, supporting its structural stability. Overall, these findings indicate that TMQ153 and TMQ523 maintained stable binding to the protein, while TMQ57 demonstrated significant instability and dissociation from OPA1. These results highlight that the stability of ligand–protein binding is a key determinant of potential inhibitory activity, and TMQ57's weak binding may correspond to reduced biological efficacy.

Next, to assess the therapeutic potential of pharmacological targeting of OPA1, we knocked down OPA1 in K562 erythroleukaemia cells [41]. K562 cells exhibited a significant decrease in the total, long, and short-OPA1 (Fig. S12A), similar to the effects observed in the MV4-11 and U937 cells (Fig. S12B). Following siRNA transfection, the knockdown of OPA1 was confirmed by RT-PCR and western blotting (Fig. S13A&B). The OPA1 knockdown significantly impaired cell viability and proliferation (Fig. S13C and D), further validating OPA1 as a potential pharmacological target. Conversely, redox state and mitochondrial respiration were significantly affected by OPA1 knockdown (Fig. S13E&F). The level of Mcl-1, a protein that interacts with OPA1 in the inner mitochondrial membrane, was significantly reduced after the deletion of OPA1 (Fig. S13G), indicating its importance in maintaining oxidative phosphorylation and mitochondrial dynamics [64].

### TMQ0153-induced oxidative stress triggers DNA damage and intrinsic apoptosis

**Fig. 5:**
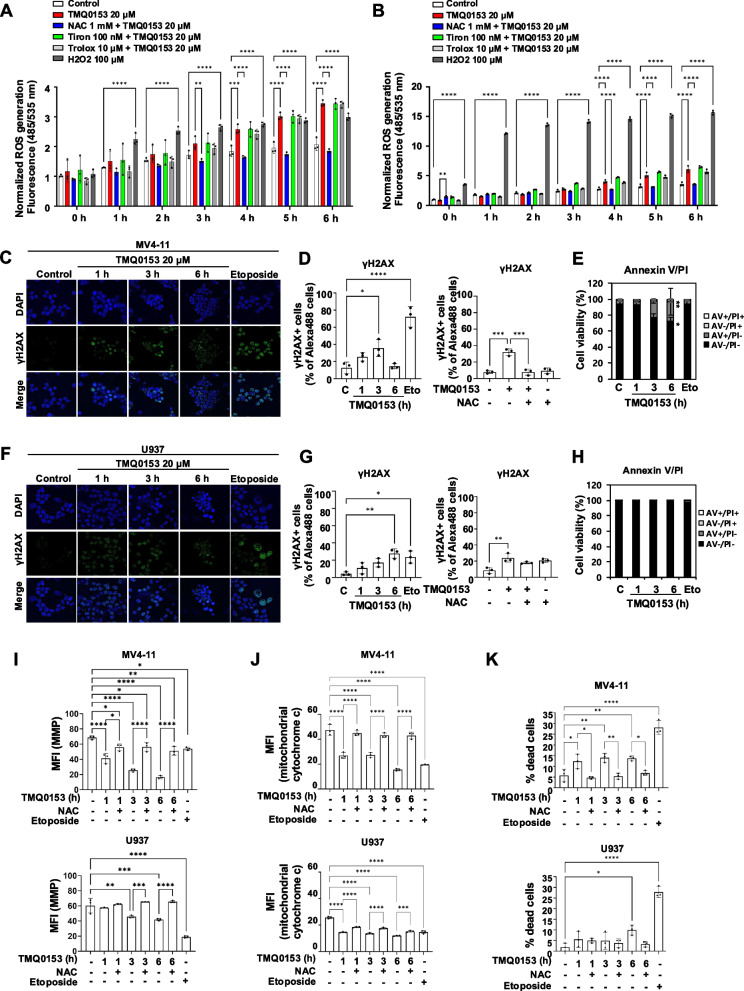
TMQ0153 induces ROS accumulation, increasing γH2AX, depolarizing MMP, and releasing cytochrome c, leading to apoptosis. **A-B** MV4-11 **(A)** and U937 **(B)** cells were pre-treated for 1 h with ROS scavengers (NAC, Tiron, and Trolox) before supplementing the medium with 20 μM TMQ0153. Then, DCF-DA fluorescence was measured for up to 6 h. **C** γH2AX (green) and DAPI (blue) signals were observed by confocal microscopy in MV4-11. **D** γH2AX levels were assessed by FACS over time (left) and after NAC pretreatment (1 mM, 1 h), followed by TMQ0153 exposure (3 h, 20 μM) (right). Eto: etoposide (30 μM, 6 h). **E** Cell viability was assessed by Annexin V (AV)/ propidium iodide (PI) staining in MV4-11 cells. **F** γH2AX was observed by confocal microscope using U937 cells treated with 20 μM TMQ0153. **G** γH2AX levels were monitored by FACS over time in U937 cells (left), with NAC’s prevention effect shown (right). **H** Corresponding cell viability was assessed by FACS. (**I**) Mitochondrial membrane potential (MMP) depolarization was assessed by FACS after TMQ0153 treatment (20 μM) in MV4-11 and U937 cells with NAC pretreatment (1 mM, 1 h). **J** Anti-cytochrome c-FITC was detected in MV4-11 and U937 cells following 20 μM TMQ0153 treatment. **K** Cell death was quantified by trypan blue staining. Etoposide (E30 μM, 24 h) served as a positive control. Data shown are from three independent experiments. Statistical significance is *P < 0.05, **P < 0.01, ***P < 0.001, ****P < 0.0001. Data were analyzed using one-way ANOVA using Tukey’s test

Considering the effect of TMQ0153 on mitochondrial dynamics, integrity, and metabolism, we investigated the involvement of ROS generation in TMQ0153-induced cell death. MV4-11 and U937 cells were pre-treated with three different ROS scavengers: NAC, Tiron, and Trolox (Fig. 5A and B, respectively). TMQ0153 significantly increased ROS levels by 1.4-fold after 4 h in both the cell lines. NAC effectively inhibited ROS generation, whereas Tiron and Trolox failed to reduce the ROS levels.

As ROS-induced free radicals can cause DNA strand breakage [65], we assessed DNA damage by monitoring the phosphorylation of the H2AX isoform (γH2AX), a marker for DNA double-strand breaks (DSBs). Flow cytometry showed a 2.8-fold increase in γH2AX expression within 3 h of TMQ0153 treatment, which decreased after 6 h (Fig. 5C-E). NAC pretreatment completely inhibited DNA damage and reduced γH2AX levels compared with those in the control group.

In U937 cells, γH2AX expression increased 6.4-fold after 6 h of TMQ0153 treatment at 20 µM (Fig. 5F-H). These results indicated that TMQ0153 induced intracellular ROS accumulation, DNA damage, and mitochondria-mediated cell death in both AML cell lines, albeit with differences in timing and extent.

To further explore the effect of NAC on TMQ0153-induced intrinsic cell death mechanisms, we evaluated mitochondrial membrane potential (MMP) depolarization in both cell lines with or without NAC pretreatment. In MV4-11 cells, NAC partially restored the decreased MMP caused by TMQ0153 (F ig. 5I). In U937 cells, a significant MMP reduction occurred only after 3 h of treatment, but NAC fully restored MMP to control levels.

TMQ0153 also triggered cytochrome c release in both the cell lines. After 1 h of treatment, mitochondrial cytochrome c levels decreased by 43.3% in MV4-11 cells and 42.9% in U937 cells compared to control. NAC pre-treatment effectively prevented the decrease in mitochondrial cytochrome c in MV4-11 cells, maintaining 94.8%, 91.0%, and 91.0% of the initial cytochrome c content in the control group after 1, 3, and 6 h, respectively. In U937 cells, NAC was less effective, preserving 72.3%, 68.78%, and 59.7% of cytochrome c content at the same time points as the control (Fig. 5J and Fig. S14). Cytochrome c and MitoTracker Green localization was examined by confocal microscopy (Fig. S15) to evaluate mitochondrial integrity and cytochrome c release. The MFI ratio of cytochrome c/MitoTracker Green was significantly reduced after 3 h of TMQ0153 (20 µM) treatment in MV4-11 (3.6-fold) and U937 cells (2.3-fold).

NAC pretreatment significantly prevented TMQ0153-induced cell death in MV4-11 cells at 1, 3, and 6 h (Fig. 5K). In contrast, U937 cells were less sensitive, with cell death (9.9%) delayed until 6 h. These findings suggest that NAC effectively preserves MMP, reduces cytochrome c release, and maintains MV4-11 cell viability. In U937 cells, although NAC delayed cell death, its reduced impact on MMP preservation indicated the differential ability of the two cell types to cope with oxidative stress. ROS generation contributes to TMQ0153-induced MMP depolarization and cell death via distinct detoxification mechanisms in AML cell lines.

### TMQ0153 induces redox state alterations and depletes GSH/GSSG and NAD^+^/NADH levels

**Fig. 6:**
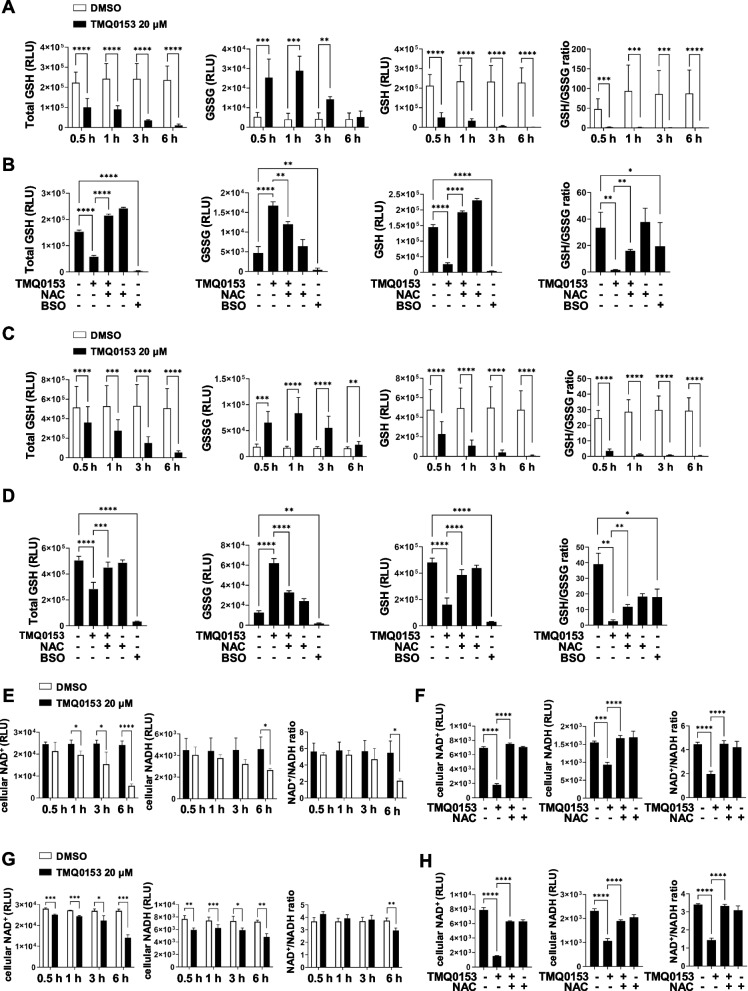
ROS-dependent changes in GSH/GSSG and NAD^+^/NADH ratios after TMQ0153 treatment in AML.** A** GSH/GSSG ratios were measured in MV4-11 after 20 μM of TMQ0153 treatment for 0.5 h, 1 h, 3 h, and 6 h. **B** N-acetylcysteine (NAC, 1 mM, 1 h) was pretreated following TMQ0153 (20 µM, 1 h) treatment in MV4-11, and GSH/GSSG ratios were assessed. Buthionine sulfoximine (BSO, 50 μM, 24 h) served as a negative control. **C** U937 cells were treated with 20 μM of TMQ0153 for up to 6 h, and GSH/GSSG ratios were assessed. **D** Pretreatment of NAC (1 mM, 1 h) mitigates the effect of TMQ0153 (20 µM, 1 h) on the GSH/GSSG ratio. **E** NAD^+^, NADH, and NAD^+^/NADH ratios were measured following 20 μM TMQ0153 treatment, with luminescence monitored for 6 h in MV4-11. **F** NAC (1 mM, 1 h) pretreatment was applied to assess its effect on NAD.^+^ metabolism in MV4-11. **G** U937 were utilized to assess NAD + , NADH, and the NAD + /NADH ratio after treatment with 20 μM TMQ0153. **H** The rescue effect of NAC (1 mM, 1 h) was verified in U937 cells after TMQ0153 (20 µM, 1 h) treatment. Data are presented as mean ± SD of three independent experiments). Statistical significance is denoted as *P < 0.05, **P < 0.01, ***P < 0.001, ****P < 0.0001 compared to the control group. *P*-values were calculated by unpaired two-tailed* t*-tests (**A**, **C**, **E**, and **G**) and one-way ANOVA using the Tukey Multiple Comparison test (**B**, **D**, **F**, and **H**)

We observed that TMQ0153 induced varying levels of ROS involvement in AML cell death (Fig. 5A-B). To assess redox changes, we measured the total GSH, reduced GSH, oxidized GSSG, and the GSH/GSSG ratio in MV4-11 (Fig. 6A-B) and U937 cells (Fig. 6C-D). A decreased GSH/GSSG ratio indicates oxidative stress, and GSH depletion often leads to apoptosis due to cytotoxic agents or environmental stress.

In MV4-11 cells, TMQ0153 treatment reduced total GSH (46.0%), GSH (24.8%), and the GSH/GSSG ratio (5.7%) within 30 min compared with the control (Fig. 6A). In line with the increase in ROS, GSSG level, a marker of ROS generation, increased 5.8-fold within 30 min of treatment and peaked after 1 h. Subsequently, GSSG levels declined in a time-dependent manner. NAC pre-treatment effectively prevented GSH depletion and GSSG accumulation in MV4-11 cells (Fig. 6B).

In U937 cells, TMQ0153 triggered a significant depletion of total GSH (52.4%) after 1 h of treatment. Reduced GSH levels (46.3%) and the GSH/GSSG ratio (13.5%) also decreased significantly within 30 min (Fig. 6C). NAC pre-treatment restored GSH levels and reduced GSSG accumulation in U937 cells (Fig. 6D). Buthionine sulfoximine (BSO) was used as the positive control. A heat map displaying cell line metabolite data from CCLE (Fig. S16) indicated that the oxidized and reduced GSH levels were higher in U937 cells than in MV4-11 cells. This observation suggests distinct variations in the redox regulation between the two cell lines.

The NAD^+^/NADH ratio is critical in maintaining redox homeostasis and is a key indicator of cellular health and mitochondrial metabolism [66, 67]. AML cells were treated with 20 µM TMQ0153 for 6 h, and NAD^+^ and NADH levels were measured. In MV4-11 cells, NAD^+^ levels decreased significantly (86.6%) after 1 h, whereas NADH (59.3%) and the NAD^+^/NADH ratio (40.6%) were significantly reduced after 6 h of treatment compared to control (Fig. 6E). NAC pre-treatment fully restored NAD^+^, NADH, and the NAD^+^/NADH ratio to baseline levels (Fig. 6F). In U937 cells, TMQ0153 treatment significantly reduced NAD^+^ (89.7%) and NADH (77.2%) levels after 30 min compared with the control. However, the NAD^+^/NADH ratio did not change significantly changes until 6 h, when it reached 78.7%, as both NAD^+^ and NADH levels were reduced in parallel (Fig. 6G). NAC pretreatment successfully prevented NAD^+^, NADH, and NAD^+^/NADH ratio reductions in U937 cells (Fig. 6H).

Taken together, these results indicated that TMQ0153-induced ROS formation impaired redox metabolism in both MV4-11 and U937 AML cells, ultimately leading to cell death.

### TMQ0153 triggers intrinsic apoptotic mechanisms

As we observed caspase-dependent apoptotic cell death through annexin V/PI staining (Fig. S7G-H), we quantified the expression levels of proteins involved in the mitochondrial apoptotic pathway by western blot analysis. In U937 cells, Mcl-1 levels decreased after 3 h and 6 h of TMQ0153 treatment (1.3- and 1.3-fold, respectively), whereas in MV4-11 cells, Mcl-1 was reduced within 1 h (1.7-fold) (Fig. S17A).

Bcl-xL expression decreased significantly after 6 h of TMQ0153 treatment in both U937 and MV4-11 cells (1.7- and 3.3-fold, respectively), indicating that Mcl-1 and Bcl-xL were regulated by cell death. Bcl-2 levels remained unchanged. Additionally, TMQ0153 caused Bid truncation in U937 cells (19.9-fold) and a notable increase in MV4-11 cells at 3 h and 6 h (20.7- and 20.2-fold, respectively; Fig. S17A). Bid truncation led to ROS-dependent mitochondrial outer membrane permeabilization (MOMP) and cytochrome c release in MV4-11 and U937 cells at 3 and 6 h, respectively (F ig. 5I-J). We investigated caspase involvement in TMQ0153-induced cell death by measuring the activity of nine caspases, with and without z-VAD-FMK. In MV4-11 cells, TMQ0153 activated initiator caspases -2 (1.8-fold), -9 (1.3-fold), and -10 (1.9-fold), as well as executioner caspases -3 (2.8-fold) and -6 (1.7-fold). Caspase-4 activity also increased (1.8-fold) (Fig. S17B). In U937 cells, all caspases except -1 and -5 were activated, with caspase-3 showing the highest activation (1.7-fold) (Fig. S17C).

Western blot analysis confirmed the activation of caspase-3, -8, and -9 along with the cleavage of poly(ADP-ribose) polymerase 1 (PARP1) following TMQ0153 treatment. In MV4-11 cells, pro-caspase-8, -9, and -3 levels were reduced by 7.1-, 50.0-, and 3.6-fold, respectively, after 3 h compared with DMSO-treated controls (Fig. S17D). Full-length PARP1, a substrate of caspases, decreased 4.3-fold after 6 h, whereas cleaved PARP1 increased 7.1-fold within 3 h.

In U937 cells, pro-caspase-8 was reduced by 1.7-fold after 3 h, followed by reduction in pro-caspase-9, -3, and full-length PARP1 after 6 h (2.7-, 2.0-, and 4.4-fold, respectively; Fig. S17E). Pretreatment with z-VAD-FMK for 1 h rescued caspase-8, -3, and PARP1 cleavage in both cell lines after 6 h of TMQ0153 treatment (Fig. S17F), indicating caspase-dependent apoptosis in response to TMQ0153, albeit with different kinetics.

Before evaluating the in vivo anti-cancer effects of TMQ0153, the toxicity of various doses of the compound was assessed in key organs, including the heart, spleen, lungs, liver, and kidneys. Histological analysis revealed no structural or cellular damage at any of the tested doses (Fig. S18). TMQ0153 was initially tested at doses of 20 mg/kg and 50 mg/kg (Fig. S19). While the 20 mg/kg dose exhibited limited efficacy, the 50 mg/kg dose resulted in significant toxicity, leading to the death of treated mice. To balance efficacy and safety, we assessed the effect of an intermediate dose of 35 mg/kg in subsequent in vivo experiments (Fig. S20A). Treating Balb/c nude mice with TMQ0153 at 35 mg/kg, no significant differences in body weight were observed between the vehicle-treated (*n* = 8) and TMQ0153-treated groups (*n* = 8), indicating good tolerability (Fig. S20B&C).

Treatment with TMQ0153 at 35 mg/kg led to a significant reduction in tumor volume starting on day 6 (Fig. S20D&E). After sacrifice, the tumor volume and weight showed significant differences between the treated and control groups, confirming TMQ0153's anti-cancer potential (Fig. S20F-H). The liver damage markers, glutamic pyruvic transaminase (GTP) and glutamic oxaloacetic transaminase (GOT), kidney damage markers, blood urea nitrogen (BUN), and creatinine (CRE) showed no significant differences. Although CRE levels were statistically significant, they remained below 0.20 mg/dL, the lower detection limit (Fig. S20I). Tumor tissues were stained for H&E, Ki-67, and cleaved caspase-3 (Fig. S20J). Altogether, this preliminary study indicated TMQ0153 that 35 mg/kg as a suitable and efficient concentration that was used for subsequent in vivo approaches. For other compounds (gilteritinib, venetoclax, and azacitidine), we selected concentrations based on established literature-validated regimens and therapeutic windows. This information, along with relevant sources, is summarized in Table S10.

### The combined treatment of TMQ0153 and Gilteritinib demonstrates a synergistic effect in vitro and in vivo

**Fig. 7:**
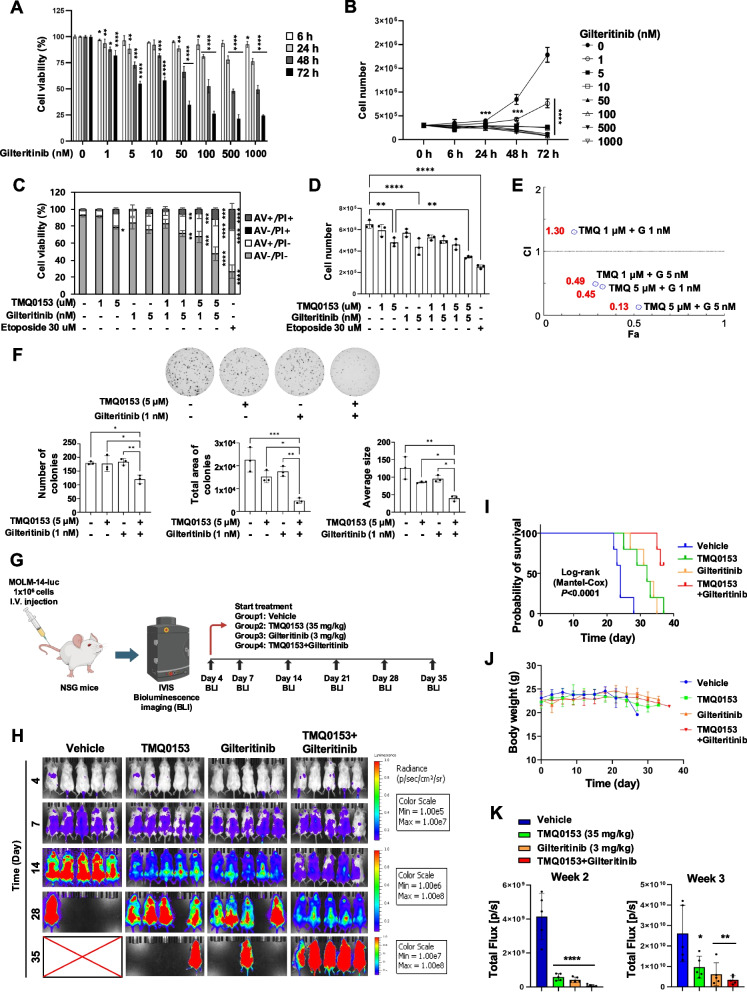
The synergistic effect of combination treatment with TMQ0153 and FLT3-targeting gilteritinib in the MV4-11 cell line and MOLM-14-luc xenograft study. **A** MV4-11 cells were treated with increasing concentrations of gilteritinib for up to 72 h, and cell viability was assessed using a trypan blue staining assay. **B** Cell proliferation following gilteritinib treatment. **C** Cell death was evaluated using AV/PI staining after combination treatment with TMQ0153 and gilteritinib. **D** Total cell numbers were measured by trypan blue staining after combination treatment. **E** The synergistic effect was analyzed using Compusyn software. **F** Colony formation assays were conducted following single or combination treatments in the MV4-11 cell line, with the number and total area of colonies indicated. **G** The scheme for the xenograft study using MOLM-14-luciferase (luc) cells. **H** Bioluminescence images, (**I)** survival curves, (**J)** body weight, and (**K)** total flux measurements in MOLM-14-luc-bearing NRG. TMQ0153 (35 mg/kg; intraperitoneal injection every 3 days) and gilteritinib (3 mg/kg; oral gavage daily) were administered alone or in combination. *N* = 5 mice/group. Statistical significance is denoted as *P < 0.05, **P < 0.01, ***P < 0.001, ****P < 0.0001. Data were analyzed using one-way ANOVA with Dunnett’s (**A, K**) and Tukey’s (**B**-**D** and **F**) Multiple Comparison tests. Survival curves were compared using the log-rank test (TMQ0153: *P* = 0.0072, gilteritinib: *P* = 0.0072, TMQ0153 + gilteritinib: *P* = 0.0021). A.u.: arbitrary units, CI: combination index, Fa: fraction affected, BLI: Bioluminescence imaging

Complex II activity is notably elevated in FLT3-mutated AML patients, and its inhibition sensitizes these blasts to apoptosis [68]. Consequently, we investigated whether TMQ0153 exhibits a synergistic effect when combined with gilteritinib in the FLT3-mutated AML cell line, MV4-11. Gilteritinib showed a cytotoxic effect and reduced the number of viable cells (Fig. 7A and B), with an IC50 of 15.4 nM after 72 h (Fig. S21). The combination treatment was evaluated by annexin V/PI staining and trypan blue exclusion tests after 24 h (Fig. 7C and D). The combination index (CI) and fraction affected (Fa) values were calculated according to the Chou-Talalay method (Fig. 7E). The strongest synergistic effect was observed with 5 μM TMQ0153 and 5 nM gilteritinib, yielding a CI of 0.13 (Table S9). A colony formation assay further confirmed a significant reduction in colony number, area, and size compared with the control and single-agent treatments (Fig. 7F).

We observed a similar synergistic effect in FLT3-mutated MOLM-14-luc cells derived from patients with relapsed AML (Fig. S21) in an orthotopic xenograft model of NSG mice (Fig. 7G). In vitro, this cell line demonstrated sensitivity to TMQ0153 compared to MV4-11 cells (Fig. S21). Similar to MV4-11, MOLM-14-luc cells harbored the FLT3-ITD mutation. Moreover, they also carried the KMT2A::MLLT3 fusion, representing a more genetically complex and aggressive AML phenotype. This genetic profile reflects the heterogeneity and therapeutic resistance commonly observed in clinical AML cases, which makes this model particularly valuable for preclinical studies. After injecting the cells, we assessed the anti-leukemic effects of TMQ0153 (35 mg/kg) and gilteritinib (3 mg/kg) (Fig. 7H). The single-agent treatments extended survival to 31.2 and 31.8 days, respectively, compared to 24.2 days in the vehicle group. The combination treatment significantly improved survival, with three out of five mice alive after 36 days (Fig. 7I) without significant changes in body weight (Fig. 7J). Tumor burden was significantly reduced (Fig. 7H-K). These xenograft results confirm the efficacy of TMQ0153 and its synergy with gilteritinib. By effectively targeting mitochondrial metabolism and FLT3 mutation, TMQ0153 is a promising strategy for relapsed AML. The drug dosage, administration methods, and schedules are listed in Table S10.

### Anti-leukemic effect of TMQ0153, Venetoclax, and Azacitidine combination on TP53-mutated cells

Patients with TP53 mutations often exhibit resistance to conventional therapies, making their treatment particularly challenging. Mutations in TP53 are linked to alterations in mitochondrial electron chain complex activity [69]. Supporting this, TP53 knockout has been shown to cause Venetoclax resistance, along with changes in mitochondrial homeostasis and cellular metabolism [70]. The combination of venetoclax and azacytidine reduces complex II activity in AML blast responders [71]. Additionally, inhibition of complex II overcomes venetoclax resistance [72]. Taken together, these findings suggest that targeting the mitochondrial electron transport chain, specifically complex II, could be an effective strategy to sensitize difficult-to-treat AML patient subgroups, such as those with TP53 alterations, to the venetoclax/azacytidine combination. As our studies showed TMQ0153’s ability to deplete glutathione and inactivate complex II, we tested the hypothesis that TMQ0153 could enhance the efficacy of the venetoclax and azacitidine combination.

**Fig. 8:**
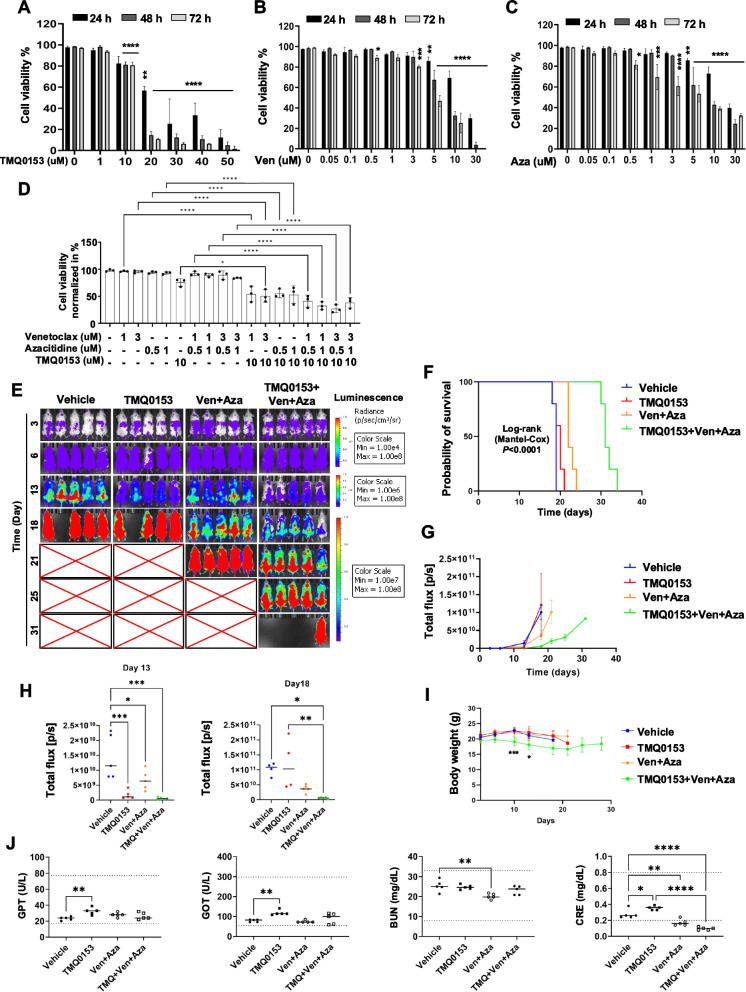
Anti-leukemic effect of combination treatment of TMQ053, venetoclax, and azacitidine. **A-C** U937-luc cells were treated with increasing concentrations of TMQ0153 (**A**), venetoclax (Ven, **B**), and azacitidine (Aza, **C**) for up to 72 h, and the cell viability was assessed by trypan blue staining assay. **D** The cytotoxic effect of combination treatment was estimated by trypan blue staining after 24 h of treatment. **E-J** U937-luciferase (luc) cells were injected into the tail vein of NSG mice. TMQ0153 (35 mg/kg; intraperitoneal injection; every 3 days), venetoclax (70 mg/kg; oral gavage, daily), and azacitidine (1.5 mg/kg, subcutaneously, daily) were administrated in combination (*n* = 5). Bioluminescence images (**E**), survivals (**F**), and total flux (**G**) were observed. The comparison of total flux at days 13 and 18 was shown (**H**). **I** The body weight of the mice was monitored throughout the treatment period. **J** Results of biochemical blood analyses for GPT, GOT, BUN, and CRE levels. The normal range of blood biochemistry was indicated by a dotted line. The result shown is the mean ± SD (*N* = 3) for (A ~ C) *P < 0.05, **P < 0.01, ***P < 0.001, ****P < 0.0001. Data were analyzed by One-Way ANOVA with Dunnett’s (**A-C**, **H**, and **I**) and Tukey’s (**D** & **J**) Multiple Comparison tests. Survival curve comparison was analyzed by the Log-rank test (**F**)

We evaluated the triple combination of TP53-mutated U937 and U937-luc cell lines in vitro and in vivo. Cell viability was assessed using trypan blue staining. The IC50 of venetoclax at 24 h was 8.8 ± 1.8 μM for U937 cells (Fig. S22) and 17.2 ± 2.2 μM for U937-luc cells (Fig. 8A-C and Fig. S23), indicating a greater sensitivity of U937 cells, consistent with prior findings that U937 cells express higher BCL-2 levels than U937-luc cells [52]. Conversely, U937-luc cells were more sensitive to azacitidine and TMQ0153 (azacitidine: 22.3 ± 1.5 μM, TMQ0153:22.1 ± 1.7 μM) compared to U937 cells (azacitidine: > 30 μM, TMQ0153: 37.7 ± 0.3 μM).

The synergistic effect of the triple combination was analyzed using the Compusyn software to identify the optimal effect of 10 μM TMQ0153 + 3 μM venetoclax + 0.5 μM azacitidine (Fig. 8D and Table S11). Although venetoclax/azacitidine treatment did not affect mitochondrial function, the addition of TMQ0153 significantly reduced maximal respiration and ATP production (Fig. S23).

In vivo studies involving U937-luc cells in NSG mice (Fig. 8E) demonstrated that triple combination treatment was associated with a notable improvement in survival, reaching an average of 31 days. This outcome was significantly better than that of the vehicle group, which had an average survival of 19 days, as well as the TMQ0153 group at 20 days and the venetoclax + azacitidine group at 22 days (Fig. 8F). The total flux assessments suggested that the triple combination regimen effectively hindered cancer cell proliferation compared to the vehicle and other treatment groups (Fig. 8G). The TMQ0153-treated group experienced a significant reduction in total flux by day 13; however, an increase in tumor levels was noted by day 18 (Fig. 8H). Additionally, body weight analyses indicated that both the TMQ0153-and Ven + Aza-treated groups showed similar weight maintenance as the vehicle group. In mice treated with a combination of TMQ0153, venetoclax, and azacitidine induced no significant weight loss (below 17% on day 10 and 14% on day13 of the relative body weight for vehicle animals) (Fig. 8I). While TMQ0153 treatment alone elevated the levels of organ damage markers (GTP, GOT, and CRE), they remained within normal ranges. CRE levels decreased in the venetoclax + azacitidine and triple combination groups, possibly indicating nutrient shortage, which is a potential side effect of venetoclax + azacitidine in NSG mice (Fig. 8J). Taken together, TMQ0153 shows promise as a combination therapy to restore drug sensitivity in TP53-mutated AML via metabolic interference.

## Discussion

Although treatment outcomes for certain AML subtypes have improved, the 5-year survival rate of adults with AML remains low at 30.5%) [2]. Adverse gene mutations, such as FLT3, KRAS, and TP53, contribute to treatment resistance, poor prognosis, and reduced overall survival [73]. FLT3 inhibitors have been developed since 2017; however, they have a short remission duration and can lead to drug resistance, posing challenges for AML treatment [74]. Mutations in TP53, RAS, and FLT3 are associated with adverse risk groups, and these patients derive limited benefits from the current therapies. Notably, elderly patients and those unfit for intensive chemotherapy have particularly poor outcomes.

Unfortunately, patients with TP53-mutated AML show minimal benefit from modern treatments, including venetoclax/azacytidine combinations, with overall survival of only 5.52 months. Therefore, novel pharmacological strategies are urgently needed to target TP53-mutant or FLT3-ITD AML cells, either alone or in combination with targeted therapies.

Recent studies have highlighted mitochondrial metabolism as a potential vulnerability factor in AML cells [54]. Mitocans, which target mitochondrial functions, have shown efficacy in AML cell lines such as MV-4–11, THP-1, OCI-AML2, and MOLM-13 [75]. For instance, combining mitocans, such as rotenone or etoposide, with the FLT3 inhibitor midostaurin produced synergistic effects, enhancing AML cell death [76].

OPA1, a key mitochondrial fusion regulator, is upregulated in multiple cancers, including AML and breast, colon, small cell lung, and stomach cancers (Fig. 1A). Deletion of OPA1 induces mitochondrial fragmentation and cell death, as demonstrated in MOLM-14 cells [77]. Notably, OPA1 expression in remission samples showed the most pronounced downregulation across various factors compared to diagnostic and relapsed AML samples (Fig. 1D, Fig. S2C, and Tables S5-6). Our analysis suggests that decreased OPA1 expression is associated with a more favorable response in AML patients. These findings highlight an association between OPA1 expression and adverse clinical outcomes. TMQ0153 damages mitochondria by initiating ROS accumulation, depolarizing mitochondrial membrane potential (MMP), and inhibiting mitochondrial respiration.

TMQ0153 significantly reduced OPA1 levels in MV4-11 cells within 1 h (Fig. 2B). In contrast, U937 cells showed persistence of the OPA1-short form (Fig. 2C), indicating greater resistance to TMQ0153. This aligns with the finding that the OPA1-short variant can preserve cristae structure, aiding cell survival under stress [78]. Mitochondrial cristae also regulate respiratory activity [14]. TMQ0153 caused significant mitochondrial dilatation, disrupting the ultrastructure of both the cell lines (Fig. 2D). The integrity of the mitochondrial ultrastructure is closely linked to the bioenergetic function and regulation of cytochrome c release, a key event in apoptosis [79].

Cellular metabolism modulation promotes tumor growth and drug resistance by helping cancer cells adapt to the tumor microenvironment (TME). For instance, treatment with the V-ATPase inhibitor archazolid A forces cancer cells to shift toward glycolysis for survival [80]. In MV4-11 cells, TMQ0153 treatment caused a similar metabolic shift, reducing cell viability (Fig. 3A and C). Conversely, U937 cells favored glycolysis, showing no significant metabolic changes until 6 h of treatment (Fig. 3B-D).

U937 cells exhibit a complex mutational profile, including the c.559 + 1G > A (p.Val173Trpfs*59) mutation that disrupts normal TP53 function, resulting in loss of tumor-suppressive activity and potential chemoresistance [81]. This mutation is particularly significant because TP53 alterations can influence AML cell responses to chemotherapy, potentially affecting the efficacy of drugs such as venetoclax and azacitidine [82].

TP53 protein plays a key role in apoptosis by directly translocating BAX to the outer mitochondrial membrane, activating the intrinsic apoptotic pathway [83]. In TP53 knockout mice, mitochondrial function and biomass are lost, whereas wild-type TP53 mice maintain mitochondrial biogenesis [84]. These findings explain the lower mitochondrial respiration capacity observed in TP53-mutated U937 cells (Fig. 3A-D) compared to TP53 wild-type MV4-11 cells.

TP53 regulates several key metabolic and apoptotic pathways, including the expression of cytochrome c oxidase 2 (SCO2) and glutaminase 2 (GLS2) [85], TP53 upregulated modulator of apoptosis (PUMA) [86], and the glucose transporters GLUT1 and GLUT4 [87]. TP53-induced glycolysis and apoptosis regulator (TIGAR) also modulates glucose metabolism [88]. When TP53 is mutated, repression of GLUT1 and GLUT4 is lost, leading to increased glycolysis, proliferation, and energy supply [89]. Thus, differences in mitochondrial morphology and cell survival in U937 cells likely resulted from the expression of the OPA1-short variant and TP53 mutation, which favors glycolysis. TMQ0153 specifically targeted mitochondrial respiration and disrupted the TCA cycle and fatty acid metabolism within 3 h in MV4-11 cells (Fig. 3F). This rapid metabolic dysfunction preceded cell death after 6 h of treatment.

Elevated ROS levels are a hallmark of leukemia cells, resulting from molecular mechanisms involving NADPH oxidase, the mitochondrial electron transport chain (mtETC), and cytochrome P450 [34, 90]. Impaired mitochondrial function exacerbates ROS production. TMQ0153 significantly increased the ROS levels in MV4-11 and U937 cells (Fig. 5A-B). Interestingly, NAC pre-treatment effectively reduced ROS levels, unlike trion and trolox. NAC aids in restoring intracellular glutathione levels and prevents free radical accumulation [91]. Elevated ROS can induce mutations and chromosomal instability by causing DNA double-strand breaks (DSBs), which trigger cell death if unrepaired [65].

TMQ0153 treatment induced γH2AX expression in MV4-11 cells, which was followed by a decrease, likely due to the onset of cell death (Fig. 5D-E). This suggests that TMQ0153-induced damage exceeded the DNA repair capacity of MV4-11 cells. In U937 cells, γH2AX levels increased until 6 h, without any reduction in cell viability (Fig. 5G). The absence of functional TP53 in U937 cells contributes to genomic instability [92] and impairs cell cycle checkpoints and apoptotic pathways [93]. This explains why apoptosis in U937 cells was delayed by 24 h compared to TP53 wild-type MV4-11 cells (Fig. S7B).

MV4-11 cells rely significantly on FLT3-ITD signaling [94] and have a relatively limited antioxidant capacity. This was evidenced by lower baseline levels of glutathione (GSH) [95], decreased activity of superoxide dismutase and catalase, and reduced expression of Nrf2-driven genes [96]. In contrast, U937 cells exhibit a much stronger defense against oxidative stress, such as nitric oxide (NO) [97], and possess more effective GSH-dependent detoxification pathways [98]. As a result, MV4-11 cells are more susceptible to accumulation of reactive oxygen species (ROS) and apoptosis when their survival signals are disrupted. In contrast, U937 cells not only neutralize ROS more effectively but also engage adaptive mechanisms, such as autophagy, to reduce oxidative damage [99].

GSH is a major ROS scavenger, whereas GSSG accumulation reflects oxidative stress [100]. The GSH/GSSG ratio is a key indicator of cellular redox balance. An elevated ratio reflects a reducing environment and a low ratio indicates oxidative stress [101]. A decline in the GSH/GSSG ratio is frequently observed prior to apoptosis [102]. In our study, both cell lines exhibited a rapid decrease in GSH/GSSG ratio after TMQ0153 treatment, indicating increased oxidative stress (Fig. 6A-D). A lower ratio suggests impaired antioxidant defenses, which can lead to lipid peroxidation, protein oxidation, and DNA damage, ultimately promoting apoptosis [103]. Pretreatment restored this effect, indicating that ROS generation reduced the GSH/GSSG ratio in AML cells (Fig. 5A-B, and Fig. 6B, D). Bioinformatics data showed that U937 cells had higher GSH levels than MV4-11 cells (Fig. S16).

NAD^+^ metabolism allows AML cells to resist apoptosis and contributes to Venetoclax resistance. NAD^+^ depletion triggers metabolic disruption, activates TP53, and induces cell death. The NAD^+^/NADH ratio is crucial for maintaining redox homeostasis and mitochondrial function [104]. Similar to its effect on GSH levels, TMQ0153 treatment reduced the NAD^+^/NADH ratio, which NAC could restored by NAC. These results indicate that TMQ0153-induced oxidative stress affects both the GSH/GSSG and NAD^+^/NADH ratios, leading to cell death in AML cells.

AML cells use multiple strategies to resist oxidative stress and chemotherapy, thereby promoting their survival and treatment resistance. These include upregulating antioxidant systems such as GSH [30], which neutralizes ROS and detoxifies drugs. ATP-binding cassette (ABC) transporters expel cytotoxic agents, thereby reducing their effectiveness [105], whereas cytochrome P450 enzymes generate less toxic metabolites [106]. Enhanced DNA repair mechanisms such as base excision repair (BER) and homologous recombination (HR) maintain genomic stability [107]. Additionally, transcription factor NRF2 boosts antioxidant genes, and metabolic reprogramming ensures a steady supply of NADPH for detoxification [108]. Together, these mechanisms enable AML cells to survive chemotherapy, and contribute to their aggressive nature and resistance to treatment.

Over the past few decades, the standard treatment for AML has remained largely unchanged, relying on a combination of cytarabine (Ara-C) and daunorubicin, known as the 7 + 3 regimen [109]. However, this approach presents significant challenges, particularly owing to the high toxicity in older patients and heterogeneity of the disease [110]. Furthermore, 30% of AML patients experience relapse and develop drug resistance driven by minimal residual disease (MRD), which is characterized by elevated mitochondrial metabolism and increased OXPHOS activity [4]. Targeting OXPHOS enhances drug sensitivity [60], indicating that TMQ0153 could be a promising component of combination therapy as mitocan.

Gilteritinib, a highly potent FLT3 inhibitor, has demonstrated promising clinical outcomes in adults with relapsed/refractory (R/R) FLT3-mutated AML [111]. Personalized chemotherapy approaches incorporating genomic profiling are becoming increasingly common for improving treatment response [112]. The *Beat AML® Master Clinical Trial* (NCT02927106) analyzed 562 patient specimens to advance the understanding of AML biology and develop new therapeutic targets.

AML cells demonstrate heightened sensitivity to mitocans compared to other solid tumors, supporting efforts to combine mitocans with FLT3 inhibitors to achieve synergistic cell death [75, 76]. Notably, a clinical trial (NCT02752035) is currently evaluating the combination of gilteritinib and azacitidine in 183 patients with newly diagnosed FLT3-mutated AML who are ineligible for intensive induction chemotherapy, with promising results showing significantly higher composite CR rates.

Our findings are consistent with these observations, showing a synergistic effect when TMQ0153 was combined with gilteritinib in FLT3-mutated cells (Fig. 7), and with venetoclax + azacitidine in TP53-mutated cells (Fig. 8). These preclinical results provided strong evidence that TMQ0153, as a standalone or in combination, holds substantial promise as an effective mitocan therapy for AML.

Although our study highlighted the promising therapeutic potential of TMQ0153 in AML, several limitations should be acknowledged. First, preclinical findings are primarily based on in vitro and xenograft models, which do not fully replicate the complexity of human AML, including interactions within the tumor microenvironment. Second, the differential response of MV4-11 and U937 cells underscores the heterogeneity of AML, which warrants future generalizability of the results to other AML subtypes. Additionally, while TMQ0153 demonstrated efficacy in FLT3- and TP53-mutated AML, its therapeutic impact on other adverse-risk mutations (e.g., NPM1 and RUNX1) needs to be further explored. Furthermore, mutational analysis to predict the potential binding sites of TMQ0153 on OPA1, as well as validation in AML-specific cell models, will provide more robust insights into its mechanism of action. Moreover, our reliance on specific functional assays, such as flow cytometry and Seahorse metabolic analyses, will be complemented in the future by spatial metabolomics-based approaches to elucidate the molecular mechanisms of TMQ0153 comprehensively. Further validation in patient-derived xenograft (PDX) models will pave the way for clinical trials, which are essential to confirm the safety, efficacy, and translatability of TMQ0153 as a standalone or combination therapy for AML treatment.

## Conclusion

TMQ0153 demonstrates significant potential as a therapeutic strategy for AML, particularly by targeting mitochondrial function and overcoming treatment resistance. By inhibiting OPA1, TMQ0153 induces mitochondrial dysfunction, metabolic reprogramming, and apoptosis in AML cells. The ability of a drug to increase ROS production, disrupt mitochondrial respiration, and alter key metabolic pathways, including the NAD + /NADH and GSH/GSSG ratios, enhances its effectiveness. When combined with existing therapies such as gilteritinib or venetoclax/azacitidine, TMQ0153 shows synergistic effects, offering a promising combination approach to improve treatment outcomes in AML. These preclinical findings support its potential as a powerful addition to AML therapy, particularly in resistant and high-risk patient subgroups.

## Supplementary Information

Supplementary Material 1.

Supplementary Material 2.

## Data Availability

RNA sequencing data can be accessed at the Gene Expression Omnibus (GEO292091).

## Abbreviations

2-DG: 2-Deoxy-D-glucose
ADP: adenosine Diphosphate
AML: acute myeloid leukemia
ATP: adenosine triphosphate
AUL: area per Unit of Length
Bcl-xL: B-cell lymphoma-extra large
BLI: bioluminescence imaging
BSO: buthionine sulfoximine
BUN: blood urea nitrogen
CML: chronic myeloid leukemia
CRc: composite complete remission
CREA: creatinine
DCFDA: 5-(and-6)-Carboxy-2',7'-Dichlorofluorescein Diacetate
DNA: deoxyribonucleic acid
DRP1: dynamin-related protein 1
DSB: DNA double-strand break
ECL: enhanced luminol-based chemiluminescent
ELN: European LeukemiaNet
FAB: French-American-British
FACS: fluorescence-activated cell sorting
FBS: fetal Bovine Serum
FCCP: carbonyl cyanide-4-(trifluoromethoxy) phenylhydrazone
FIS1: mitochondrial fission protein
FLT3: Fms-like tyrosine kinase
GDP: guanosine diphosphate
GLS2: glutaminase 2
GLUT1: glucose transporter 1
GLUT4: glucose transporter 4
GOT: glutamic-oxaloacetic transaminase
GPx: glutathione peroxidase family
GR: GSSG reductase
GSH: glutathione
GSSG: Oxidized GSH
GTP: glutamic pyruvic transaminase
i.p.: intraperitoneal
IHC: Immunohistochemistry
ITD: internal tandem duplication
KRAS: Kirsten rat sarcoma viral oncogene homolog
Mff: mitochondrial fission factor
MFI: median fluorescence intensity
MFN1: Mitofusin 1
MFN2: Mitofusin 2
MMP: Mitochondrial membrane potential
MOMP: mitochondrial outer membrane permeabilization
mtETC: mitochondrial electron transport chain
NAC: N-acetyl-L-cysteine
NAD^+^: Nicotinamide adenine dinucleotide
NADH: Reduced NAD
NADPH: nicotinamide adenine dinucleotide phosphate
NOD-SCID: nonobese diabetic/severe combined immunodeficiency
NSG: NOD-SCID gamma
OCR: oxygen consumption rate
OMM: Outer mitochondrial membrane
OPA1: Optic Atrophy 1
OXPHOS: oxidative phosphorylation
PARP1: poly(ADP-ribose) polymerase 1
PDB: protein data bank
PER: proton efflux rate
PRDXs: peroxiredoxins
PUMA: p53 up-regulated modulator of apoptosis
PVDF: Polyvinylidene fluoride
RNA: Ribonucleic acid
ROS: reactive oxygen species
RPMI 1640: Roswell Park Memorial Institute
SCO2: cytochrome c oxidase 2
SOD: superoxide dismutase
TCA: tricarboxylic acid
TEM: transmission electron microscopy
TIGAR: TP53-induced glycolysis and apoptosis regulator
TME: tumor microenvironment
TMQ: tetrahydrobenzimidazole
TP53: transformation-related protein 53
Z-VAD-FMK: carbobenzoxy-valyl-alanyl-aspartyl-[O-methyl]-fluoromethylketone
γH2AX: γ-phosphorylated form of histone H2AX
