# Supplementary material for: Tetrahydrobenzimidazole TMQ0153 targets OPA1 and restores drug sensitivity in AML via ROS-induced mitochondrial metabolic reprogramming
Source: J Exp Clin Cancer Res. 2025 Apr 7;44:114. doi: 10.1186/s13046-025-03372-0 (PMC11974110; doi:10.1186/s13046-025-03372-0)

Fig. S1

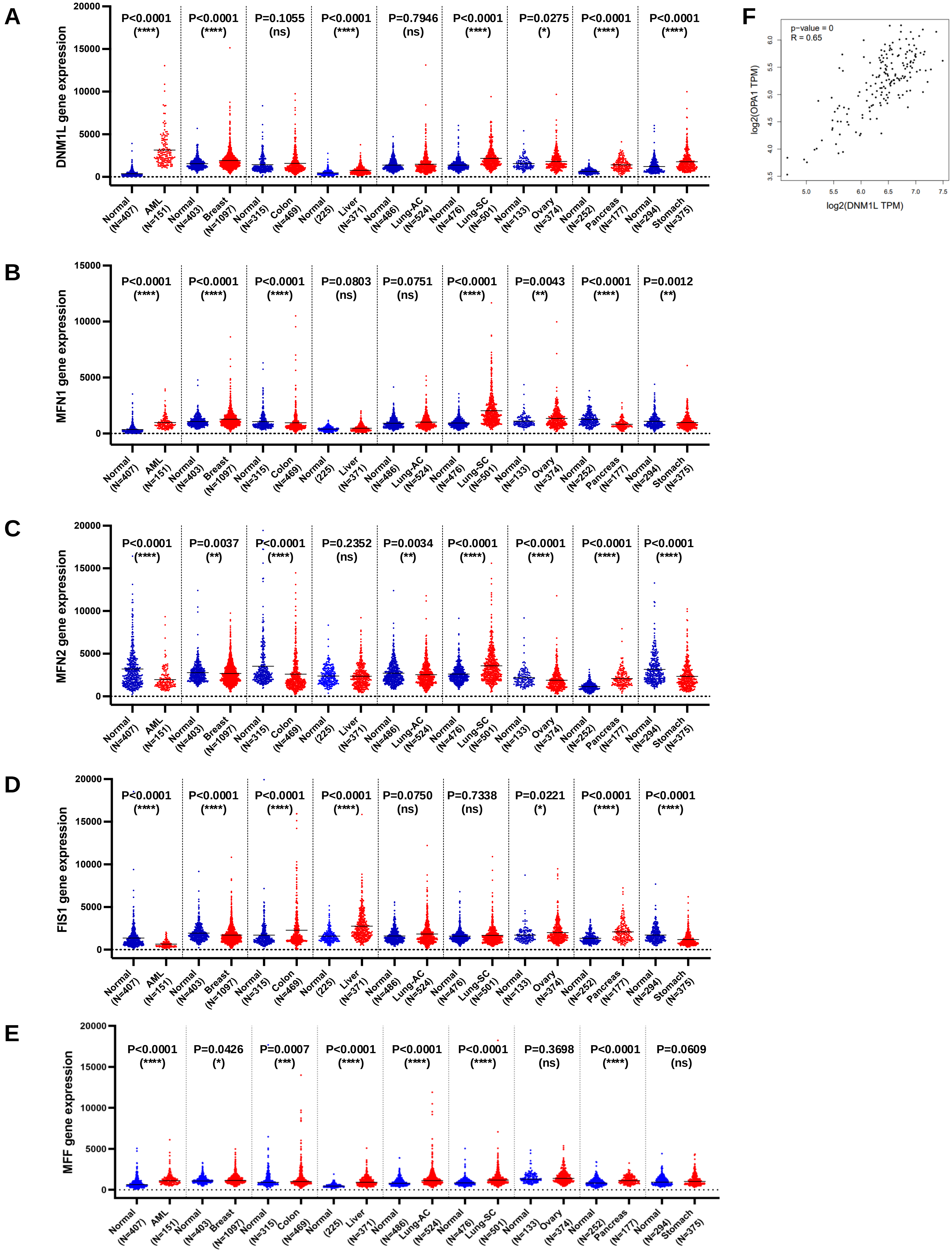
A
DNM1L gene expression
20000
15000
10000
5000
0
P<0.0001 (****)
P<0.0001 (****)
P=0.1055 (ns)
P<0.0001 (****)
P=0.7946 (ns)
P<0.0001 (****)
P=0.0275 (*)
P<0.0001 (****)
P<0.0001 (****)
Normal (N=407)
AML (N=151)
Normal (N=403)
Breast (N=1097)
Normal (N=315)
Colon (N=469)
Normal (225)
Liver (N=371)
Normal (N=486)
Lung-AC (N=524)
Normal (N=476)
Lung-SC (N=501)
Normal (N=133)
Ovary (N=374)
Normal (N=252)
Pancreas (N=177)
Normal (N=294)
Stomach (N=375)
F
p−value = 0
R = 0.65
log2(OPA1 TPM)
3.5
4.0
4.5
5.0
5.5
6.0
log2(DNM1L TPM)
5.0
5.5
6.0
6.5
7.0
7.5
B
MFN1 gene expression
15000
10000
5000
0
P<0.0001 (****)
P<0.0001 (****)
P<0.0001 (****)
P=0.0803 (ns)
P=0.0751 (ns)
P<0.0001 (****)
P=0.0043 (**)
P<0.0001 (****)
P=0.0012 (**)
Normal (N=407)
AML (N=151)
Normal (N=403)
Breast (N=1097)
Normal (N=315)
Colon (N=469)
Normal (225)
Liver (N=371)
Normal (N=486)
Lung-AC (N=524)
Normal (N=476)
Lung-SC (N=501)
Normal (N=133)
Ovary (N=374)
Normal (N=252)
Pancreas (N=177)
Normal (N=294)
Stomach (N=375)
C
MFN2 gene expression
20000
15000
10000
5000
0
P<0.0001 (****)
P=0.0037 (**)
P<0.0001 (****)
P=0.2352 (ns)
P=0.0034 (**)
P<0.0001 (****)
P<0.0001 (****)
P<0.0001 (****)
P<0.0001 (****)
Normal (N=407)
AML (N=151)
Normal (N=403)
Breast (N=1097)
Normal (N=315)
Colon (N=469)
Normal (225)
Liver (N=371)
Normal (N=486)
Lung-AC (N=524)
Normal (N=476)
Lung-SC (N=501)
Normal (N=133)
Ovary (N=374)
Normal (N=252)
Pancreas (N=177)
Normal (N=294)
Stomach (N=375)
D
FIS1 gene expression
20000
15000
10000
5000
0
P<0.0001 (****)
P<0.0001 (****)
P<0.0001 (****)
P<0.0001 (****)
P=0.0750 (ns)
P=0.7338 (ns)
P=0.0221 (*)
P<0.0001 (****)
P<0.0001 (****)
Normal (N=407)
AML (N=151)
Normal (N=403)
Breast (N=1097)
Normal (N=315)
Colon (N=469)
Normal (225)
Liver (N=371)
Normal (N=486)
Lung-AC (N=524)
Normal (N=476)
Lung-SC (N=501)
Normal (N=133)
Ovary (N=374)
Normal (N=252)
Pancreas (N=177)
Normal (N=294)
Stomach (N=375)
E
MFF gene expression
20000
15000
10000
5000
0
P<0.0001 (****)
P=0.0426 (*)
P=0.0007 (***)
P<0.0001 (****)
P<0.0001 (****)
P<0.0001 (****)
P=0.3698 (ns)
P<0.0001 (****)
P=0.0609 (ns)
Normal (N=407)
AML (N=151)
Normal (N=403)
Breast (N=1097)
Normal (N=315)
Colon (N=469)
Normal (225)
Liver (N=371)
Normal (N=486)
Lung-AC (N=524)
Normal (N=476)
Lung-SC (N=501)
Normal (N=133)
Ovary (N=374)
Normal (N=252)
Pancreas (N=177)
Normal (N=294)
Stomach (N=375)

Fig. S2

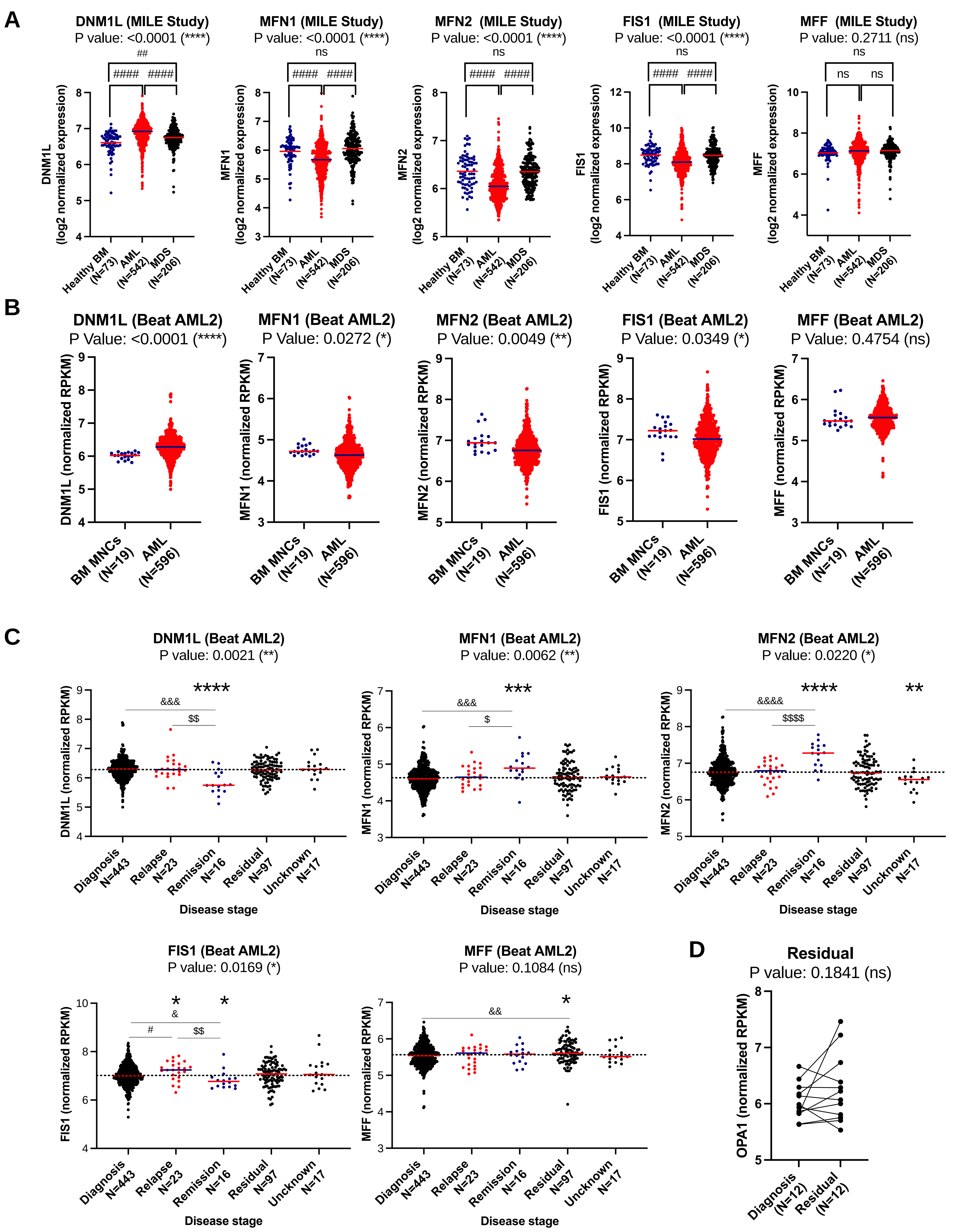

A
DNM1L (MILE Study)
P value: <0.0001 (****)
MFN1 (MILE Study)
P value: <0.0001 (****)
MFN2 (MILE Study)
P value: <0.0001 (****)
FIS1 (MILE Study)
P value: <0.0001 (****)
MFF (MILE Study)
P value: 0.2711 (ns)
Healthy BM (N=73)
AML (N=542)
MDS (N=206)
(log2 normalized expression)
B
DNM1L (Beat AML2)
P Value: <0.0001 (****)
MFN1 (Beat AML2)
P Value: 0.0272 (*)
MFN2 (Beat AML2)
P Value: 0.0049 (**)
FIS1 (Beat AML2)
P Value: 0.0349 (*)
MFF (Beat AML2)
P Value: 0.4754 (ns)
BM MNCs (N=19)
AML (N=596)
C
DNM1L (Beat AML2)
P value: 0.0021 (**)
MFN1 (Beat AML2)
P value: 0.0062 (**)
MFN2 (Beat AML2)
P value: 0.0220 (*)
FIS1 (Beat AML2)
P value: 0.0169 (*)
MFF (Beat AML2)
P value: 0.1084 (ns)
Diagnosis N=443
Relapse N=23
Remission N=16
Residual N=97
Unknown N=17
Disease stage
D
Residual
P value: 0.1841 (ns)
OPA1 (normalized RPKM)
Diagnosis (N=12)
Residual (N=12)

Fig. S3

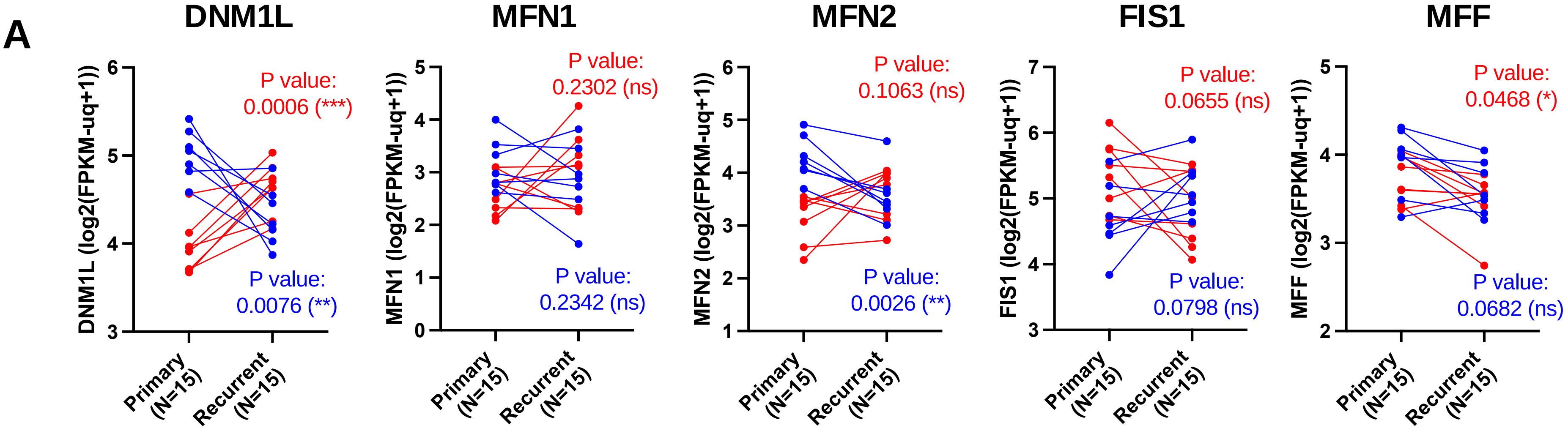
A
DNM1L
MFN1
MFN2
FIS1
MFF
DNM1L (log2(FPKM-uq+1))
MFN1 (log2(FPKM-uq+1))
MFN2 (log2(FPKM-uq+1))
FIS1 (log2(FPKM-uq+1))
MFF (log2(FPKM-uq+1))
P value: 0.0006 (***)
P value: 0.0076 (**)
P value: 0.2302 (ns)
P value: 0.2342 (ns)
P value: 0.1063 (ns)
P value: 0.0026 (**)
P value: 0.0655 (ns)
P value: 0.0798 (ns)
P value: 0.0468 (*)
P value: 0.0682 (ns)
Primary (N=15)
Recurrent (N=15)

Fig. S3 continued

B Top 20 enriched Gene Ontology terms in diagnostic OPA1^{low} TARGET AML

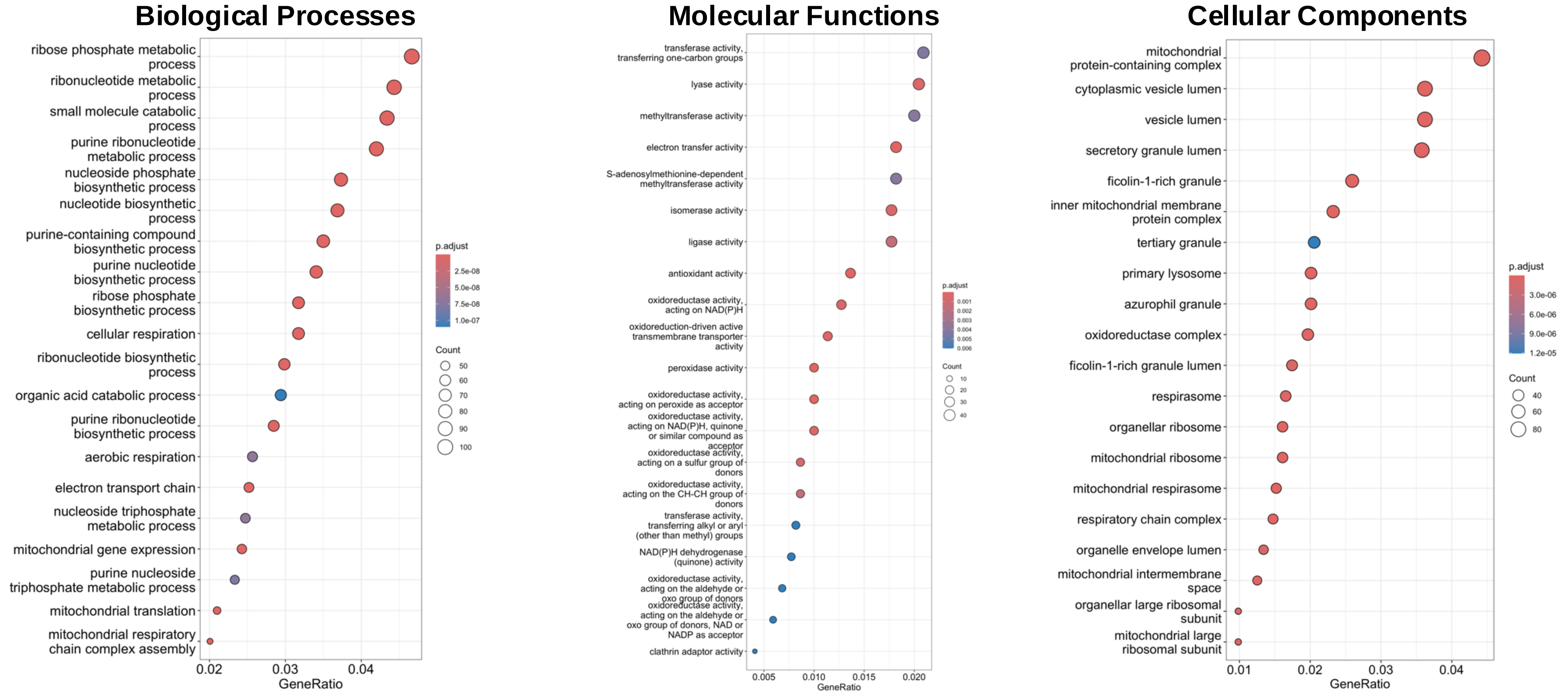

C Top 20 enriched Gene Ontology terms in relapsed OPA1^{high} TARGET AML (paired analysis)

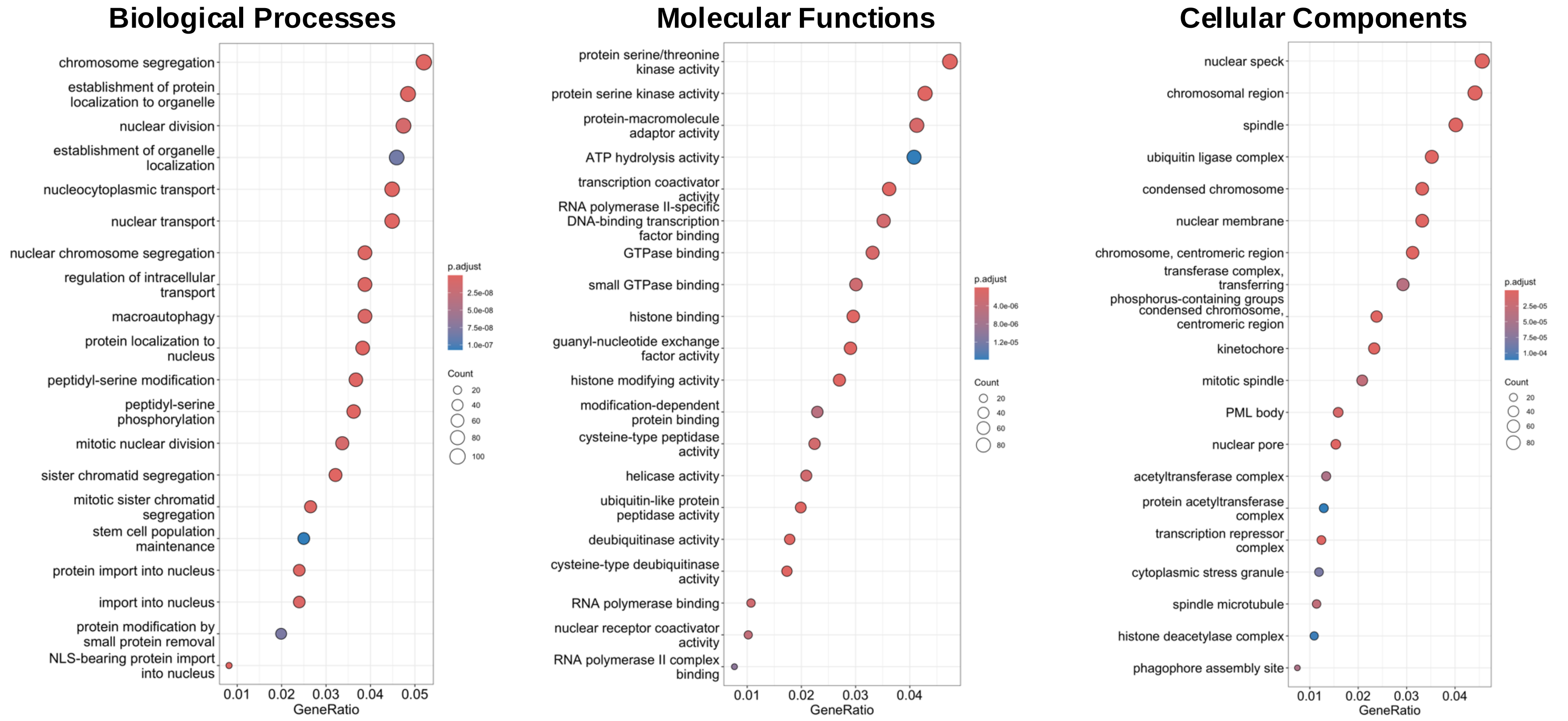

D Top 20 enriched Gene Ontology terms in relapsed OPA1^{low} TARGET AML (paired analysis)

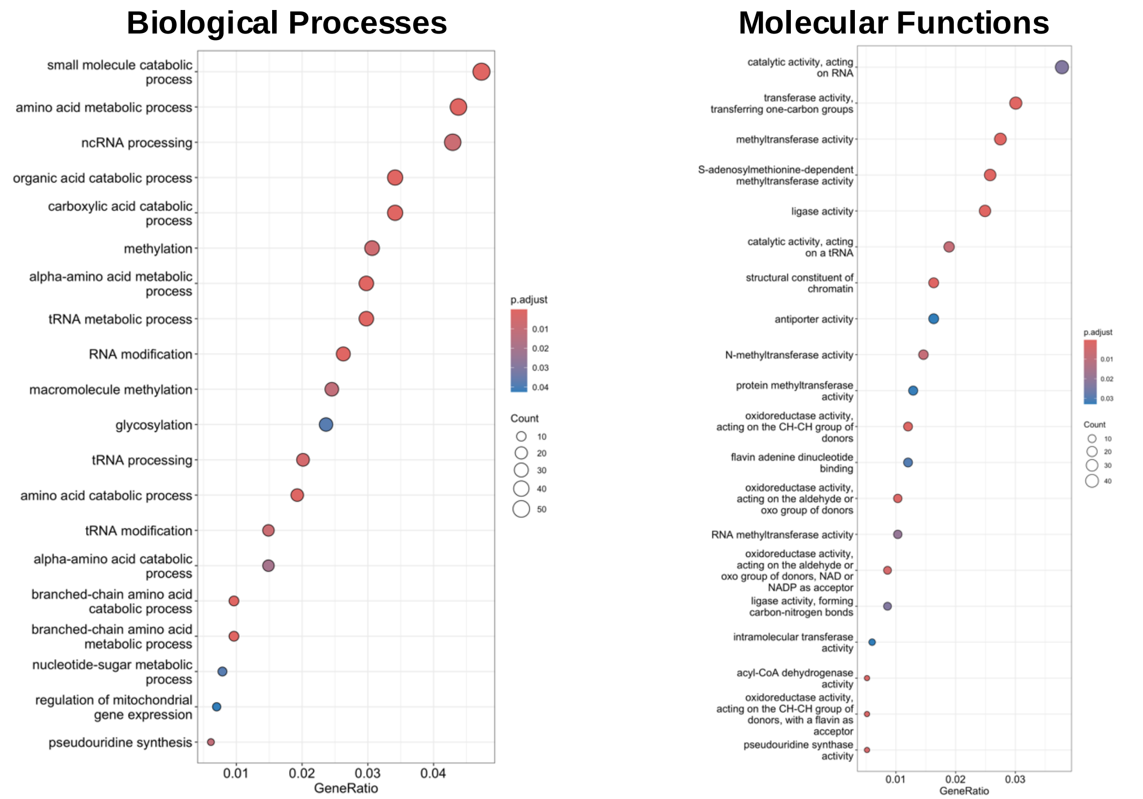

Fig. S4

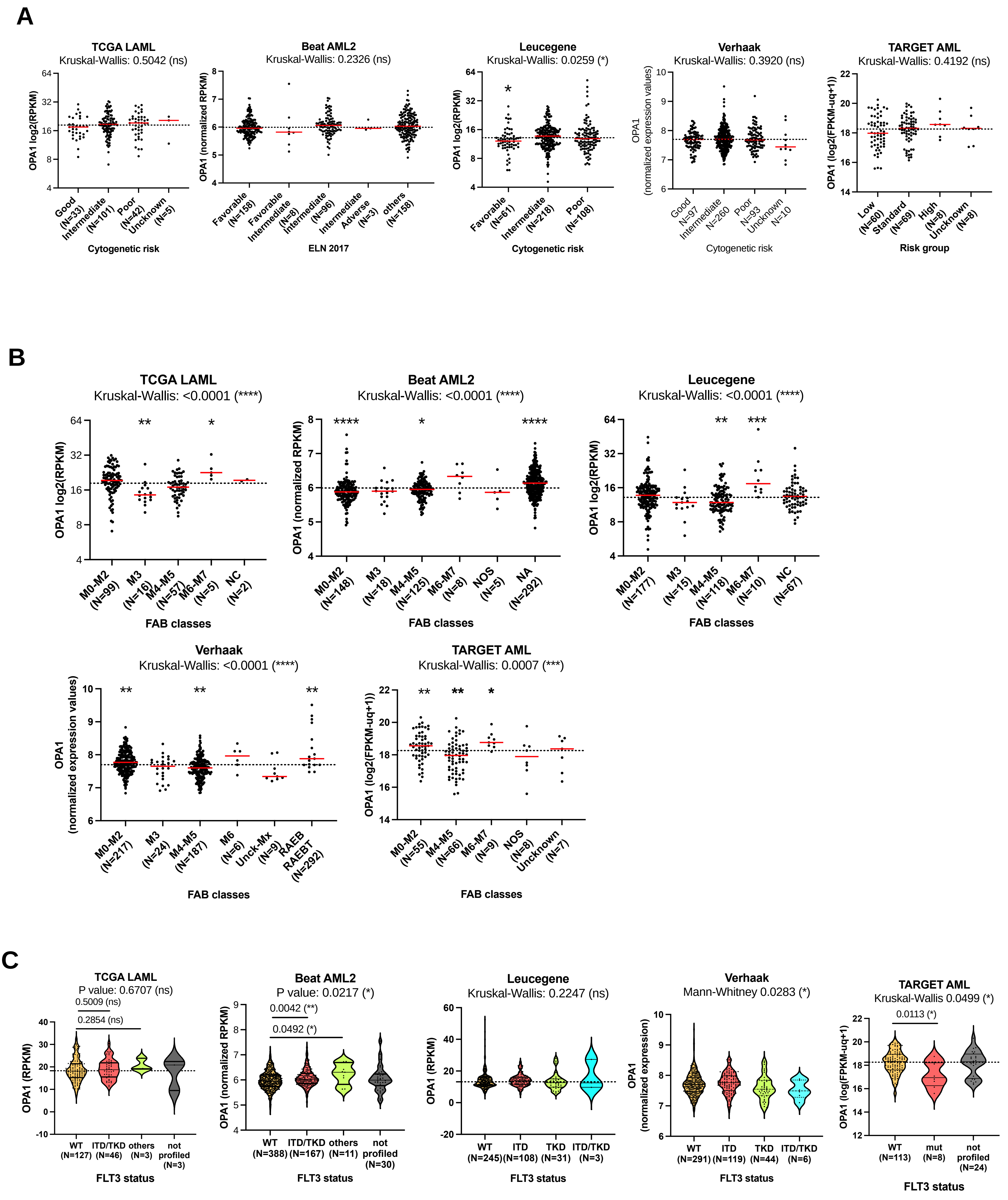
A
TCGA LAML
Kruskal-Wallis: 0.5042 (ns)
OPA1 log2(RPKM)
Good (N=33)
Intermediate (N=101)
Poor (N=42)
Unckown (N=5)
Cytogenetic risk
Beat AML2
Kruskal-Wallis: 0.2326 (ns)
OPA1 (normalized RPKM)
Favorable (N=158)
Favorable Intermediate (N=8)
Intermediate (N=96)
Intermediate Adverse (N=3)
others (N=158)
ELN 2017
Leucegene
Kruskal-Wallis: 0.0259 (*)
OPA1 log2(RPKM)
Favorable (N=61)
Intermediate (N=218)
Poor (N=108)
Cytogenetic risk
Verhaak
Kruskal-Wallis: 0.3920 (ns)
OPA1 (normalized expression values)
Good N=97
Intermediate N=260
Poor N=93
Unknown N=10
Cytogenetic risk
TARGET AML
Kruskal-Wallis: 0.4192 (ns)
OPA1 (log2(FPKM-uq+1))
Low (N=60)
Standard (N=69)
High (N=8)
Uncknown (N=8)
Risk group
B
TCGA LAML
Kruskal-Wallis: <0.0001 (****)
OPA1 log2(RPKM)
M0-M2 (N=99)
M3 (N=16)
M4-M5 (N=57)
M6-M7 (N=5)
NC (N=2)
FAB classes
Beat AML2
Kruskal-Wallis: <0.0001 (****)
OPA1 (normalized RPKM)
M0-M2 (N=148)
M3 (N=18)
M4-M5 (N=125)
M6-M7 (N=8)
NOS (N=5)
NA (N=292)
FAB classes
Leucegene
Kruskal-Wallis: <0.0001 (****)
OPA1 log2(RPKM)
M0-M2 (N=177)
M3 (N=15)
M4-M5 (N=118)
M6-M7 (N=10)
NC (N=67)
FAB classes
Verhaak
Kruskal-Wallis: <0.0001 (****)
OPA1 (normalized expression values)
M0-M2 (N=217)
M3 (N=24)
M4-M5 (N=187)
M6 (N=6)
Unck-Mx (N=9)
RAEB RAEBT (N=292)
FAB classes
TARGET AML
Kruskal-Wallis: 0.0007 (***)
OPA1 (log2(FPKM-uq+1))
M0-M2 (N=55)
M4-M5 (N=66)
M6-M7 (N=9)
NOS (N=8)
Unknown (N=7)
FAB classes
C
TCGA LAML
P value: 0.6707 (ns)
0.5009 (ns)
0.2854 (ns)
OPA1 (RPKM)
WT (N=127)
ITD/TKD (N=46)
others (N=3)
not profiled (N=3)
FLT3 status
Beat AML2
P value: 0.0217 (*)
0.0042 (**)
0.0492 (*)
OPA1 (normalized RPKM)
WT (N=388)
ITD/TKD (N=167)
others (N=11)
not profiled (N=30)
FLT3 status
Leucegene
Kruskal-Wallis: 0.2247 (ns)
OPA1 (RPKM)
WT (N=245)
ITD (N=108)
TKD (N=31)
ITD/TKD (N=3)
FLT3 status
Verhaak
Mann-Whitney 0.0283 (*)
OPA1 (normalized expression)
WT (N=291)
ITD (N=119)
TKD (N=44)
ITD/TKD (N=6)
FLT3 status
TARGET AML
Kruskal-Wallis 0.0499 (*)
0.0113 (*)
OPA1 (log(FPKM-uq+1)
WT (N=113)
mut (N=8)
not profiled (N=24)
FLT3 status

Fig. S5

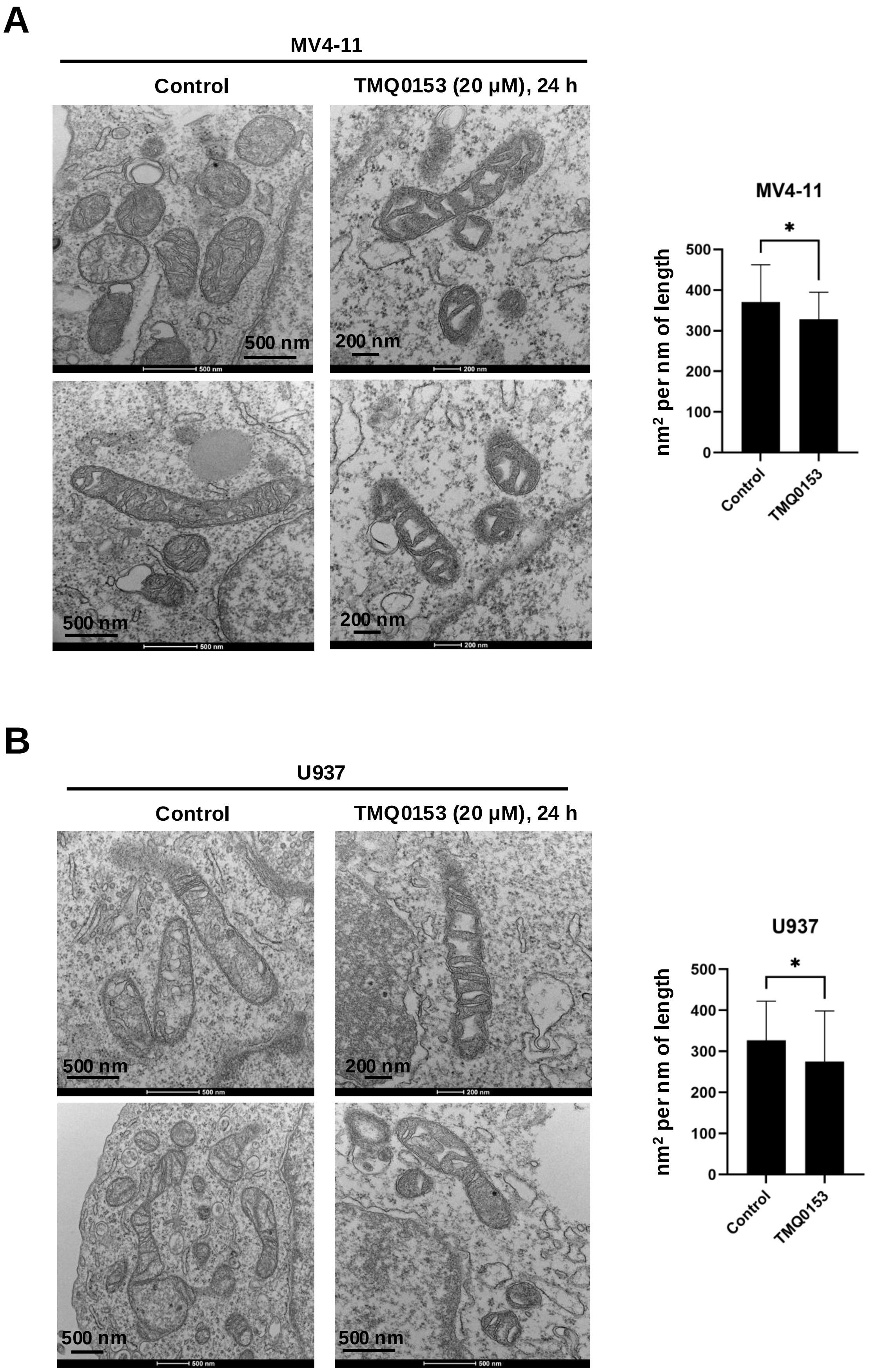
A
MV4-11
Control
TMQ0153 (20 μM), 24 h
500 nm
200 nm
500 nm
200 nm
MV4-11
*
nm2 per nm of length
500
400
300
200
100
0
Control
TMQ0153
B
U937
Control
TMQ0153 (20 μM), 24 h
500 nm
200 nm
500 nm
500 nm
U937
*
nm2 per nm of length
500
400
300
200
100
0
Control
TMQ0153

Fig. S6

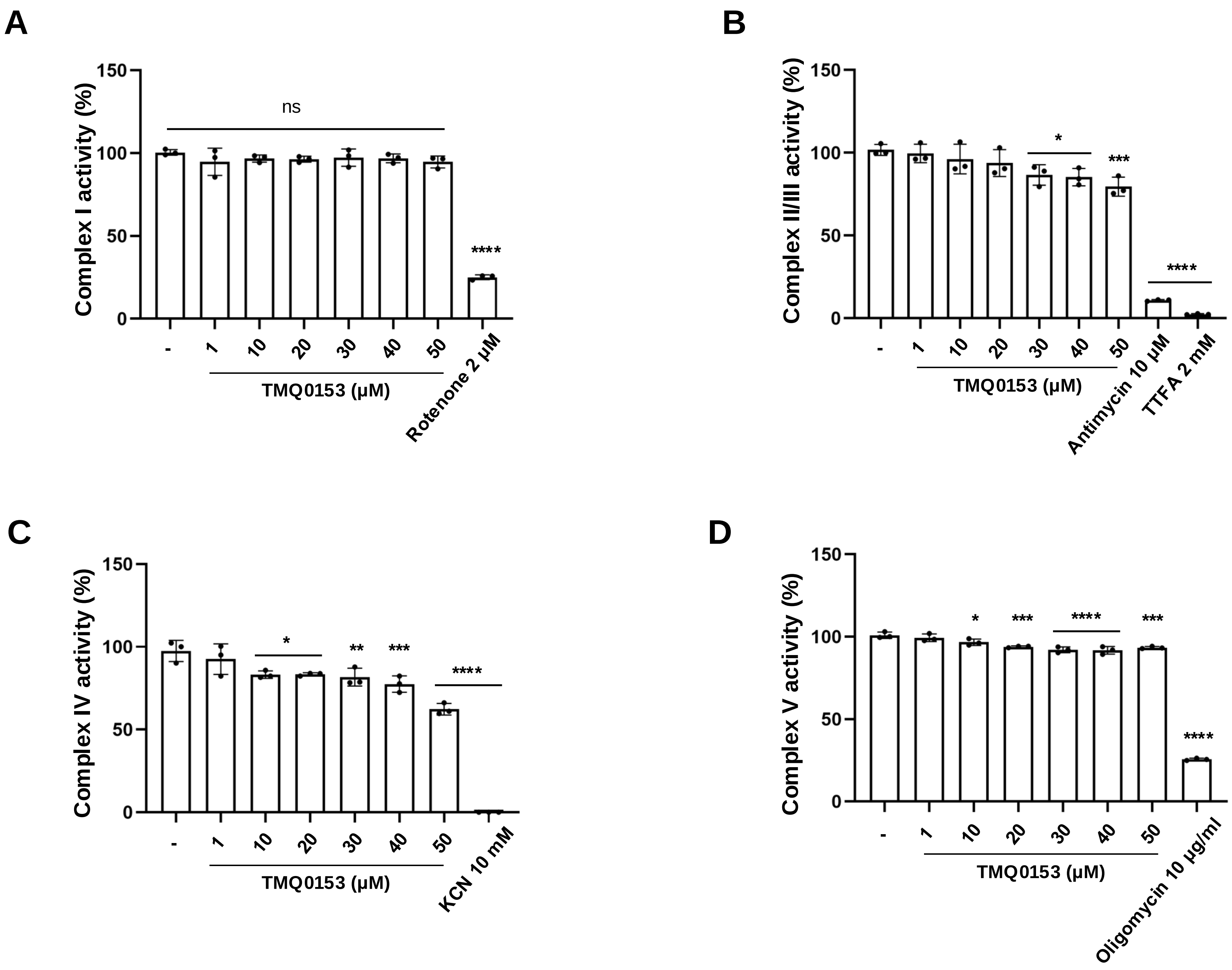
A
Complex I activity (%)
150
100
50
0
ns
****
-
1
10
20
30
40
50
TMQ0153 (μM)
Rotenone 2 μM
B
Complex II/III activity (%)
*
***
****
TMQ0153 (μM)
Antimycin 10 μM
TTFA 2 mM
C
Complex IV activity (%)
*
**
***
****
TMQ0153 (μM)
KCN 10 mM
D
Complex V activity (%)
*
***
****
***
****
TMQ0153 (μM)
Oligomycin 10 μg/ml

Fig. S7

IC50 (μM)_Trypan blue staining				
Cell line	12 h	24 h	48 h	72 h
MV4-11	>50	>50	18.58±3.16	14.49±1.27
U937	>50	37.71±0.31	25.13±0.46	18.56±2.02

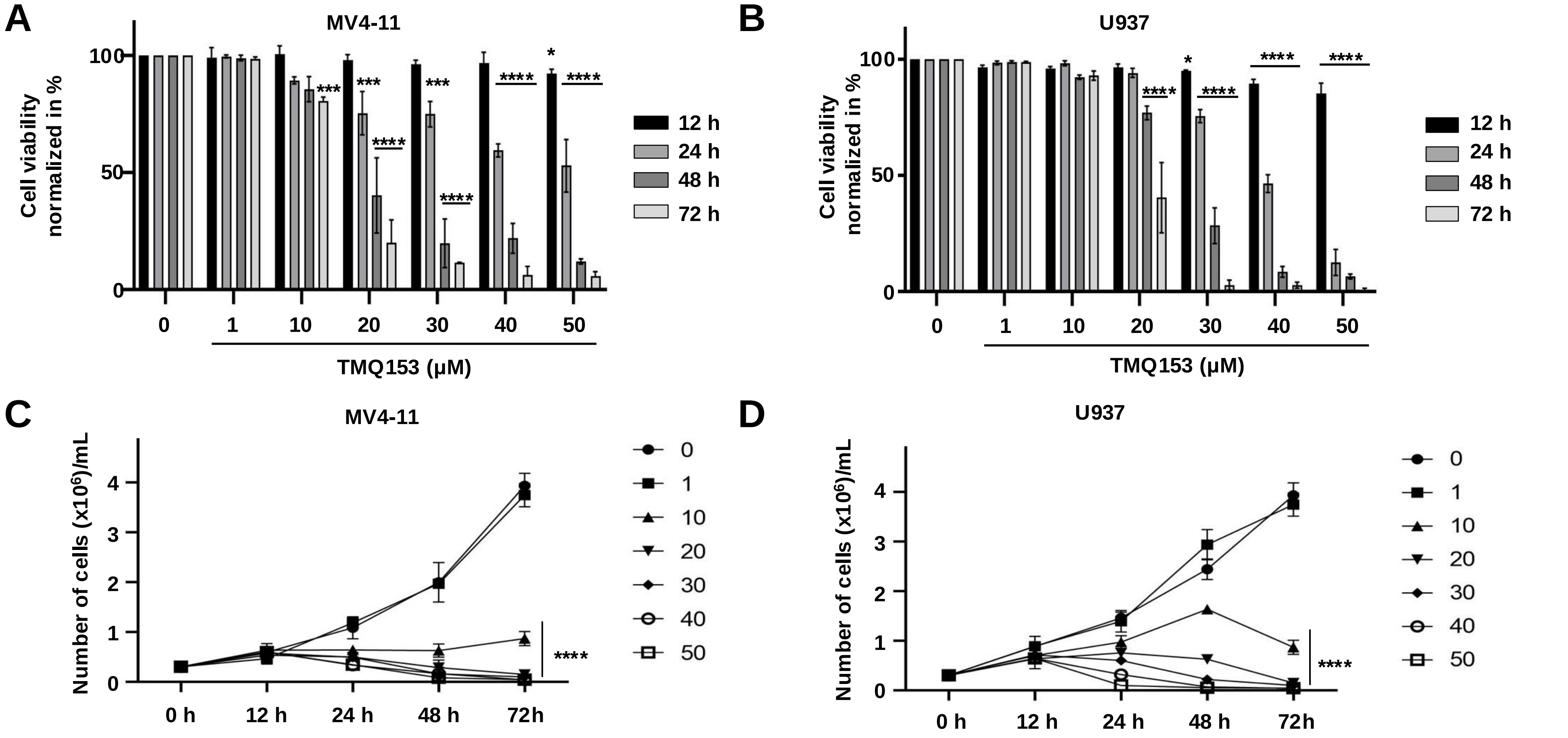

IC50 (μM)_Annexin V/PI staining		
Cell line	6 h	24 h
MV4-11	>50	9.20±4.15
U937	>50	25.68±0.90

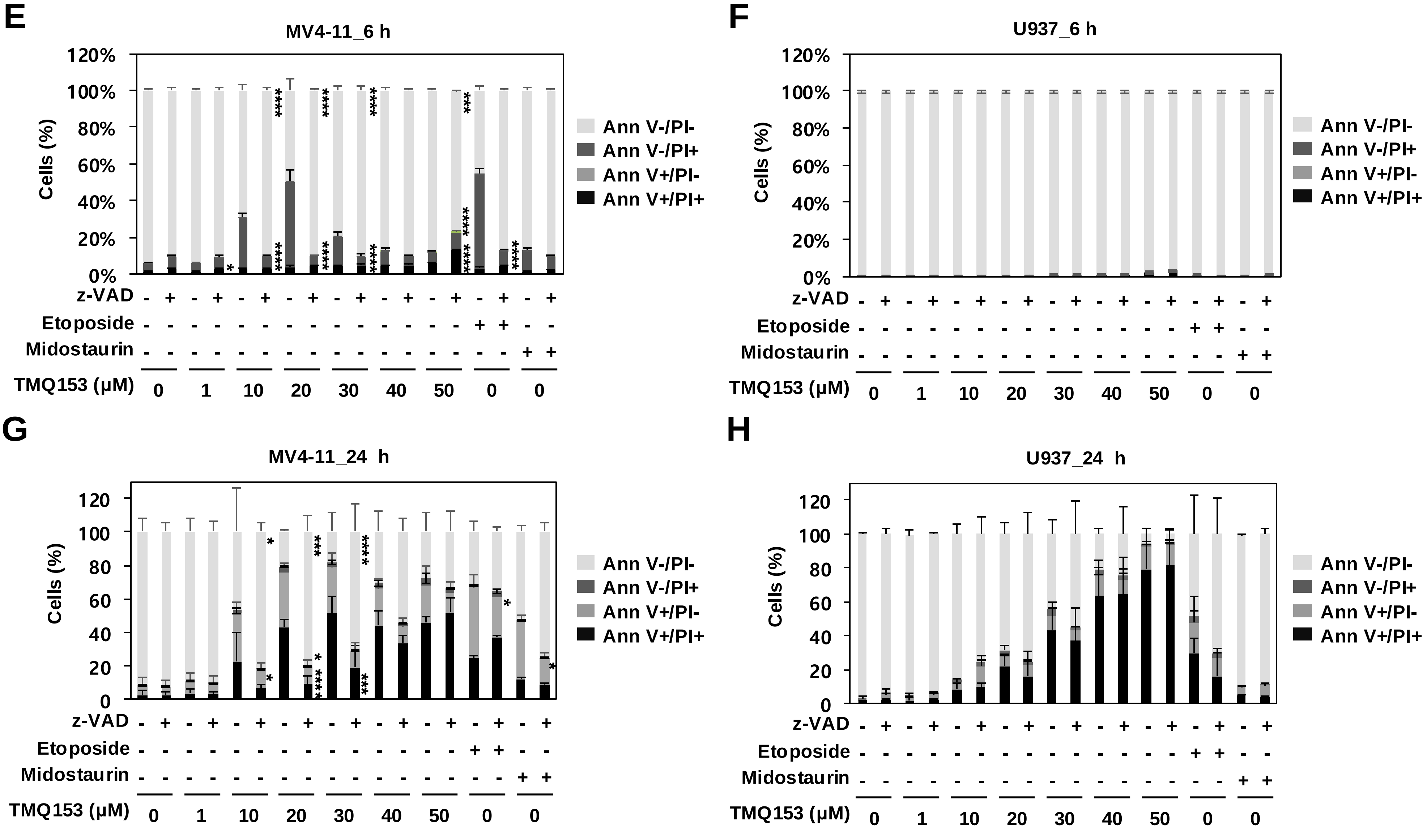

Fig. S8

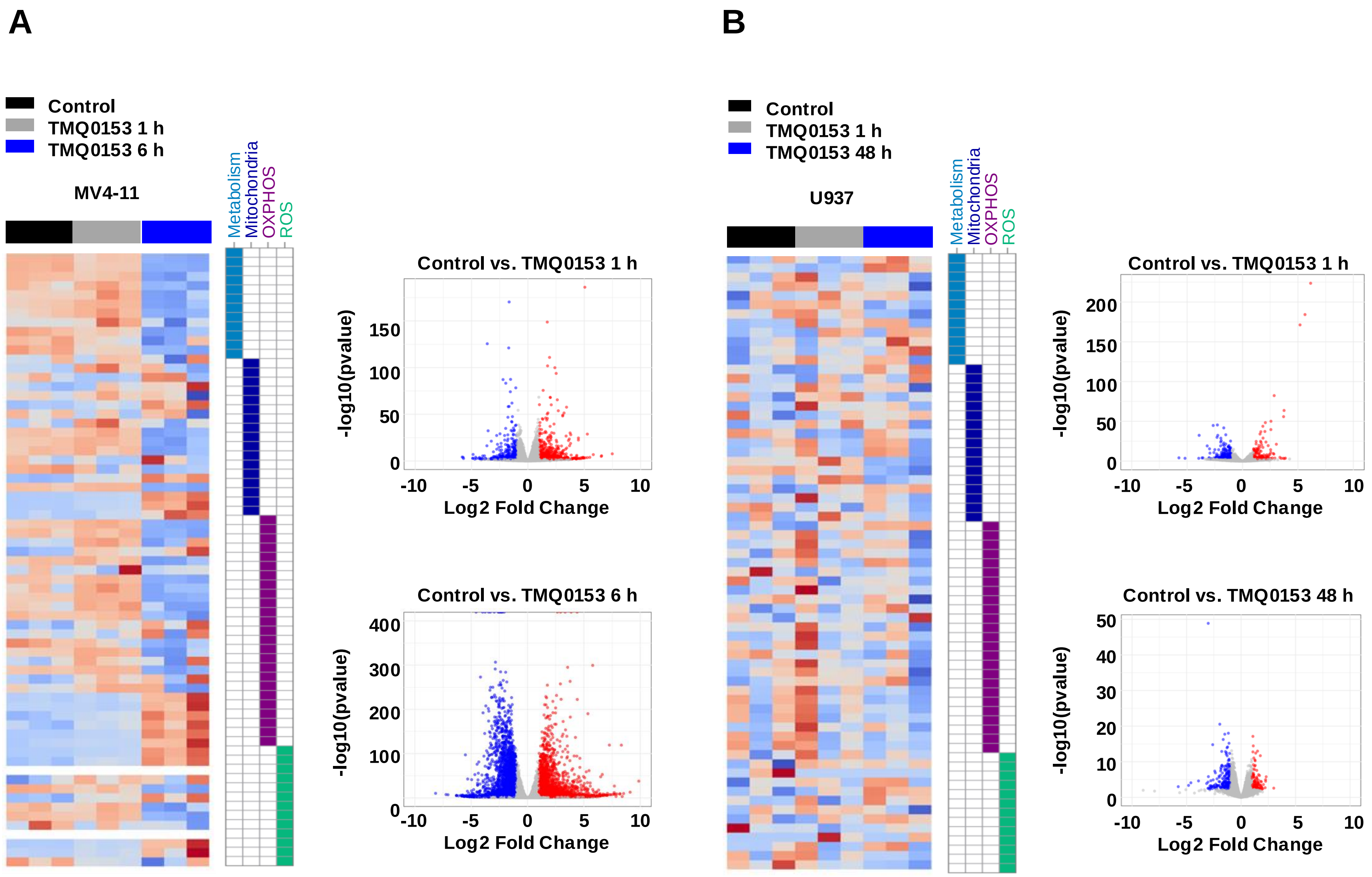
A
Control
TMQ0153 1 h
TMQ0153 6 h
MV4-11
Metabolism
Mitochondria
OXPHOS
ROS
Control vs. TMQ0153 1 h
-log10(pvalue)
Log2 Fold Change
Control vs. TMQ0153 6 h
B
Control
TMQ0153 1 h
TMQ0153 48 h
U937
Control vs. TMQ0153 48 h

Fig. S9

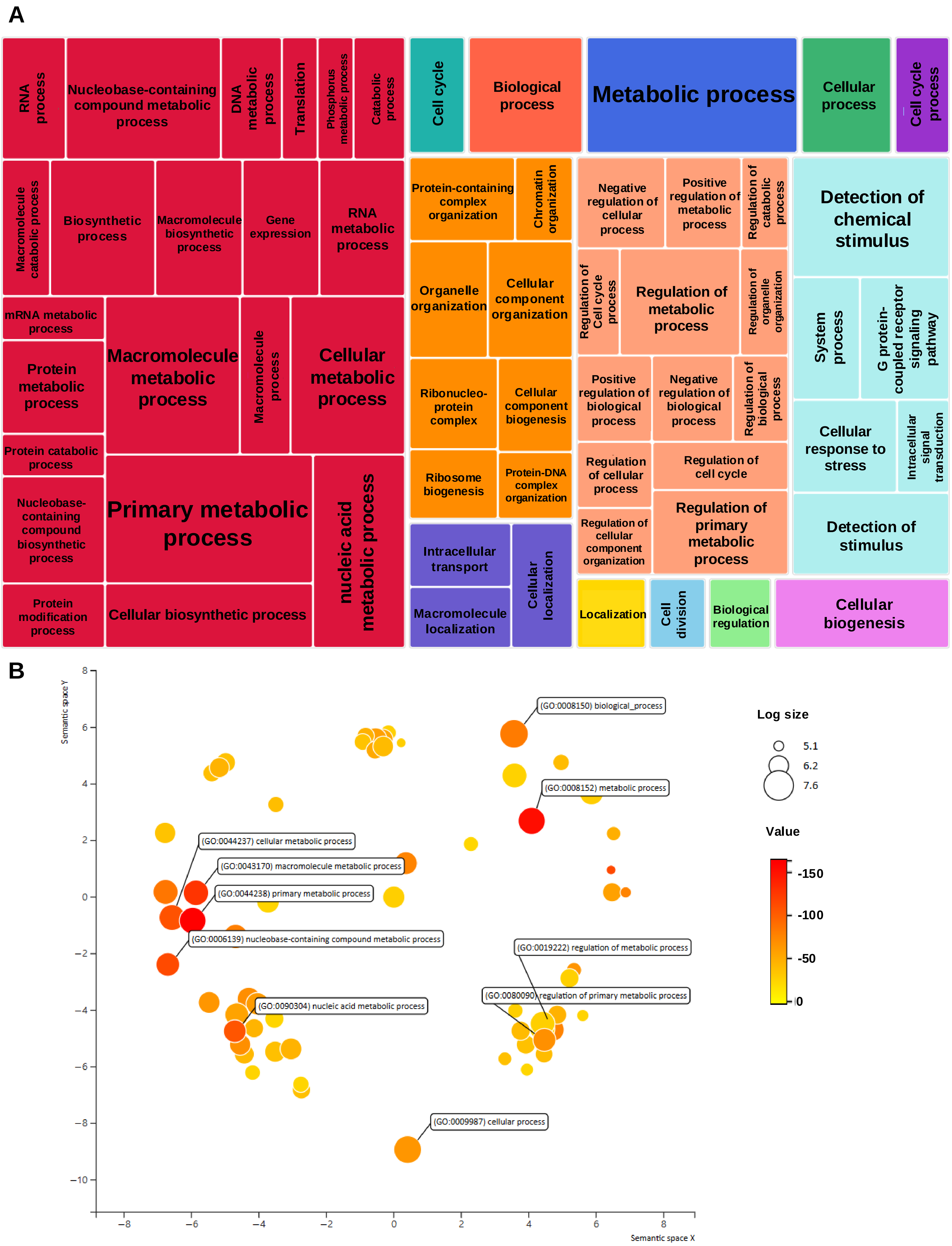
A
RNA process
Nucleobase-containing compound metabolic process
DNA metabolic process
Translation
Phosphorus metabolic process
Catabolic process
Cell cycle
Biological process
Metabolic process
Cellular process
Cell cycle process
Macromolecule catabolic process
Biosynthetic process
Macromolecule biosynthetic process
Gene expression
RNA metabolic process
mRNA metabolic process
Protein metabolic process
Macromolecule metabolic process
Macromolecule process
Cellular metabolic process
Protein catabolic process
Nucleobase-containing compound biosynthetic process
Primary metabolic process
nucleic acid metabolic process
Protein modification process
Cellular biosynthetic process
Protein-containing complex organization
Chromatin organization
Organelle organization
Cellular component organization
Ribonucleoprotein complex
Cellular component biogenesis
Ribosome biogenesis
Protein-DNA complex organization
Intracellular transport
Macromolecule localization
Cellular localization
Negative regulation of cellular process
Positive regulation of metabolic process
Regulation of catabolic process
Regulation of Cell cycle process
Regulation of metabolic process
Regulation of organelle organization
Positive regulation of biological process
Negative regulation of biological process
Regulation of biological process
Regulation of cellular process
Regulation of cell cycle
Regulation of cellular component organization
Regulation of primary metabolic process
Localization
Cell division
Biological regulation
Detection of chemical stimulus
System process
G protein-coupled receptor signaling pathway
Cellular response to stress
Intracellular signal transduction
Detection of stimulus
Cellular biogenesis
B
Semantic space Y
Semantic space X
8
6
4
2
0
−2
−4
−6
−8
−10
−8
−6
−4
−2
0
2
4
6
8
(GO:0008150) biological_process
(GO:0008152) metabolic process
(GO:0044237) cellular metabolic process
(GO:0043170) macromolecule metabolic process
(GO:0044238) primary metabolic process
(GO:0006139) nucleobase-containing compound metabolic process
(GO:0019222) regulation of metabolic process
(GO:0080090) regulation of primary metabolic process
(GO:0090304) nucleic acid metabolic process
(GO:0009987) cellular process
Log size
5.1
6.2
7.6
Value
-150
-100
-50
0

Fig. S9 continued

C

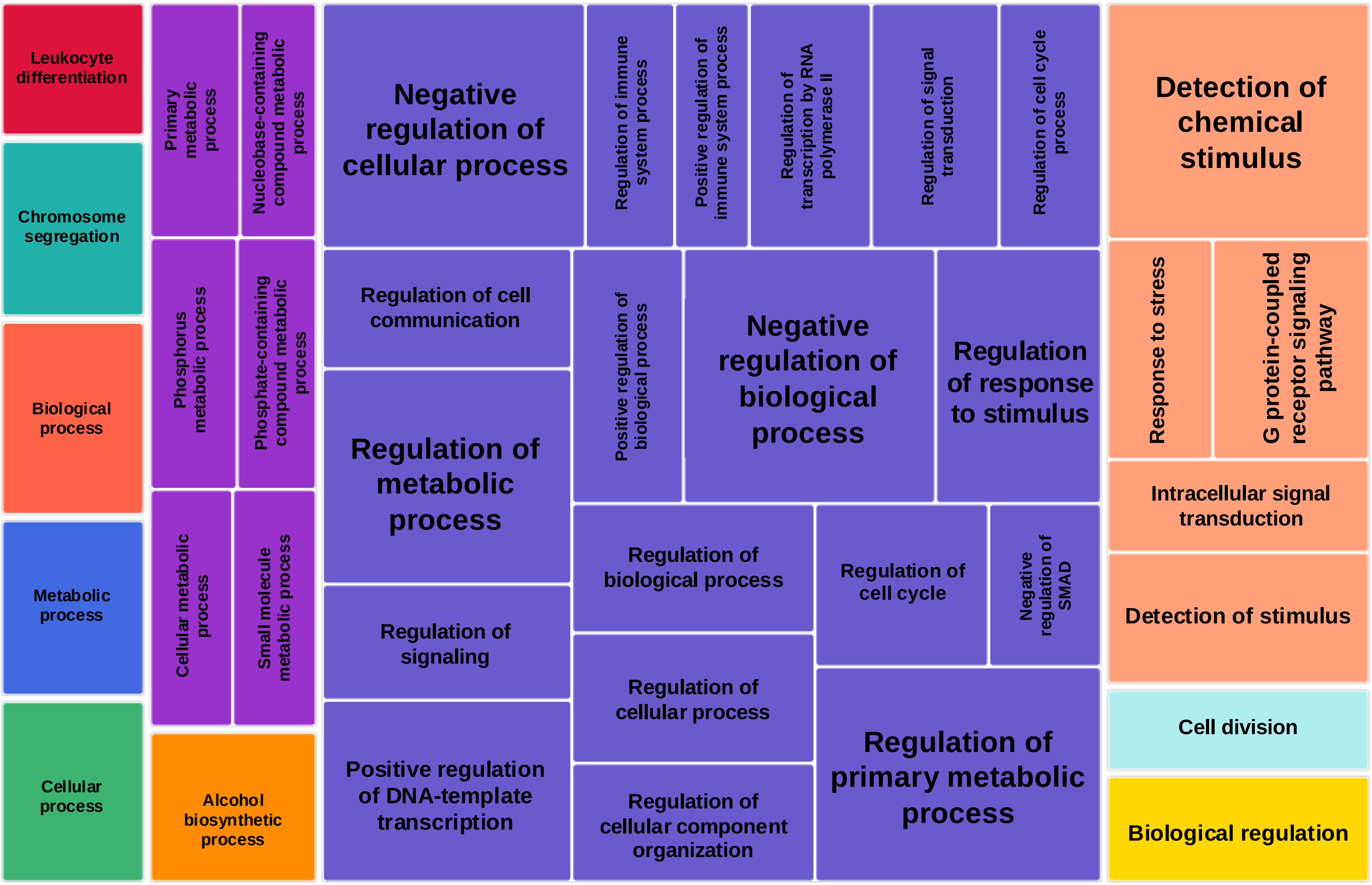

D

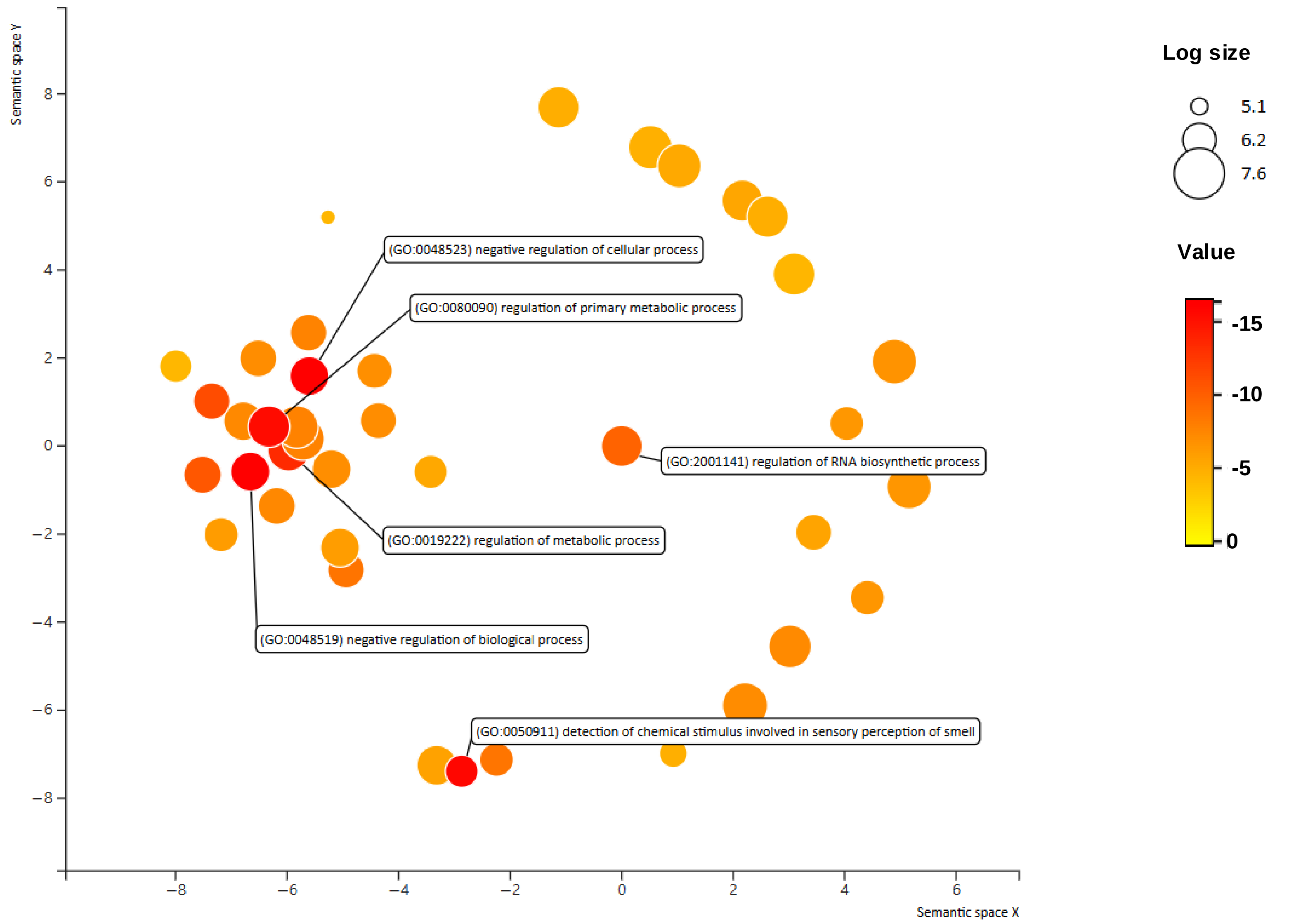

Fig. S10

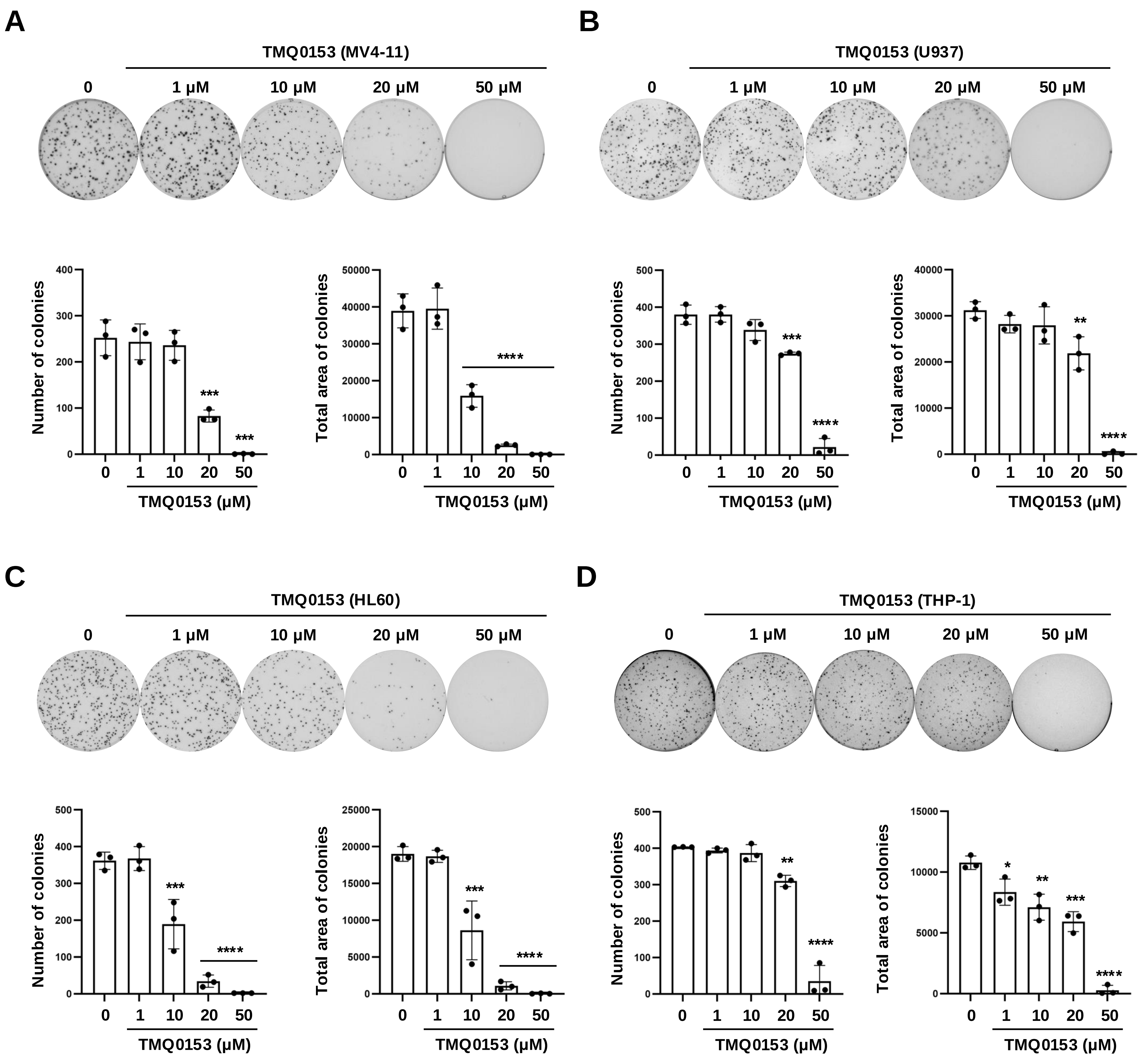

A
TMQ0153 (MV4-11)
0
1 μM
10 μM
20 μM
50 μM
Number of colonies
Total area of colonies
TMQ0153 (μM)
B
TMQ0153 (U937)
C
TMQ0153 (HL60)
D
TMQ0153 (THP-1)

Fig. S11

A

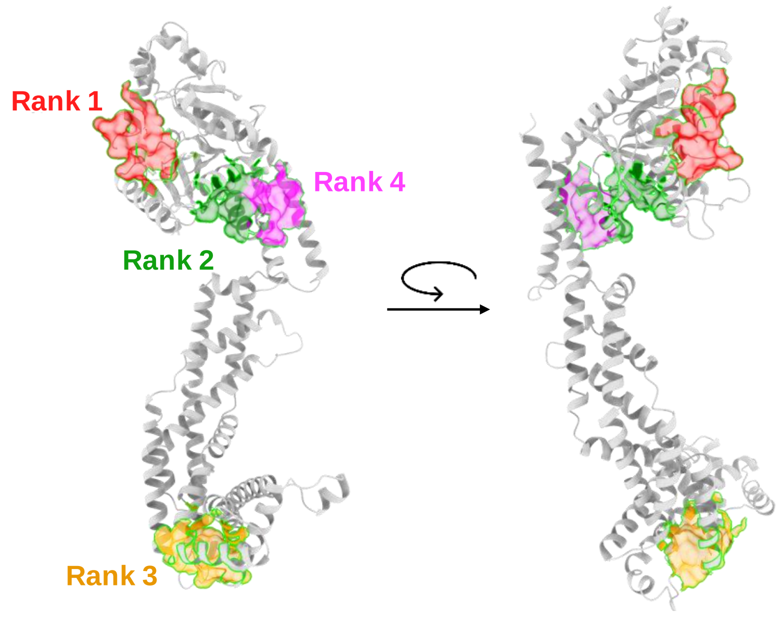

Rank	Probability
1	0.858
2	0.291
3	0.163
4	0.042

B

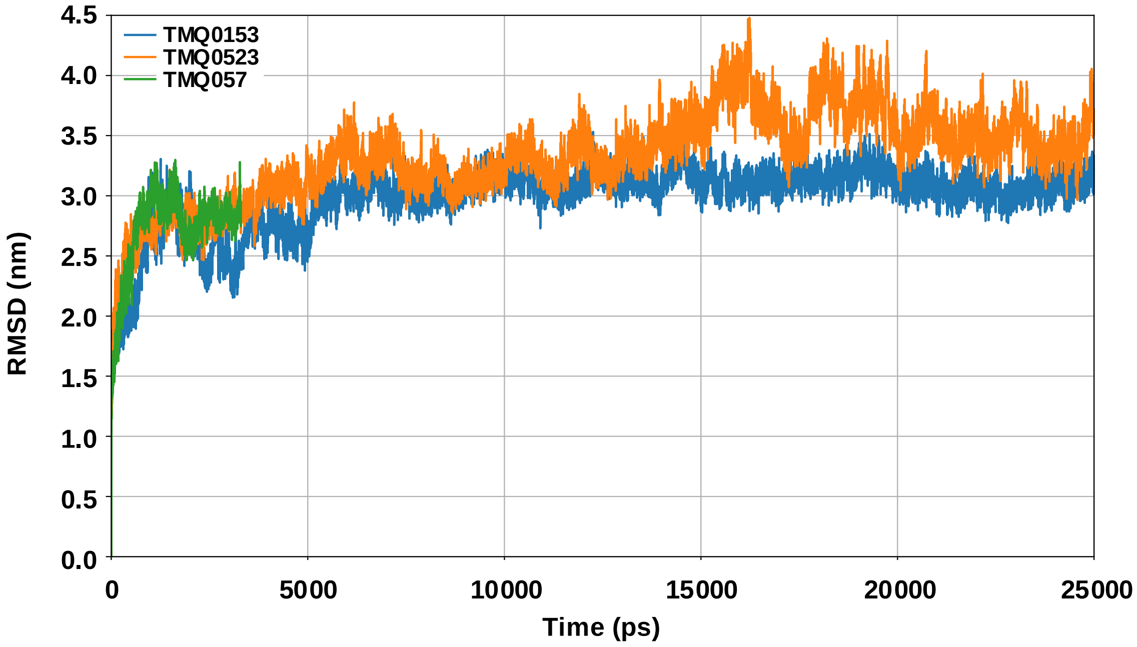

Fig. S12

A

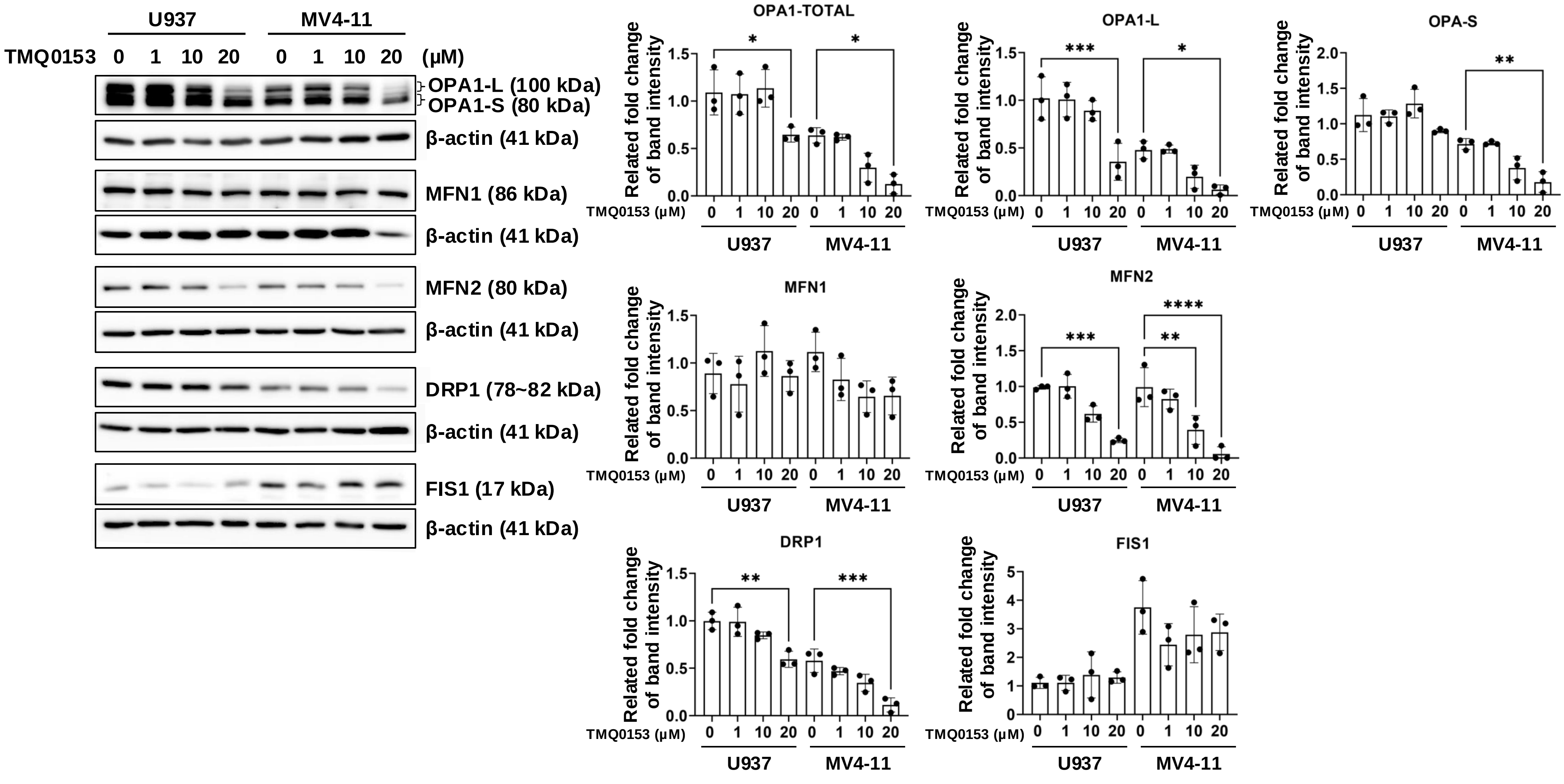
U937
MV4-11
TMQ0153 0 1 10 20 0 1 10 20 (μM)
OPA1-L (100 kDa)
OPA1-S (80 kDa)
β-actin (41 kDa)
MFN1 (86 kDa)
MFN2 (80 kDa)
DRP1 (78~82 kDa)
FIS1 (17 kDa)
OPA1-TOTAL
OPA1-L
OPA-S
MFN1
MFN2
DRP1
FIS1
Related fold change of band intensity
TMQ0153 (μM)

B

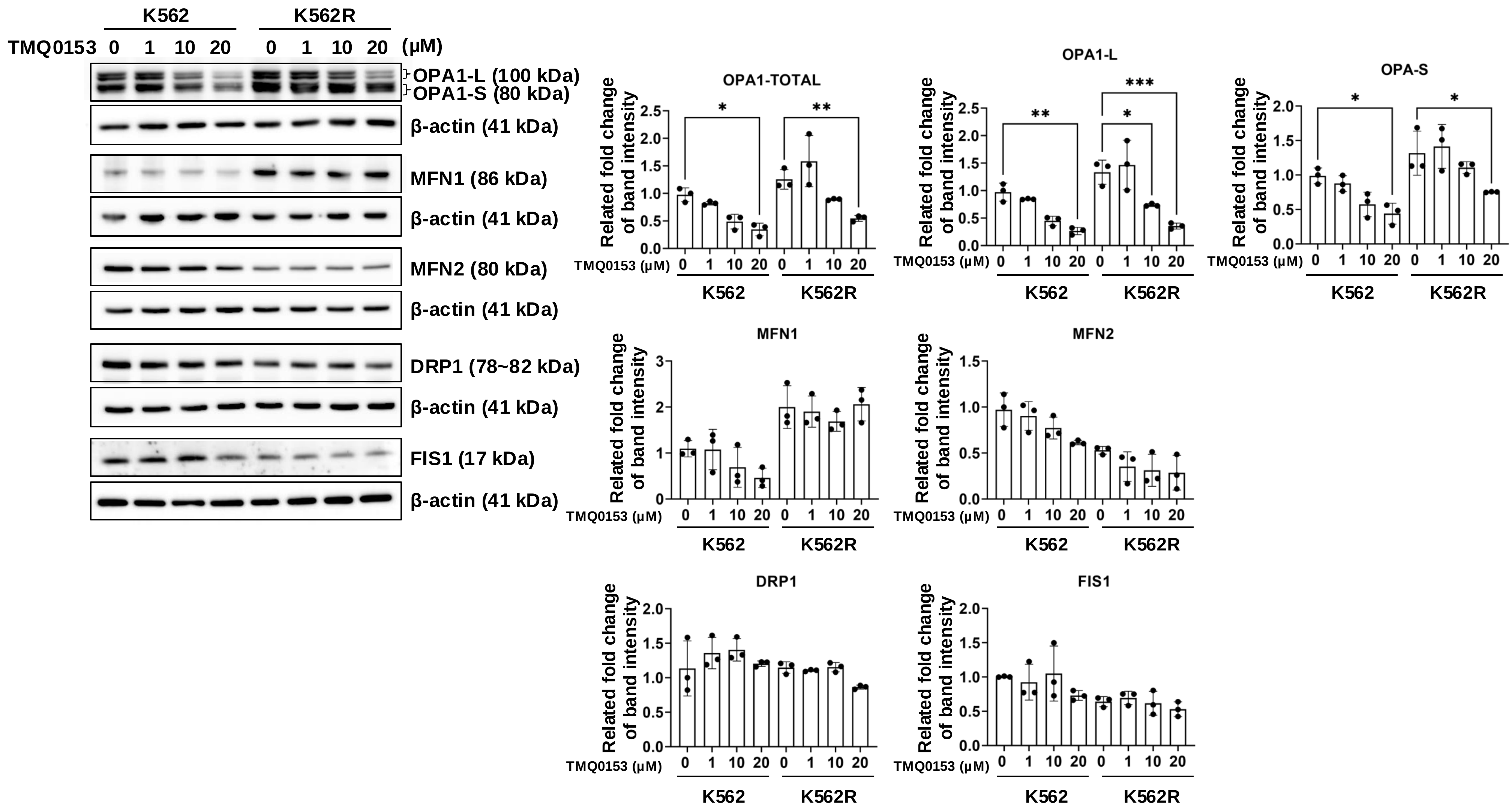
K562
K562R
TMQ0153 0 1 10 20 0 1 10 20 (μM)
OPA1-L (100 kDa)
OPA1-S (80 kDa)
β-actin (41 kDa)
MFN1 (86 kDa)
MFN2 (80 kDa)
DRP1 (78~82 kDa)
FIS1 (17 kDa)
OPA1-TOTAL
OPA1-L
OPA-S
MFN1
MFN2
DRP1
FIS1
Related fold change of band intensity
TMQ0153 (μM)

Fig. S13

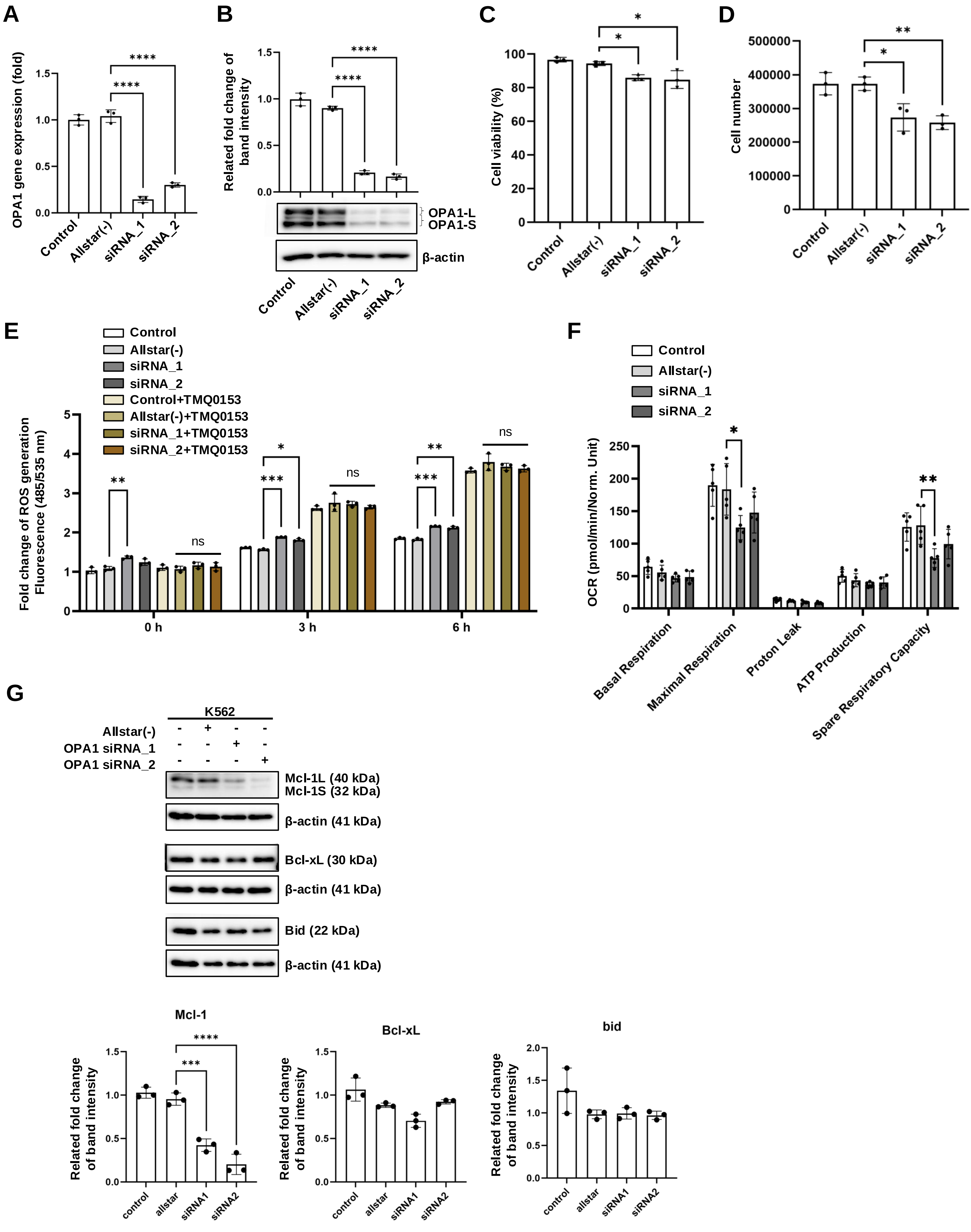

A
OPA1 gene expression (fold)
Control
Allstar(-)
siRNA_1
siRNA_2
****
B
Related fold change of band intensity
OPA1-L
OPA1-S
β-actin
C
Cell viability (%)
D
Cell number
E
Control
Allstar(-)
siRNA_1
siRNA_2
Control+TMQ0153
Allstar(-)+TMQ0153
siRNA_1+TMQ0153
siRNA_2+TMQ0153
Fold change of ROS generation Fluorescence (485/535 nm)
0 h
3 h
6 h
ns
F
OCR (pmol/min/Norm. Unit)
Basal Respiration
Maximal Respiration
Proton Leak
ATP Production
Spare Respiratory Capacity
G
K562
Allstar(-)
OPA1 siRNA_1
OPA1 siRNA_2
Mcl-1L (40 kDa)
Mcl-1S (32 kDa)
β-actin (41 kDa)
Bcl-xL (30 kDa)
Bid (22 kDa)
Mcl-1
Bcl-xL
bid
Related fold change of band intensity
control
allstar
siRNA1
siRNA2

Fig. S14

A

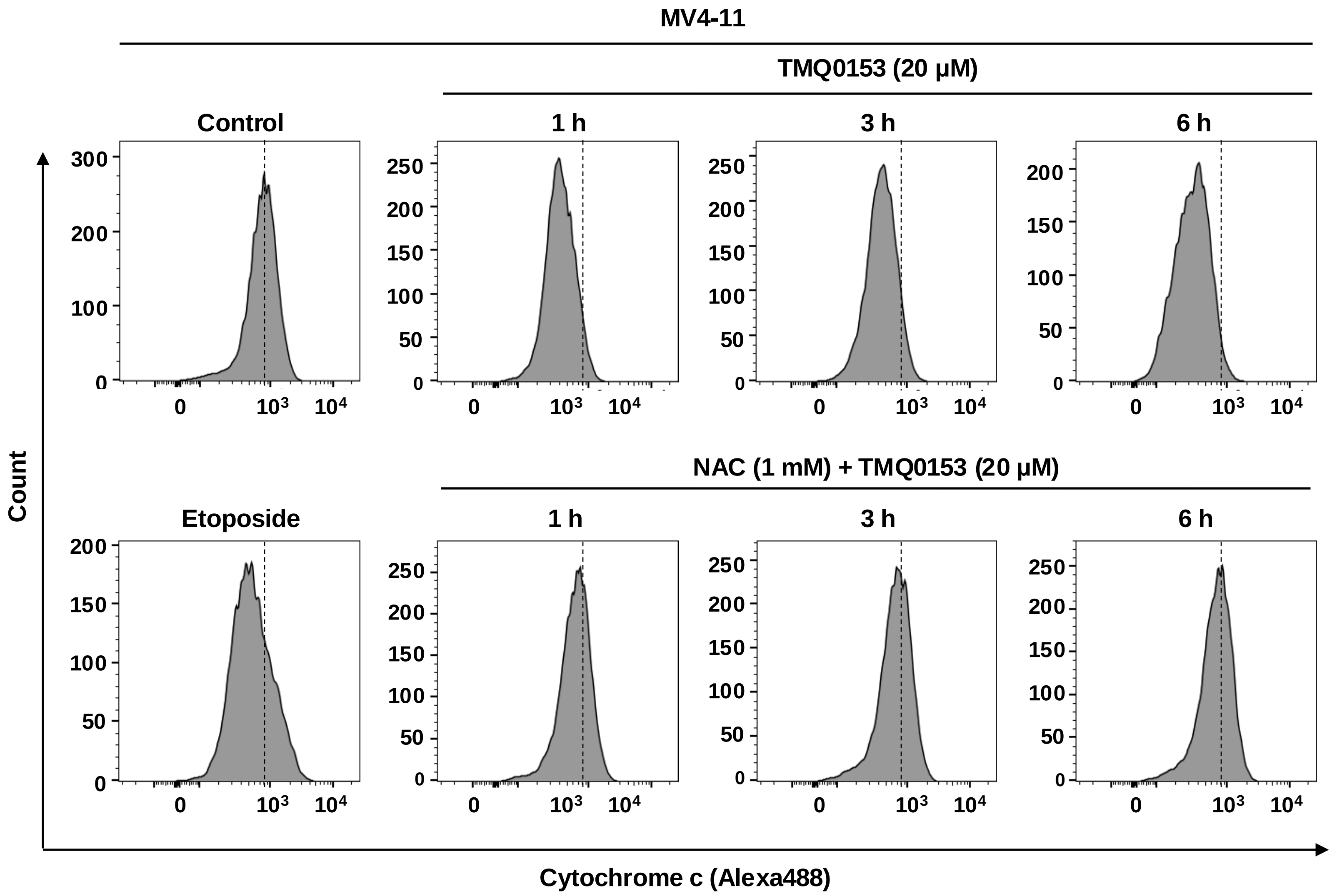

MV4-11
TMQ0153 (20 μM)
Control
1 h
3 h
6 h
NAC (1 mM) + TMQ0153 (20 μM)
Etoposide
Count
Cytochrome c (Alexa488)

B

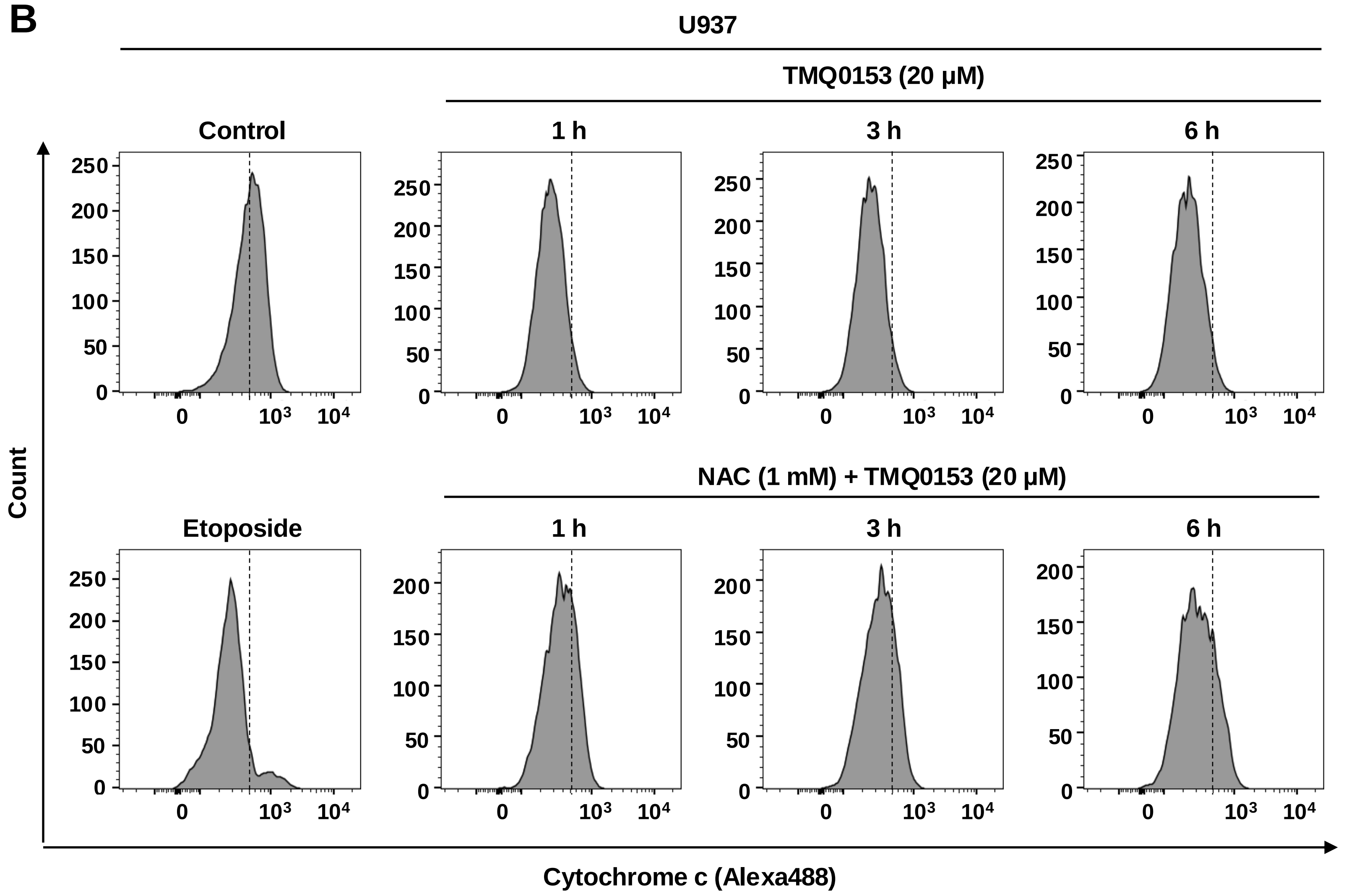

U937
TMQ0153 (20 μM)
Control
1 h
3 h
6 h
NAC (1 mM) + TMQ0153 (20 μM)
Etoposide
Count
Cytochrome c (Alexa488)

Fig. S15

A

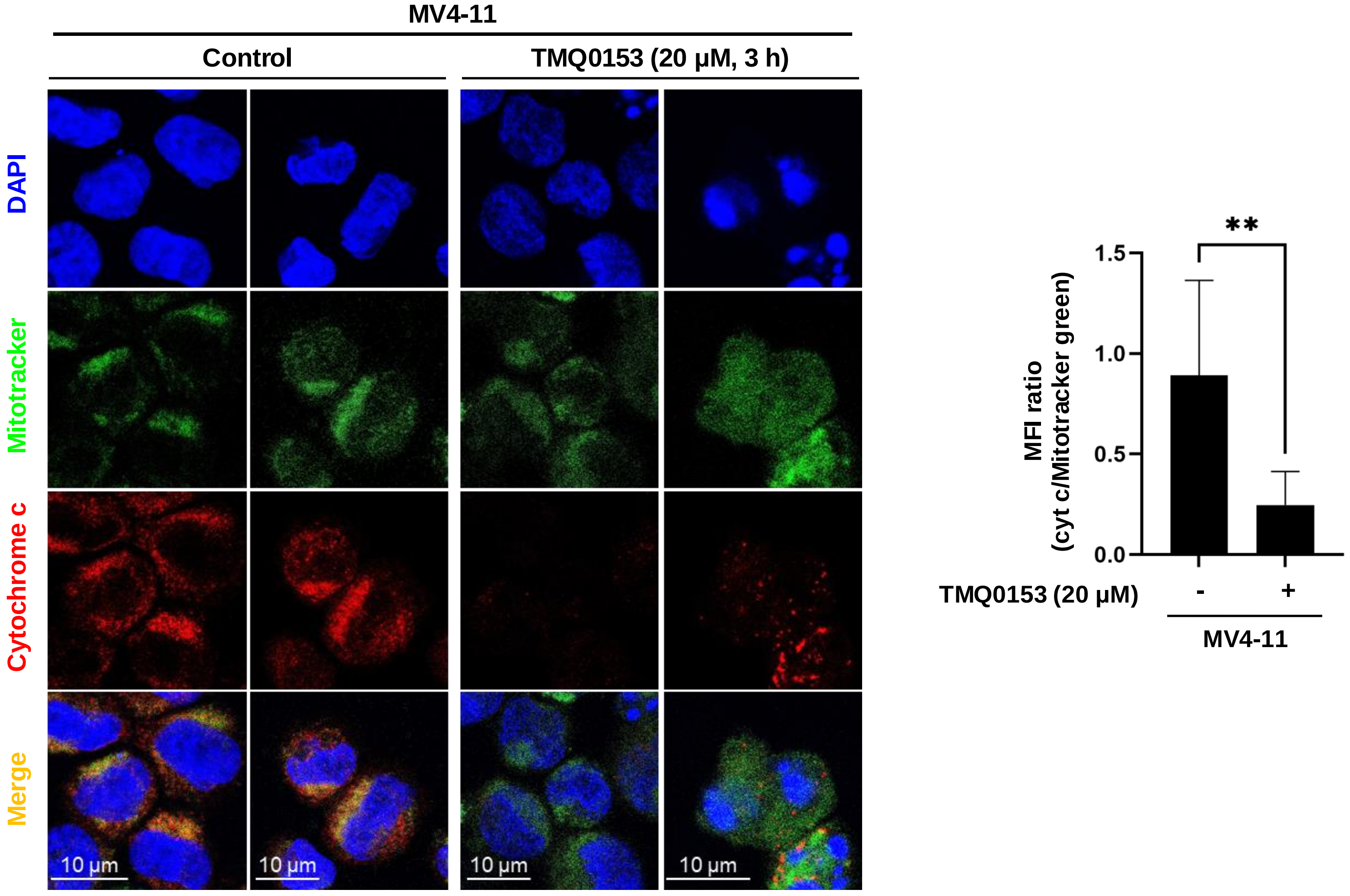

MV4-11
Control
TMQ0153 (20 µM, 3 h)
DAPI
Mitotracker
Cytochrome c
Merge
10 µm
10 µm
10 µm
10 µm
MFI ratio
(cyt c/Mitotracker green)
1.5
1.0
0.5
0.0
**
TMQ0153 (20 µM)
-
+
MV4-11

B

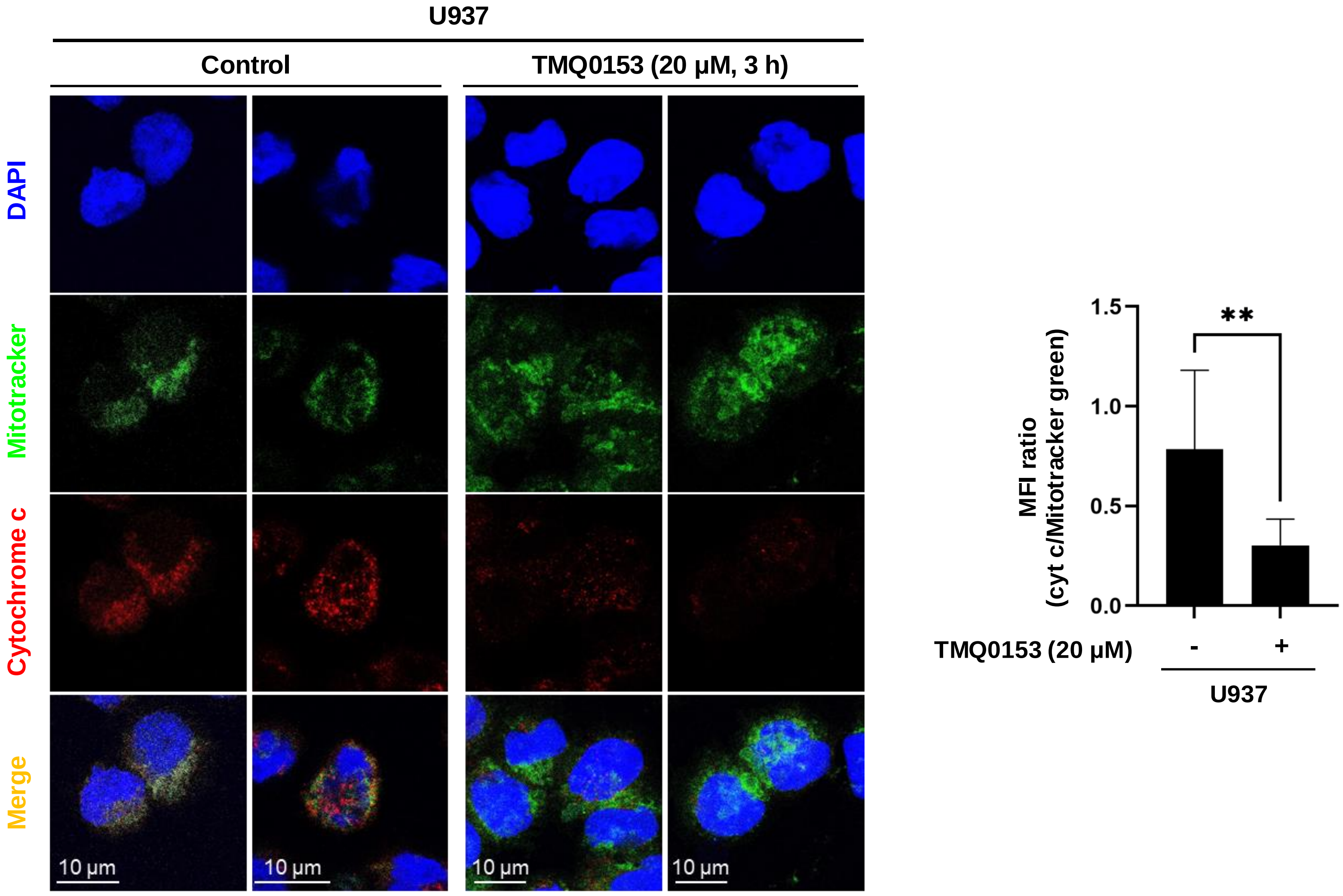

U937
Control
TMQ0153 (20 µM, 3 h)
DAPI
Mitotracker
Cytochrome c
Merge
10 µm
10 µm
10 µm
10 µm
MFI ratio
(cyt c/Mitotracker green)
1.5
1.0
0.5
0.0
**
TMQ0153 (20 µM)
-
+
U937

Fig. S16

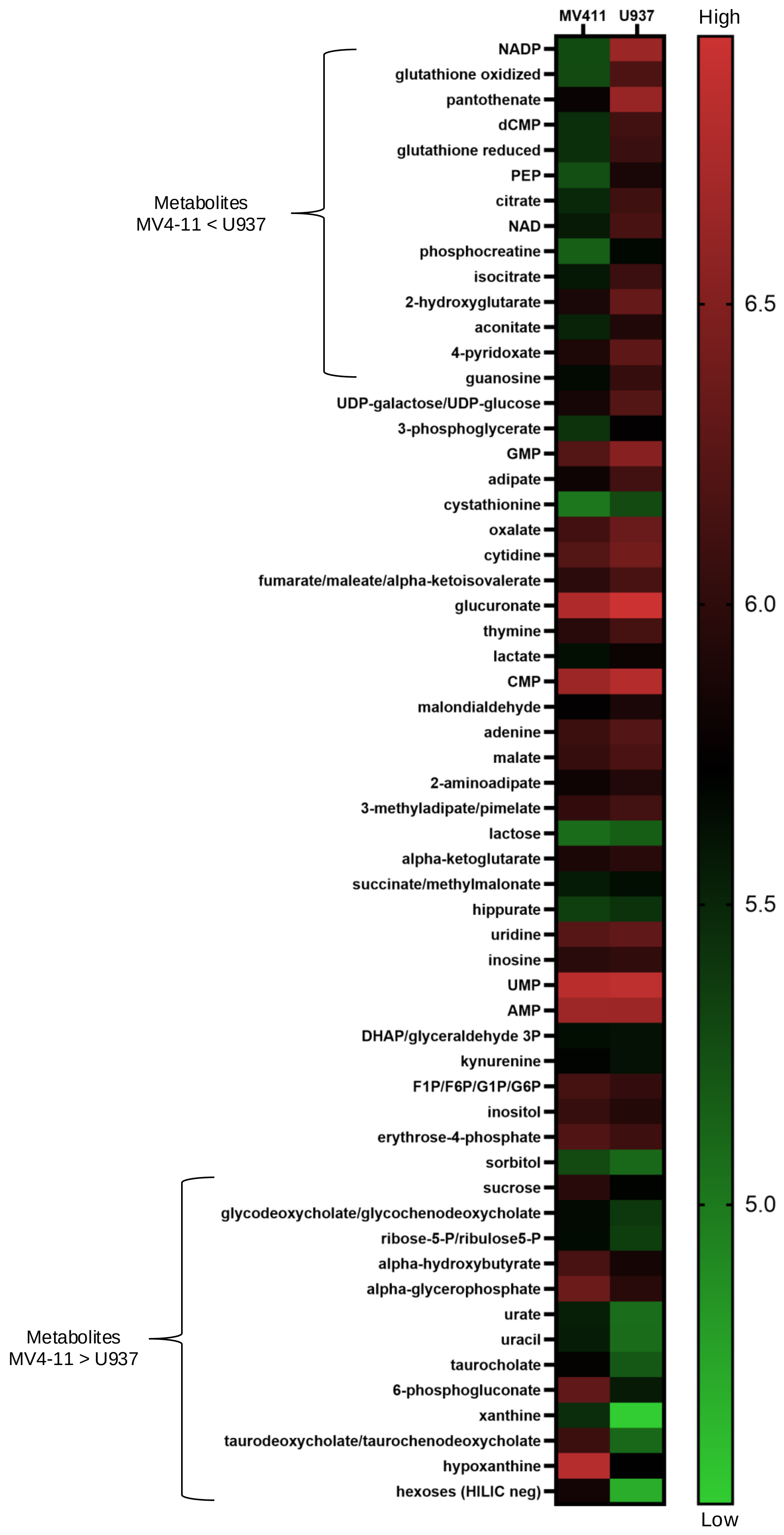
MV411
U937
High
Low
6.5
6.0
5.5
5.0
Metabolites
MV4-11 < U937
Metabolites
MV4-11 > U937
NADP
glutathione oxidized
pantothenate
dCMP
glutathione reduced
PEP
citrate
NAD
phosphocreatine
isocitrate
2-hydroxyglutarate
aconitate
4-pyridoxate
guanosine
UDP-galactose/UDP-glucose
3-phosphoglycerate
GMP
adipate
cystathionine
oxalate
cytidine
fumarate/maleate/alpha-ketoisovalerate
glucuronate
thymine
lactate
CMP
malondialdehyde
adenine
malate
2-aminoadipate
3-methyladipate/pimelate
lactose
alpha-ketoglutarate
succinate/methylmalonate
hippurate
uridine
inosine
UMP
AMP
DHAP/glyceraldehyde 3P
kynurenine
F1P/F6P/G1P/G6P
inositol
erythrose-4-phosphate
sorbitol
sucrose
glycodeoxycholate/glycochenodeoxycholate
ribose-5-P/ribulose5-P
alpha-hydroxybutyrate
alpha-glycerophosphate
urate
uracil
taurocholate
6-phosphogluconate
xanthine
taurodeoxycholate/taurochenodeoxycholate
hypoxanthine
hexoses (HILIC neg)

Fig. S17

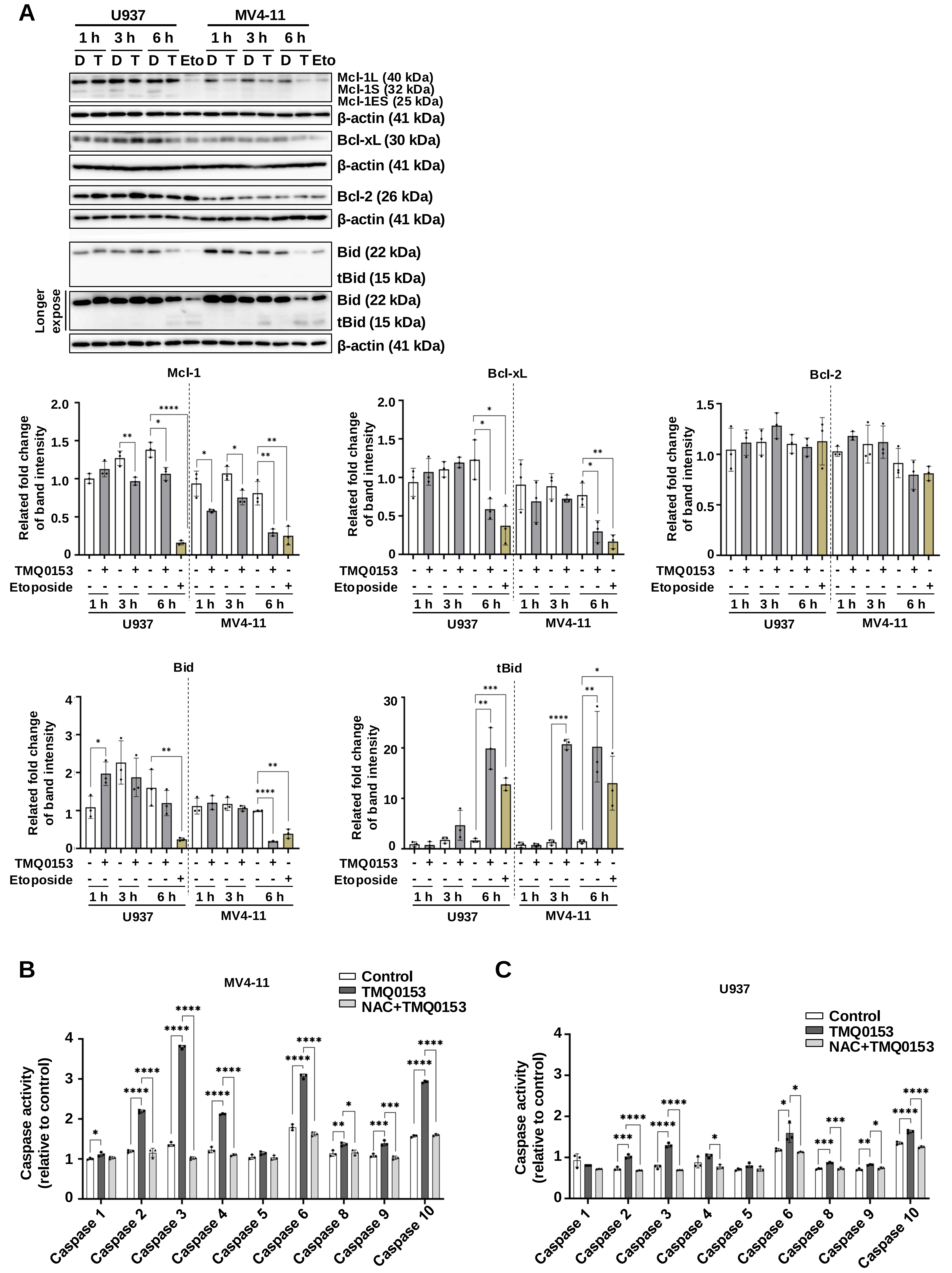
A
U937
MV4-11
1 h
3 h
6 h
D
T
Eto
Mcl-1L (40 kDa)
Mcl-1S (32 kDa)
Mcl-1ES (25 kDa)
β-actin (41 kDa)
Bcl-xL (30 kDa)
Bcl-2 (26 kDa)
Bid (22 kDa)
tBid (15 kDa)
Longer expose
Mcl-1
Bcl-xL
Bcl-2
Bid
tBid
Related fold change of band intensity
TMQ0153
Etoposide
B
MV4-11
Control
TMQ0153
NAC+TMQ0153
Caspase activity (relative to control)
Caspase 1
Caspase 2
Caspase 3
Caspase 4
Caspase 5
Caspase 6
Caspase 8
Caspase 9
Caspase 10
C
U937

Fig. S17 continued

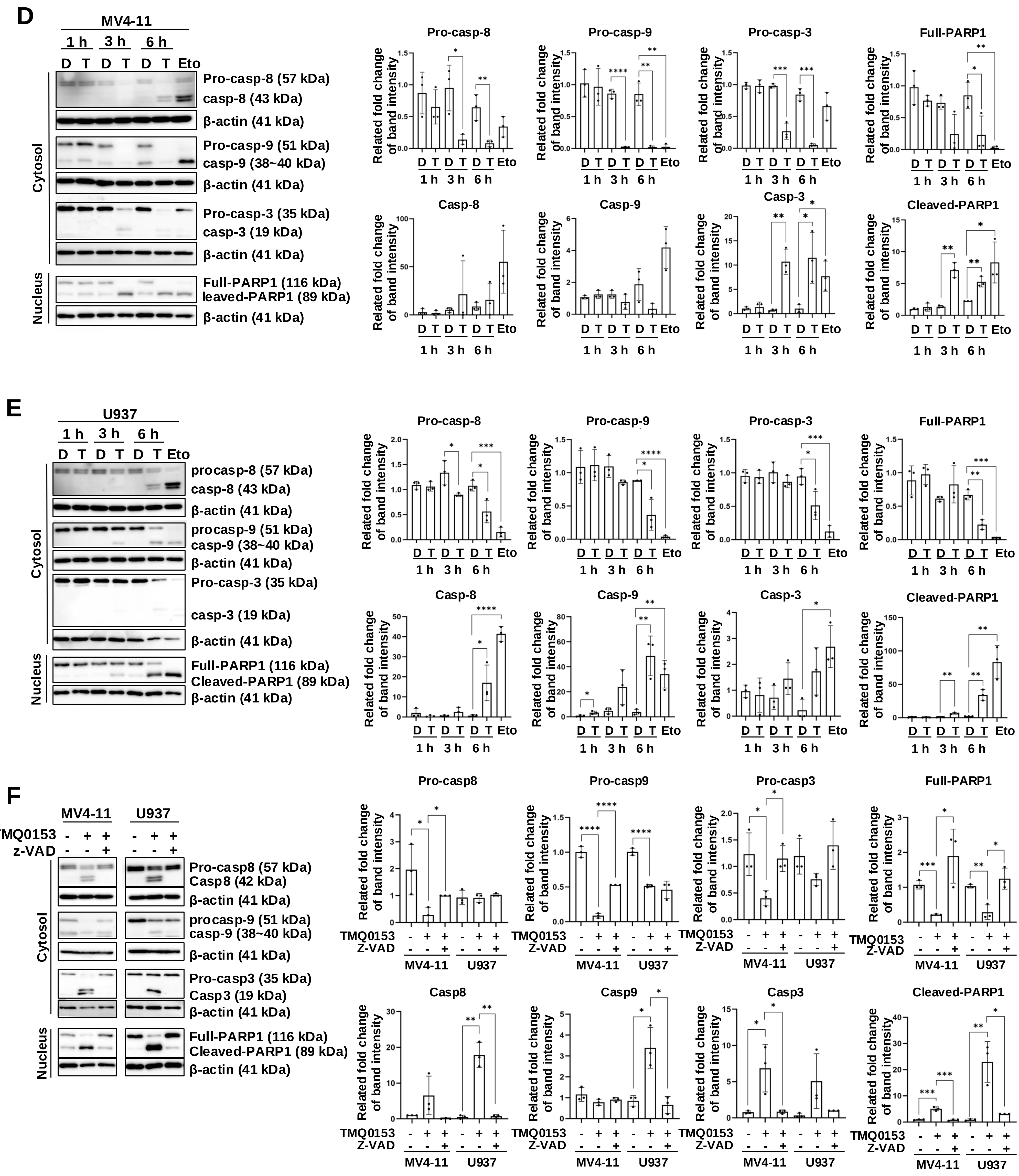

Fig. S18

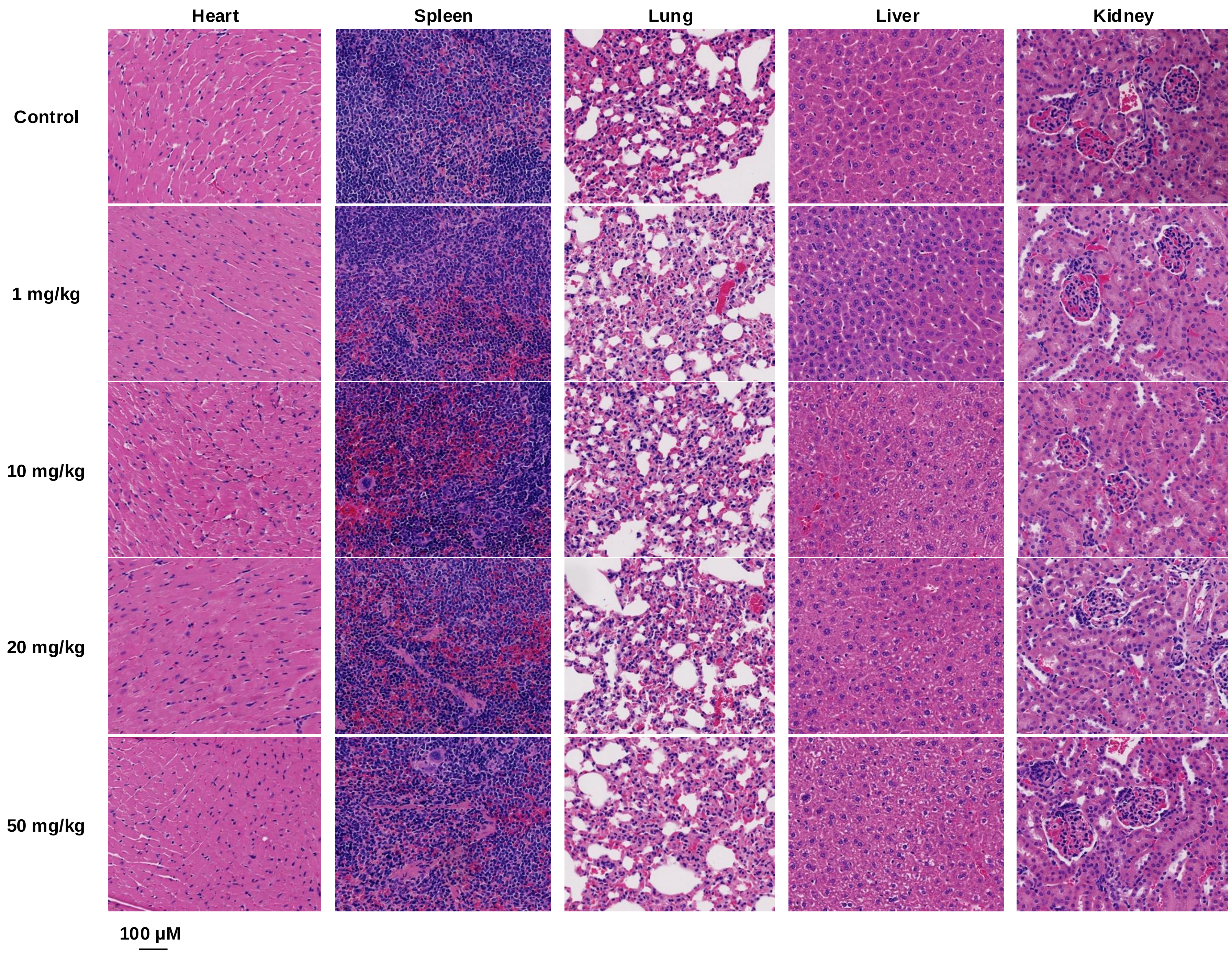
Heart
Spleen
Lung
Liver
Kidney
Control
1 mg/kg
10 mg/kg
20 mg/kg
50 mg/kg
100 μM

Fig. S19

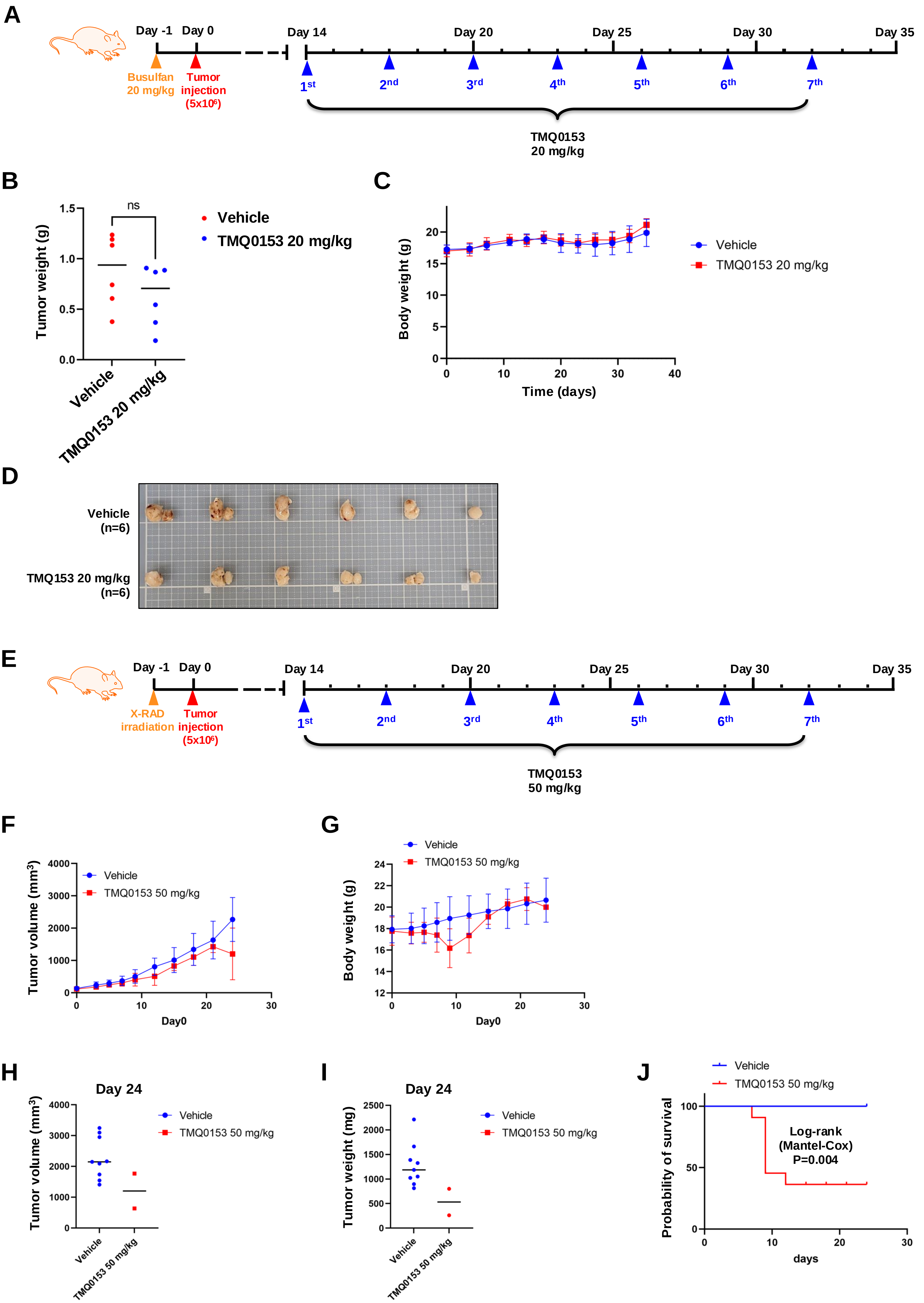
A
Day -1
Day 0
Day 14
Day 20
Day 25
Day 30
Day 35
Busulfan 20 mg/kg
Tumor injection (5x10⁶)
1st
2nd
3rd
4th
5th
6th
7th
TMQ0153 20 mg/kg
B
ns
Tumor weight (g)
1.5
1.0
0.5
0.0
Vehicle
TMQ0153 20 mg/kg
C
Body weight (g)
Time (days)
0
5
10
15
20
30
40
Vehicle
TMQ0153 20 mg/kg
D
Vehicle (n=6)
TMQ153 20 mg/kg (n=6)
E
Day -1
Day 0
Day 14
Day 20
Day 25
Day 30
Day 35
X-RAD irradiation
Tumor injection (5x10⁶)
1st
2nd
3rd
4th
5th
6th
7th
TMQ0153 50 mg/kg
F
Tumor volume (mm³)
4000
3000
2000
1000
0
10
20
30
Day0
Vehicle
TMQ0153 50 mg/kg
G
Body weight (g)
24
22
20
18
16
14
12
0
10
20
30
Day0
Vehicle
TMQ0153 50 mg/kg
H
Day 24
Tumor volume (mm³)
4000
3000
2000
1000
0
Vehicle
TMQ0153 50 mg/kg
I
Day 24
Tumor weight (mg)
2500
2000
1500
1000
500
0
Vehicle
TMQ0153 50 mg/kg
J
Vehicle
TMQ0153 50 mg/kg
Probability of survival
100
50
0
Log-rank (Mantel-Cox) P=0.004
0
10
20
30
days

Fig. S20

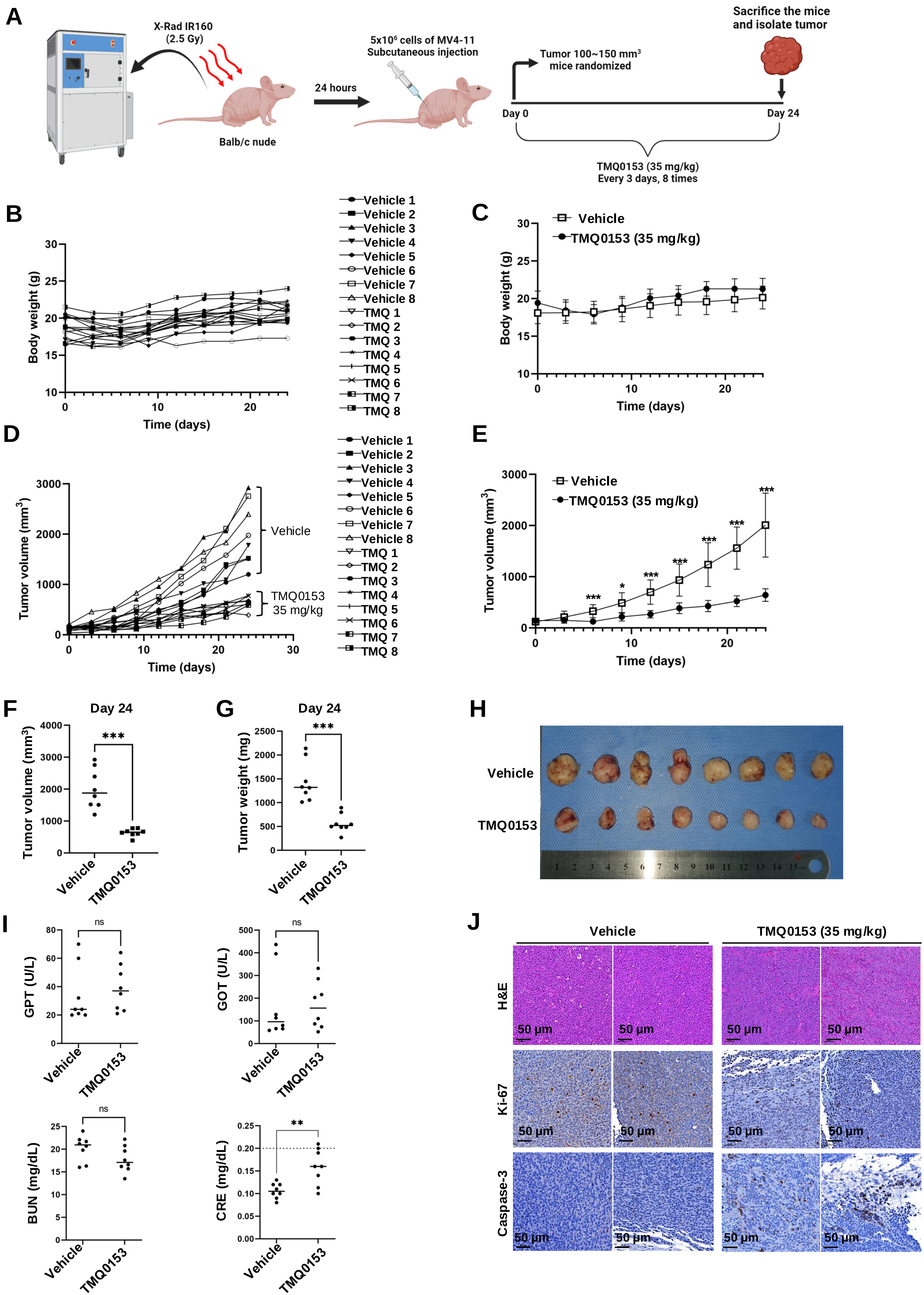

A
X-Rad IR160
(2.5 Gy)
Balb/c nude
24 hours
5x10⁶ cells of MV4-11
Subcutaneous injection
Tumor 100~150 mm³
mice randomized
Sacrifice the mice
and isolate tumor
Day 0
Day 24
TMQ0153 (35 mg/kg)
Every 3 days, 8 times
B
Body weight (g)
Time (days)
Vehicle 1
Vehicle 2
Vehicle 3
Vehicle 4
Vehicle 5
Vehicle 6
Vehicle 7
Vehicle 8
TMQ 1
TMQ 2
TMQ 3
TMQ 4
TMQ 5
TMQ 6
TMQ 7
TMQ 8
C
Vehicle
TMQ0153 (35 mg/kg)
Body weight (g)
Time (days)
D
Tumor volume (mm³)
Time (days)
Vehicle
TMQ0153
35 mg/kg
E
Vehicle
TMQ0153 (35 mg/kg)
Tumor volume (mm³)
Time (days)
F
Day 24
Tumor volume (mm³)
Vehicle
TMQ0153
G
Day 24
Tumor weight (mg)
Vehicle
TMQ0153
H
Vehicle
TMQ0153
I
GPT (U/L)
GOT (U/L)
BUN (mg/dL)
CRE (mg/dL)
ns
**
Vehicle
TMQ0153
J
Vehicle
TMQ0153 (35 mg/kg)
H&E
Ki-67
Caspase-3
50 μm

Fig. S21

IC50 (MV4-11)	
Time	Gilteritinib (nM)
24 h	>100
48 h	>100
72 h	15.44±2.01

IC50 (MOLM-14-luc)		
Time	TMQ0153 (μM)	Gilteritinib (nM)
24 h	>50	>100
48 h	19.81±0.95	67.36±17.10
72 h	17.89±1.05	23.14±2.48

A

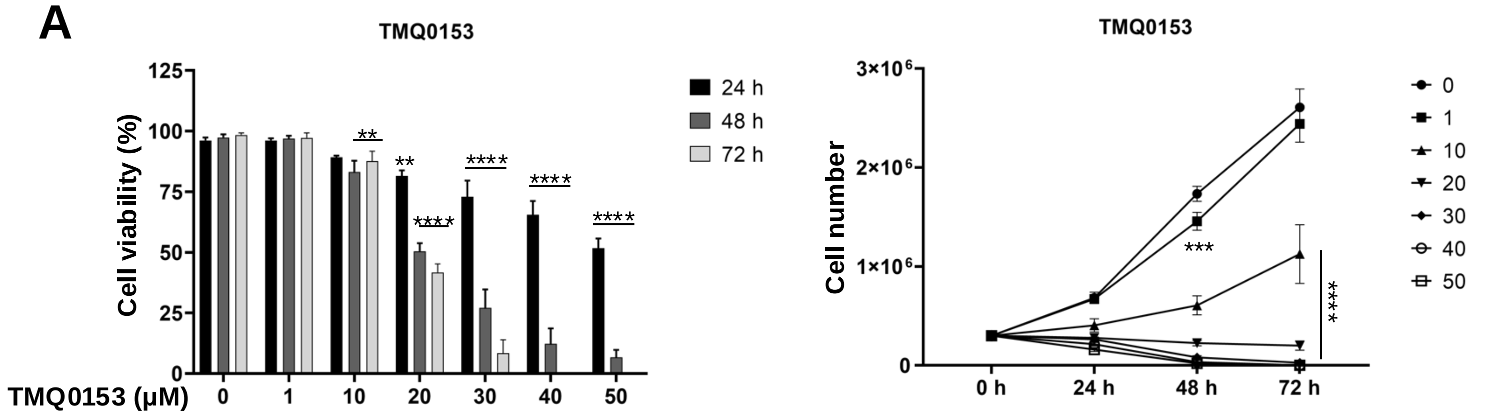

B

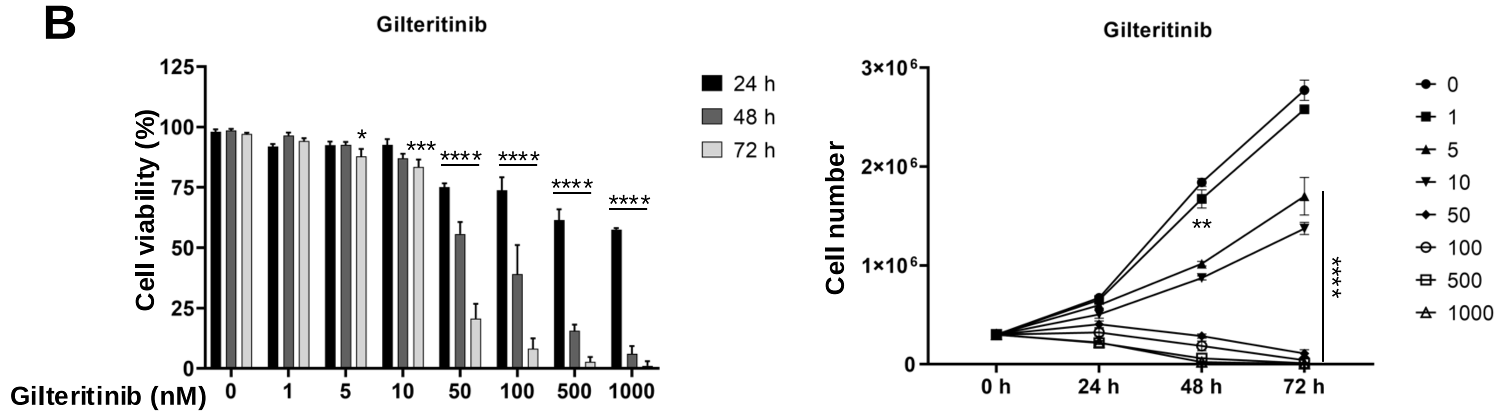

C

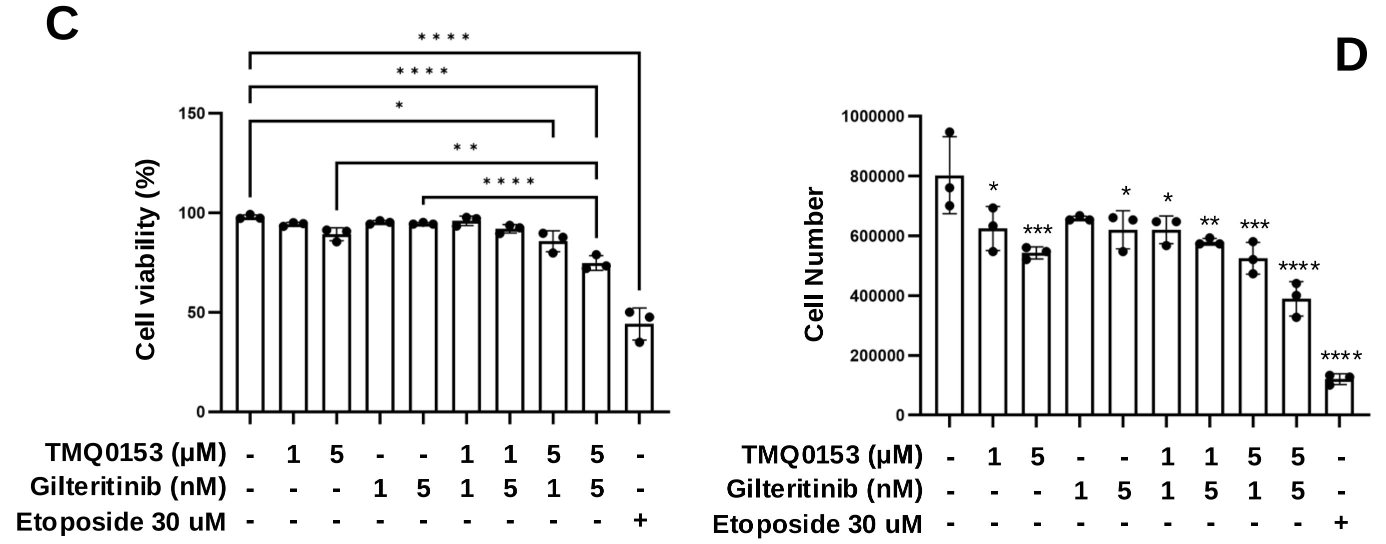

D

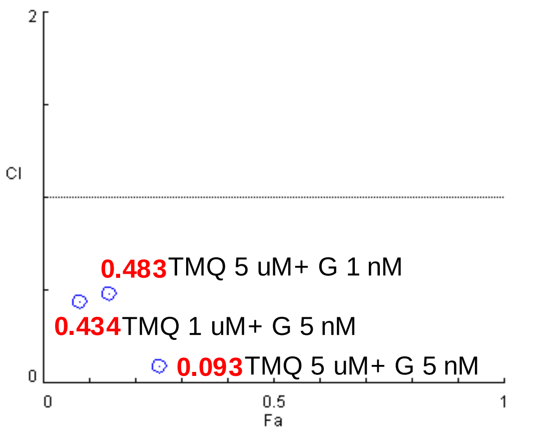

TMQ0153 (μM)	Gilteritinib (nM)	Effect (Fa)	CI
1.0	1.0	0.04	14.3548
1.0	5.0	0.081	0.43489
5.0	1.0	0.143	0.48305
5.0	5.0	0.252	0.09304

E

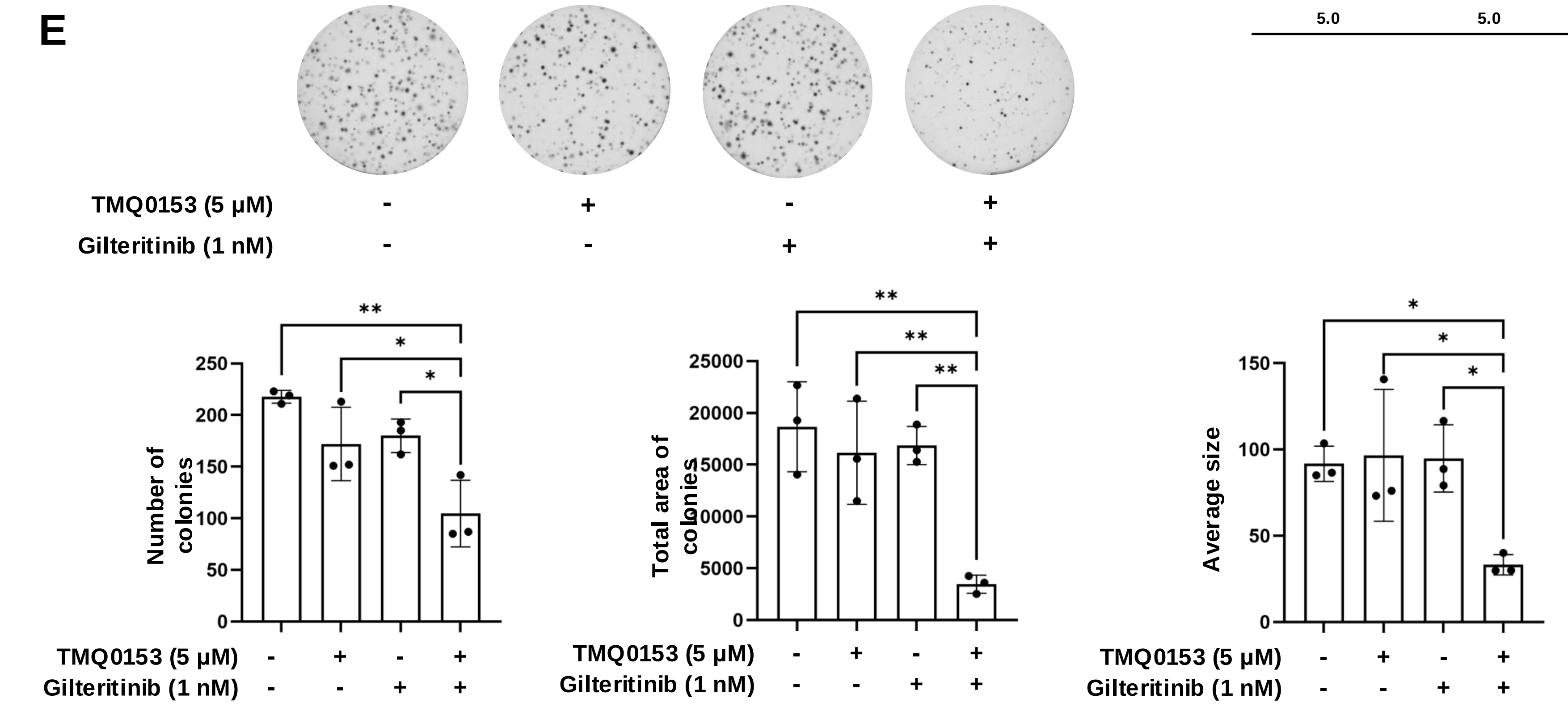

Fig. S22

IC50 (U937)		
	Venetoclax (μM)	Azacitidine (μM)
24 h	8.83±1.78	>30
48 h	3.06±0.34	>30
72 h	1.01±0.12	16.22±7.24

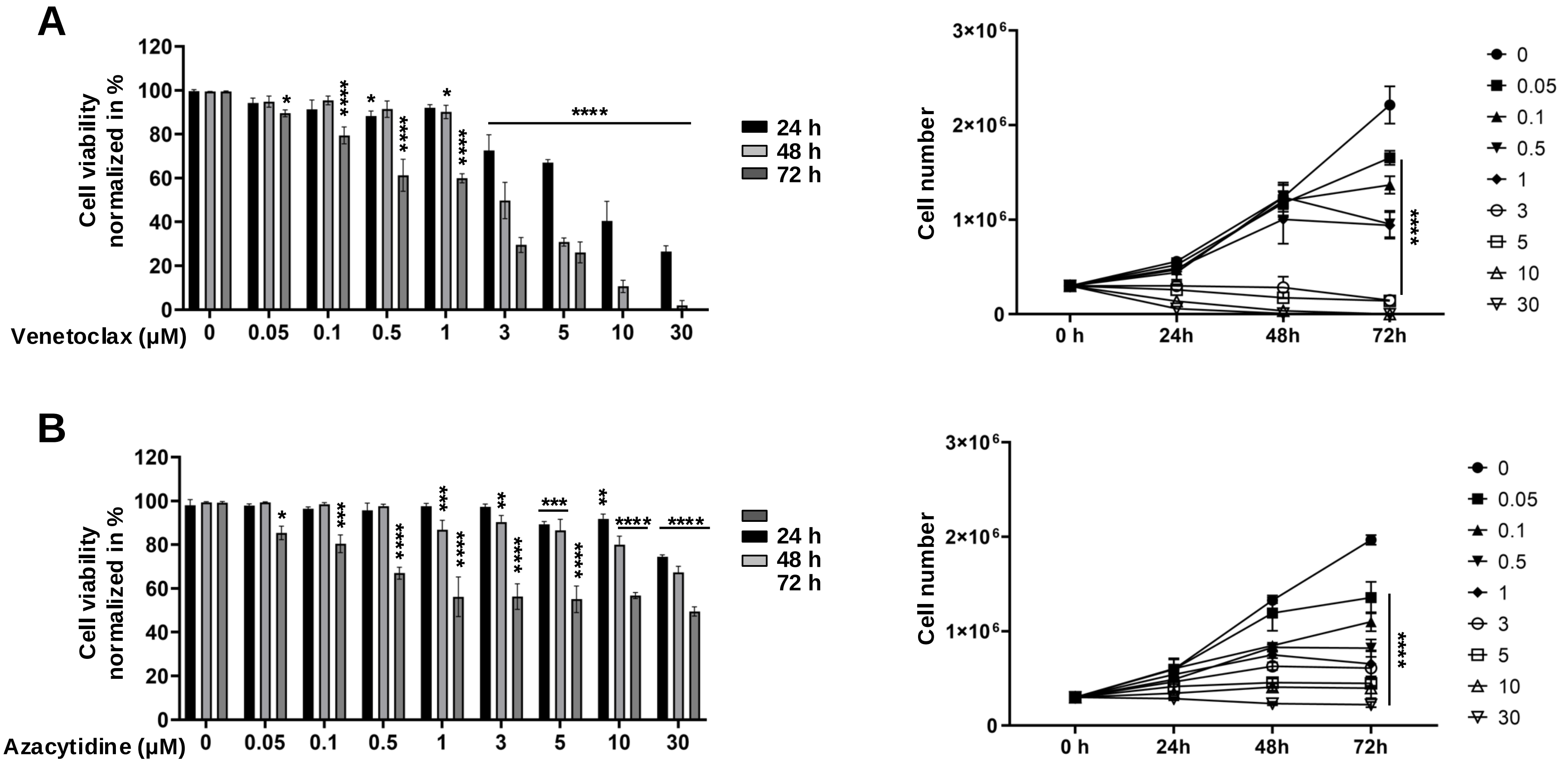

Fig. S23

IC 50 (U937-luc)			
	TMQ0153 (μM)	Azacitidine (μM)	Venetoclax (μM)
24 h	22.06±1.65	22.25±1.52	17.17±2.17
48 h	14.06±0.49	9.31±2.10	7.17±0.54
72 h	13.36±0.32	6.01±1.12	5.28±0.69

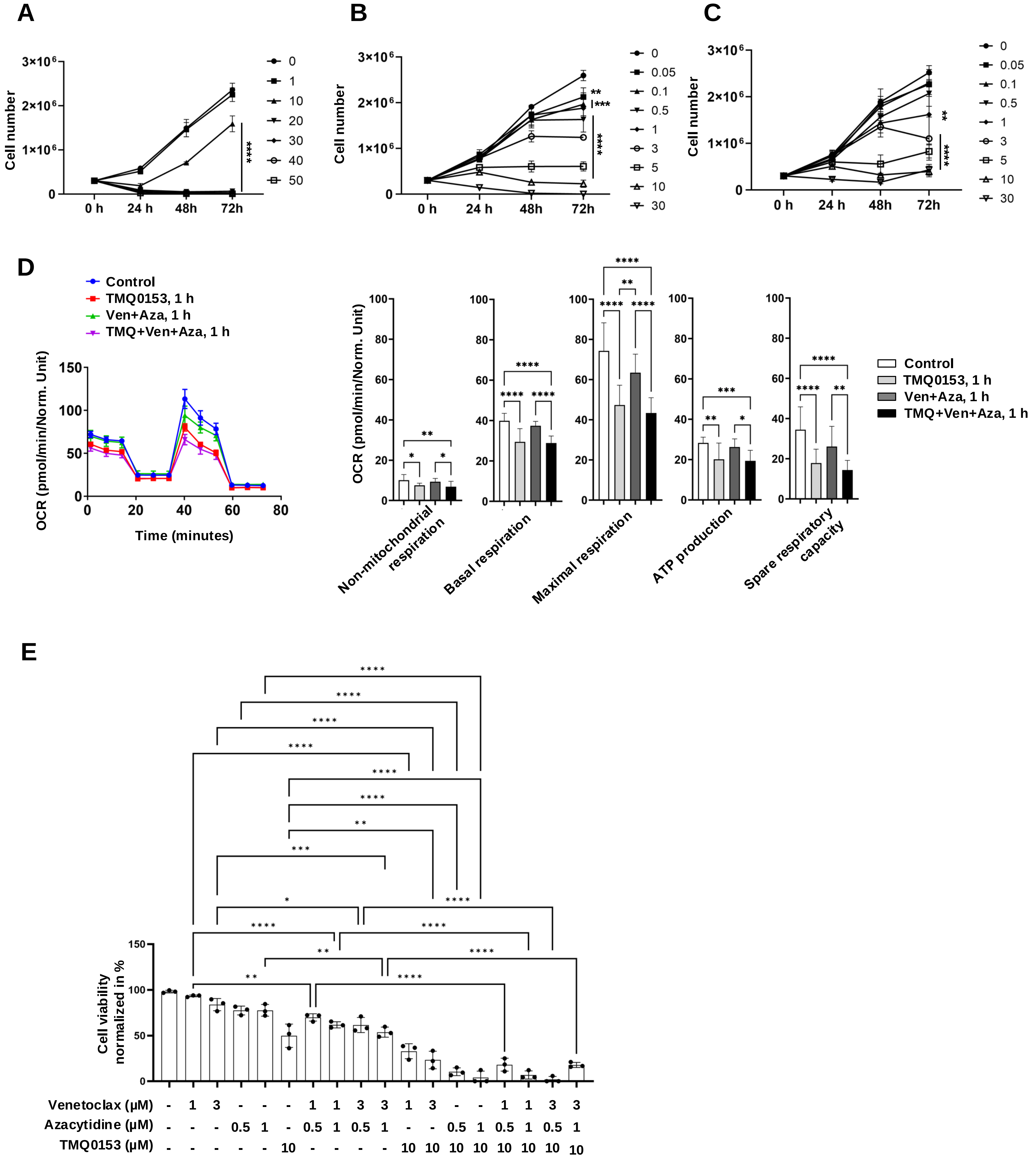

Supplement: Supplementary file 1 — Supplementary Material 1. [file 13046_2025_3372_MOESM1_ESM.pdf]
