# Supplementary material for: Tetrahydrobenzimidazole TMQ0153 targets OPA1 and restores drug sensitivity in AML via ROS-induced mitochondrial metabolic reprogramming
Source: J Exp Clin Cancer Res. 2025 Apr 7;44:114. doi: 10.1186/s13046-025-03372-0 (PMC11974110; doi:10.1186/s13046-025-03372-0)
Supplement: Supplementary file 2 — Supplementary Material 2. [file 13046_2025_3372_MOESM2_ESM.docx]

**Supplementary information**

**Tetrahydrobenzimidazole TMQ0153 Targets OPA1 and Restores Drug Sensitivity in AML via ROS-Induced Mitochondrial Metabolic Reprogramming**

Su Jung Park^1^, Claudia Cerella^2,a^, Jin Mo Kang^1^, Jin Young Byun^3^, David Kum^1^, Barbora Orlikova-Boyer^2,a^, Anne Lorant^2;b^, Michael Schnekenburger^2,a^, Ali Al-Mourabit^4^, Christo Christov^5^, Juyong Lee^3,6^, Byung Woo Han^1^, and Marc Diederich^1,*^

^1^Research Institute of Pharmaceutical Sciences & Natural Products Research Institute, College of Pharmacy, Seoul National University, Seoul 08826, Republic of Korea

^2^Laboratoire de Biologie Moléculaire du Cancer, BAM3 Pavillon 2, 6A rue Nicolas-Ernest Barblé, L-1210 Luxembourg, Luxembourg

^3^College of Pharmacy, Seoul National University, Seoul 08826, Republic of Korea

^4^CNRS, Institut de Chimie des Substances Naturelles, Université Paris-Saclay, F-91190 Gif-sur-Yvette, France

^5^Service d’Histologie, Faculté de Médicine, Université de Lorraine, and INSERM U1256 NGERE, 54000 Nancy, France

^6^Department of Molecular Medicine and Biopharmaceutical Sciences, Graduate School of Convergence Science and Technology, and College of Pharmacy, Seoul National University, Seoul 08826, Republic of Korea

**Running title:** Reprogramming of AML Metabolism Restores Drug Sensitivity

***Corresponding author:** Marc Diederich, Ph.D., Professor, Department of Pharmacy, College of Pharmacy, Seoul National University, Building 21, Room 218, 1 Gwanak-ro, Gwanak-gu, Seoul, 08826, Korea, Office: +82-2-880-8919, Lab: +82-2-880-2490, E-mail: marcdiederich@snu.ac.kr

**Present address:**

^a^ Department of Cancer Research, Luxembourg Institute of Health (LIH), BAM Pavillon 2, 6A rue Nicolas-Ernest Barblé, L-1210 Luxembourg, Luxembourg

**^b^** Luxembourg Centre for Systems Biomedicine, Bioinformatics Core, Roudeneck, 1, Boulevard du Jazz, L-4370 Esch-sur Alzette, Luxembourg

**Conflict of interest:**

The authors declare no potential conflicts of interest.

**Supplementary Information includes:**

Material and methods

Figures Legends for Supplementary Figures 1-S23

Supplementary Tables 1-11

Supplementary References

**Supplementary Material and Methods**

**Cell proliferation and viability assays**

The cell viability and proliferation were analyzed using Trypan Blue staining (Lonza, Walkersville, MD, USA) and a Malassez cell counting chamber (Marienfeld, Lauda-Königshofen, Germany). IC_50_ values for viability were calculated based on the result of the trypan blue assay and shown using Prism 10 software (La Jolla, CA, USA). The type of cell death was determined and quantified by flow cytometry (FACSLyric, BD Biosciences) after Annexin V/ propidium iodide (PI) staining. Caspase activation was assessed by a Caspase activity assay kit (K138-9-25, Biovision, Waltham, MA, USA) following the manufacturer’s protocol.

**Immunoblotting**

Cells were lysed by using M-PER® buffer (ThermoFisher, Waltham, MA, USA) supplemented with 1% PhosStop (Roche, Basel, Switzerland), 1 mM of sodium orthovanadate (JUNSEI, Tokyo), 0.5 % Complete (Sigma-Aldrich, St. Louis, MO, USA), 5 mM sodium fluoride (JUNSEI, Tokyo), 1 mM of phenylmethylsulfonyl fluoride (PMSF). Proteins were resolved by sodium dodecyl sulfate-polyacrylamide gel electrophoresis (SDS-PAGE) and transferred to polyvinylidene fluoride (PVDF) membranes (GE Healthcare Life Science, Chicago, IL, USA). Membranes were incubated with selected primary antibodies (Supplementary Table 1). Luminescence signals were detected with the enhanced luminol-based chemiluminescent (ECL) Plus Western Blotting Detection System (GE Healthcare Life Science, Chicago, IL, USA), and bands were acquired using an Amersham Imager 600 (GE Healthcare Life Science, Chicago, IL, USA). Band intensity was quantified by Image J 1.54d software (US National Institute of Health, Bethesda, MD, USA, RRID:SCR_003070).

**Measurement of mitochondrial membrane potential and cytochrome C release**

For mitochondrial membrane potential (MMP) analysis, cells were incubated at 37 ℃ for 25 min with 10 nM Mito-Tracker Red CMXRosRed (Molecular Probes, Invitrogen, Grand Island, NY, USA). Cells were resuspended with phenol red-free RPMI containing 1 % Penicillin-Streptomycin and analyzed by flow cytometry (FACSLyric, BD, Biosciences). Data were analyzed using the Flow-Jo 10.9 software (RRID:SCR_008520).

To assess mitochondrial cytochrome c levels, we employed a flow cytometry protocol (1) to ensure selective permeabilization of the cytoplasmic and nuclear membranes while maintaining mitochondrial integrity. After treatment with TMQ0153, cells were collected by centrifugation at 800 × g for 5 minutes at room temperature. The supernatant was discarded, and the cell pellet was resuspended in 2 mL of permeabilization buffer (5 mM KCl, 0.005% digitonin in PBS) to selectively permeabilize the cytoplasmic and nuclear membranes, leaving the mitochondrial membranes intact. The suspension was incubated on ice for 7.5 minutes, followed by centrifugation at 1,200 × g for 5 minutes to remove the supernatant. The resulting pellet was resuspended in 400 µL of 4% paraformaldehyde in PBS for fixation and incubated at room temperature in the dark for 20 minutes. After washing twice with PBS, the pellet was resuspended in 1 mL of PBS and stored at 4°C in the dark. Blocking was performed by resuspending the pellet in 400 µL of blocking buffer (3% BSA, 0.05% saponin in PBS) for 1 h at room temperature. After blocking, the pellet was centrifuged at 2,400 × g for 5 minutes, and the supernatant was discarded. For primary antibody binding, the cell pellet was resuspended in 100 µL of antibody binding buffer (cytochrome c antibody [Santa Cruz, Dallas, TX, USA], diluted 1:50 in blocking buffer) and incubated overnight at 4°C in the dark with gentle agitation. Following incubation, the cells were washed with 1 mL of PBS and centrifuged at 2,400 x g for 5 minutes. The pellet was then resuspended in 100 µL of secondary antibody buffer containing Alexa Fluor 568-conjugated anti-rabbit IgG (diluted 1:100 in blocking buffer) and incubated for 1 h at room temperature in the dark. After staining, cells were washed with 1 mL of cold PBS and resuspended in 200 µL of PBS. Flow cytometric analysis was performed using a FACSLyric instrument (BD Biosciences). Data acquisition and analysis were conducted using FlowJo 10.9 software.

**Analysis of ROS generation, NAD^+^/NADH, and GSH/GSSG ratios**

DCFDA/H_2_DCFDA-Cellular ROS assay kit (Abcam, Cambridge, MA, USA) was used following the manufacturer’s instructions to measure cellular ROS generation. The fluorescence signals were detected by a SpectraMax i3x microplate reader, and data were recorded using the SoftMax Pro 7.0 software package (Sunnyvale, California, USA). The whole cell NAD+/NADH ratio was analyzed using the NAD^+^/NADH Glo assay kit (Promega Madison, WI, USA). GSH and GSSG measurements were performed using the GSH/GSSG-Glo™ Assay kit (Promega Madison, WI, USA) following the manufacturer’s instructions.

**Determination of the oxygen consumption rate and glycolytic rate**

Mitochondrial respiration was assessed by measuring the oxygen consumption rate (OCR) using the Agilent Seahorse XF Cell Mito Stress Test Kit (#103015-100, Agilent, Santa Clara, CA, USA) with a Seahorse XFe96 analyzer (Agilent). Cells were seeded in 6-well plates at a density of 300,000 cells/mL in 3 mL of complete RPMI1640 medium. After one hour, cells were treated with 20 μM TMQ0153 for the different time points to allow simultaneous harvesting. Cells were then counted, washed three times in 1X PBS, and transferred to a Seahorse 96-well plate. MV4-11 cells were seeded at 1 × 10⁵ cells per well in a total volume of 180 µL of Seahorse XF RPMI medium pH 7.4. We used the standard injection sequence of the Cell Mito Stress Test Kit after optimization of the concentrations of the different inhibitors: 1.5 μM oligomycin, 1.0 μM carbonyl cyanide 4-(trifluoromethoxy) phenylhydrazone (FCCP), and 0.5 μM rotenone/antimycin A. U937 cells were seeded at 1.7 × 10⁵ cells per well in 180 µL of Seahorse XF RPMI medium pH 7.4. We used the standard injection sequence of the Cell Mito Stress Test Kit after optimization of the concentrations of the different inhibitors: 1.5 μM oligomycin, 0.125 μM FCCP, and 0.5 μM rotenone/antimycin A. Data analysis was performed using the Seahorse Wave software (Agilent, Santa Clara, CA, USA).

To investigate the metabolic shift from oxidative phosphorylation (OXPHOS) to glycolysis after drug treatment, the proton efflux rate was measured using a Seahorse XF Glycolytic Rate Assay Kit (#103344-100, Agilent, Santa Clara, CA, USA). 0.5 μM rotenone/antimycin A and 50 mM 2-deoxy-D-glucose (2-DG) were used for both cell lines. Data were analyzed using the Seahorse Wave software (Agilent, Santa Clara, CA, USA).

**Transmission electron microscopy (TEM)**

For mitochondrial morphological analysis, 10^7^ cells were prepared and pelleted. Cells were fixed in Karnovsky’s fixative solution overnight at 4 ℃. The cells were rinsed with sodium cacodylate buffer and post-fixed in 1 % osmium tetroxide diluted in 0.1 M sodium cacodylate buffer. Samples were washed with distilled water and then dehydrated through a graded series of ethanol solutions (30 %~100 %) followed by propylene oxide. 1:1 propylene oxide/Spurr’s resin mixture was used for the transition step. Samples were embedded in 100 % Spurr’s resin and left to polymerize in an oven at 56 ℃ for 48 h. Ultrathin sections (70–90 nm) were obtained with an ultramicrotome, EM UC7 (Leica, Wetzlar, Germany). Sections were stained with uranyl acetate and lead citrate and examined with a JEM1010 transmission electron microscope (JEOL, Tokyo, Japan).

**Immunohistochemistry (IHC)**

After the xenograft study using MV4-11, the tumors were collected and stored in 10 % formalin for IHC analysis. The paraffin blocks and embedding in the slides were performed by LOGONE Bio (Seoul, Korea) soaked in xylene and ethanol to remove the paraffin and treated with trypsin antigen retrieval solution (Ab970, Abcam, Cambridge, MA, USA). Endogenous peroxidase was eliminated by hydrogen peroxide (3 %). The samples were incubated with anti-Ki-67 (1:200) and cleaved-caspase-3 (1:200) overnight at 4 ℃. Secondary antibodies were incubated for 1 h, and avidin-biotin conjugate solution was used according to the manufacturer’s instructions (Vectastain, PK-4001, Vector Laboratories, CA, USA). The tissue sections were incubated with 3,3'-diaminobenzidine (DAB) for visualization.

**siRNA transfection**

For the experiment, 2x10^5^ K562 cells were seeded in a 24-well plate with 100 µl of RPMI containing 10% FBS and 1% penicillin-streptomycin. The transfection reagent was prepared using 100 nM of siRNA and 6 µl of HiPerFect transfection reagent (QIAGEN, Hilden, Germany) in 100 µl of culture media without serum, following the manufacturer’s instructions. Control siRNA (AllStar Negative, 1027280, QIAGEN), FlexiTube siRNA for OPA1 (SI03019429 and SI40500088, QIAGEN) were used. The cells were incubated for 48 h and underwent a second transfection for another 24 h.

**Real time-PCR**

To confirm the knockdown of OPA1, total RNA was extracted using the RNeasy Mini Kit (QIAGEN). For the RT-PCR, iTaq Universal SYBR Green Supermix (Bio-Rad, CA, USA) was utilized along with OPA1 primers: Forward (F) 5’-GCTGCGCAGACCATGAATTC-3’ and Reverse (R) 5’-GGTCTCCCAAGCAACCTCTAC-3’. GAPDH served as a housekeeping gene, with primers F: 5’-CTGGGCTACACTGAGCACC-3’ and R: 5’-AAGTGGTCGTTGAGG GCAATG-3’. The fold change values were calculated.

**In vivo xenografts**

Balb/c nude mice (RRID: IMSR_RJ:BALB-C-NUDE), 6–8 weeks old and weighing 18–20 g, were obtained from Orient Bio Inc. (Seoul, Korea). All animal experiments were approved by the Seoul National University Institutional Animal Care and Use Committee (SNU-220704-6). To suppress the immune system, mice were pre-treated with busulfan (20 mg/kg) via intraperitoneal injection 24 h before cancer cell inoculation. MV4-11 cells (5 × 10⁶) were mixed with Geltrex (Thermo Fisher Scientific, Waltham, MA, USA) at a 1:1 ratio and injected subcutaneously. Once tumor nodules reached a size of 100–150 mm³, mice were randomly assigned to either the control group (n=6) or the TMQ0153 treatment group (20 mg/kg, n=6).

In a separate experiment, Balb/c nude mice were exposed to 2.5 Gy of X-RAD irradiation 24 h prior to tumor injection to enhance immunosuppression. MV4-11 cells (5 × 10⁶) were again mixed with Geltrex in a 1:1 ratio and subcutaneously injected. When tumors reached 100–150 mm³, mice were randomized into the control (n=9) and TMQ0153 (50 mg/kg, n=9) groups. Tumor dimensions were measured with calipers, and volumes were calculated using the formula: V = 1/2 × length × (width)². TMQ0153 was administered intraperitoneally (i.p.) every three days. The study was terminated when tumors in the control group reached 2,000–2,500 mm³.

To evaluate systemic toxicity, blood tests were conducted using the FUJI DRI-CHEM 3500s analyzer (FUJIFILM, Tokyo, Japan). Markers for organ damage included glutamic pyruvic transaminase (GPT), glutamic-oxaloacetic transaminase (GOT), blood urea nitrogen (BUN), and creatinine (CREA). These parameters provided a comprehensive assessment of organ function and potential adverse effects of the treatment.

**Binding site prediction**

To identify potential ligand-binding sites for TMQ derivatives, we utilized PrankWeb (version v3.0, Charles University, Czech Republic) (2), an online resource that interfaces with P2Rank (version v2.5, Charles University, Czech Republic) (3), a machine learning-based tool for ligand-binding site prediction. We submitted protein structures modeled by AlphaFold3 (version v3, Google DeepMind and Isomorphic Labs, UK) (4) to the PrankWeb server, which predicts ligandable sites for a given protein structure. PrankWeb resulted in the predicted pockets for the input PDB structure. For each predicted pocket, PrankWeb provided the estimated score and probability. From the resulting predictions, we selected the site with the highest probability score.

**Molecular dynamics simulations**

The topology files for the OPA1 and the TMQ ligand derivatives were generated using the tleap and Antechamber programs implemented in AMBER24 (version v24, University of California, USA) (5), employing the ff14SB force field for the protein and the Generalized AMBER Force Field (GAFF) for the ligand. The systems were solvated using the TIP3P water model with a 12 Å padding region. Initial energy minimization of the solvated systems was performed to remove any unfavorable contacts. This process involved 10,000 cycles, with the first 5,000 cycles employing the steepest descent method, followed by 5,000 cycles using the conjugate gradient method. Following minimization, equilibration molecular dynamics (MD) simulations were conducted as follows: 10 ns under constant volume (NVT) and 100 ns under constant pressure (NPT) with positional restraints: 10.0 kcal·mol⁻¹Å⁻² for minimization step, 2.0 kcal·mol⁻¹Å⁻² for NVT equilibration step, and 1.0 kcal·mol⁻¹Å⁻² for NPT equilibration step, respectively. A time step of 2 fs was used for the MD simulations, with all bonds involving hydrogen atoms constrained using the SHAKE algorithm. Long-range electrostatic interactions were calculated using the particle mesh Ewald (PME) method. Temperature control was maintained with a Langevin thermostat. All simulations were performed at a constant temperature of 300 K. Subsequently, production simulations were carried out for 500 ns under NPT conditions, with trajectories recorded every 2 ps.

**Supplementary figures**

**Fig. S1** Pan-cancer gene expression analysis of factors involved in mitochondrial fission and fusion compared with samples from corresponding non-cancerous specimens. (**A-E**) Differential gene expression of (**A**) dynamin-1-like protein (DNM1L;DRP1), (**B**) mitofusin (MFN)1, (**C**) MFN2, (**D**) fission protein 1 (FIS1), and (**E**) mitochondrial fission factor (MFF) genes in hematological and solid tumor patients were compared to healthy samples using data available from the TNMplot website. Mann-Whitney test; **P* < 0.05; ***P* < 0.01, ****P* < 0.001, *****P* < 0.0001. ns; non-significant. (**F**) OPA1 and DNM1L (DRP1) gene expression correlation analysis from the TCGA LAML cohort was extracted from GEPIA.

**Fig. S2** Pro-cancer features of increased pro-fusion and pro-fission mitochondrial factors. (**A**&**B**) Comparative analysis of the expression of the selected genes between samples from healthy donors (BM: bone marrow, MNCs: mononuclear cells) and AML patients included in (**A**) the MILE Study and (**B**) the Beat AML 2.0 cohorts. In (**A**), the study also included samples available in the MILE Study from patients affected by myelodysplastic syndrome (MDS). (**C**) Expression of selected genes in AML patient samples from Beat AML 2.0 cohort stratified by the disease stage at the specimen collection. (**D**) Paired analysis of OPA1 expression in AML patient samples taken at the diagnosis vs. residual disease stages from the Beat AML 2.0 cohort. Medians were compared by the Kruskal-Wallis test; comparisons between two subgroups or between the median of each subgroup and the overall median (dashed line) were performed applying the Mann-Whitney test (&/$/* *P* < 0.05; &&/##/$$/***P* < 0.01, &&&/###/$$$/****P* < 0.001, &&&&/####/$$$$/**** *P* < 0.0001). ns; non-significant.

**Fig. S3** (**A**) Paired diagnostic (primary) vs. relapse (recurrent) analysis of 15 pediatric AML patients from the TARGET AML cohort of the expression of the indicated selected genes. The two patient groups identified by red and blue colors correspond to the two groups showing distinct OPA1 expression trends between the two disease stages of Figure 1F (paired t-test; **P* < 0.05; ***P* < 0.01, ****P* < 0.001).ns; non-significant. (**B-D**) The top 20 gene ontology terms for Biological Processes (BP), Molecular Functions (MF), and Cellular Components (CC) found to be enriched between the selected patient groups of **Fig. 1F** are shown.

**Fig. S4** OPA1 expression in AML patients stratified by specific clinical features. (**A-C**) Analysis of OPA1 expression in AML patients stratified by risk (**A**), French-American-British (FAB) classification (**B**), and FMS-like tyrosine kinase 3 (FLT3) mutational status (**C**). Cohorts: adult TCGA (N=179), Beat AML 2.0 (N=596), Leucegene (N=387), Verhaak (GSE6891; N=437), and pediatric TARGET-AML (N=145). Redundant samples from the same patients in each cohort were excluded from the analysis. Medians were compared by the Kruskal-Wallis test; further comparisons between the median of each subgroup and the overall median (dashed line) were performed applying the Mann-Whitney test (*P* values: * < 0.05, **< 0.01, ***< 0.001, **** < 0.0001). ns; non-significant.

**Fig. S5** TMQ0153 induces mitochondrial shrinkage and disorganization of the mitochondrial matrix. (**A&B**) Mitochondrial morphology was analyzed by transmission electron microscopy (TEM) in MV4-11 (**A**) and U937 (**B**) cells following TMQ0153 treatment for 24 h. The mitochondrial area (nm²) per unit length (nm) was quantified and presented. Data were analyzed using an unpaired t-test. Statistical significance is indicated as *P < 0.05, **P < 0.01, ***P < 0.001, ****P < 0.0001 compared to control cells.

**Fig. S6** Measurement of activities of mitochondria complexes after TMQ0153 treatment at various concentrations using Mitocheck® kit. (**A-D**) Complex I (**A**), II/III (**B**), IV (**C**), and V (**D**) activities were calculated following the manufacturer’s instructions. All data represent the mean ± SD at least three independent experiments. Data analysis was done using One-Way ANOVA and Dunnett’s test. **P* < 0.05, ***P* < 0.01, ****P* < 0.001, *****P* < 0.0001 compared to control cells. ns; non-significant.

**Fig. S7** Anti-cancer effect of TMQ0153 on AML cell lines. (**A-B)** The effect of TMQ0153 on the cell viability and proliferation of MV4-11 and U937 cell lines. Trypan blue staining showed the dose (0, 1, 10, 20, 30, 40, and 50 μM)- and time-dependent (12, 24, 48, and 72 h) cytotoxic effect of TMQ0153 on MV4-11 (**A**) and U937 (**B**) cells. (**C-D)** The number of viable MV4-11 (**C**) and U937 (**D**) cells after TMQ0153 treatment is represented. (**E-H)** Annexin V/PI staining was performed after TMQ0153 treatment by FACS analysis after 6 h cells (**E** and **F**) and 24 h (**G** and **H**) in MV4-11 and U937. IC50 values were determined using the results from Annexin V/PI assays. Cells were pretreated with 50 μM z-VAD for 1 h before TMQ0153 treatment. 30 μM of etoposide for 24 h and 500 nM of midostaurin for 24 h were used as positive controls for cell death induction. All data represent the mean ± SD of at least three independent experiments. Data analysis was done using one-way ANOVA and Dunnett’s test. Statistical significance was assessed as **P* < 0.05, ***P* < 0.01, ****P* < 0.001, *****P* <0.0001 compared to untreated cells.

**Fig. S8** TMQ0153 treatment blocks metabolic pathways in AML cells. (**A**&**B**) RNA sequencing was conducted after 20 μM TMQ0153 treatment in MV4-11 (control, 1 h, and 6 h) and U937 (control, 1 h, and 48 h) cells. The expression patterns of the 69 genes across four categories (Metabolism, Mitochondria, OXPHOS, ROS) are shown for MV4-11 (**A**) and U937 (**B**) cells. The heat map illustrates the mRNA levels differentially expressed across the three time points. Volcano plot shows differentially expressed genes with |log2FC(Fold Change) > 1 and adjusted *P*-value < 0.05. Genes identified as upregulated are represented in red, while downregulated genes are shown in blue.

**Fig. S9** Treemap and Bubble Plot of GO Enrichment Analysis in MV4-11 and U937 cells. (**A**&**B**) The treemap and bubble plot illustrate the Gene Ontology (GO) enrichment analysis results for differentially expressed genes. The analysis was visualized using REViGO, clustering GO terms to highlight key biological processes. In the treemap, each block represents a GO term, and the size corresponds to its significance level. (**C**&**D**) In the bubble plot, each bubble represents a GO term, with size indicating enrichment significance and color representing *P*-value. Major biological processes include metabolic processes, cellular response, and macromolecule metabolism in MV4-11 (**A**&**B**) and U937 (**C**&**D**) cell lines.

**Fig. S10** TMQ0153 impaired colony formation ability in AML cell lines. (**A-D**) Cells were treated with 0, 1, 10, 20, and 50 μM TMQ0153 and incubated with methylcellulose for 10 days. The colony formation was assessed by MTT assay in MV4-11 (**A**), U937 (**B**), HL60 (**C**), and THP-1 (**D**) cells. Pictures are representative of three independent experiments. The quantification was analyzed by image J. All data represent the mean ± SD of at least three independent experiments. Data analysis was done using one-way ANOVA and Dunnett’s test. Statistical significance was assessed as **P* < 0.05, ***P* < 0.01, ****P* < 0.001, *****P* <0.0001 compared to untreated cells.

**Fig. S11** Molecular dynamic analysis of TMQ candidates with OPA1 at the GDP binding site. **A** Four predicted ligand binding sites were identified by P2Rank. Each site is color-coded based on its probability score: Rank 1 in red (0.858), Rank 2 in green (0.291), Rank 3 in orange (0.163), and Rank 4 in magenta (0.042). The high probability of the top-ranked site suggests it is the most likely binding location for TMQ derivatives. **B** Root Mean Square Deviation (RMSD) of systems over the simulation time. To ensure an accurate assessment of TMQ57's binding stability, only the trajectory segments where the ligand remained within the binding site were included in the RMSD analysis. Time (ps) corresponding to post-dissociation behavior were excluded. The result is representative of three independent experiments.

**Fig. S12** Dose-dependent effect of TMQ0153 on mitochondrial dynamic proteins in AML and CML cell lines. **A-B** AML (MV4-11 and U937) (**A**) and CML (K562 and K562IR) (**B**) cells were treated with 0, 1, 10, and 20 μM of TMQ0153 for 24 h, and mitochondrial dynamic-related protein levels were estimated by western blot. Blots are representative of three independent experiments. Data represents the mean ± SD of at least three independent experiments. Data analysis was done using one-way ANOVA and Tukey’s test. **P* < 0.05, ***P* < 0.01, ****P* < 0.001, *****P* < 0.0001.

**Fig. S13** Knock-down of OPA1 in K562 cells. (**A-B)** OPA1 knock-down by siRNA in K562 cells was confirmed by real-time PCR. Results show the fold change of OPA1 mRNA levels compared to the AllStar control (**A**) and resulting quantified changes in OPA1 expression by western blot (**B**). **C-D** Cell viability (**C**) and proliferation (**D**) were measured by trypan blue staining. (**E)** ROS generation was assessed after OPA1 knock-down. (**F)** The effect of OPA1 depletion on mitochondrial function was measured by the Seahorse MitoStress assay kit. Anti- and pro-apoptotic protein levels change after the knock-down of OPA1 in K562 cells. (**G)** After the OPA1 knock-down, myeloid cell leukemia sequence 1 (Mcl-1), B-cell lymphoma-extra large (Bcl-xL), and BH3 interacting domain death agonist (Bid) protein were assessed by western blot. Band intensities were analyzed by Image J. Blots are representative of three independent experiments. Data represents the mean ± SD of at least three independent experiments. Data were analyzed using One-Way ANOVA and Tukey’s test. **P* < 0.05, ***P* < 0.01, ****P* < 0.001, *****P* < 0.0001. ns; non-significant.

**Fig. S14** **Flow cytometry analysis of mitochondrial cytochrome c levels. (A&B)** Histograms show anti-cytochrome c-FITC fluorescence intensity in MV4-11 (**A**) and U937 (**B**) cells treated with TMQ0153 (20 μM for 1 h, 3 h, and 6 h), with or without pre-treatment with NAC (1 mM, 1 h). Etoposide (30 μM, 24 h) was used as a positive control to induce cytochrome c release. Histograms are representative of three independent experiments.

**Fig. S15** Co-staining of cytochrome c and MitoTracker Green observed by confocal microscopy. (**A**) MV4-11 cells were treated with TMQ0153 (20 μM, 3 h). The nucleus was stained with DAPI (blue), cytochrome c with an anti-cytochrome c antibody (red), and mitochondria with MitoTracker Green (green). Mean fluorescence intensity (MFI) is shown in the right panel. (**B**) U937 cells were treated with TMQ0153 (20 μM, 3 h), followed by cytochrome c and mitochondrial staining, as performed for MV4-11 cells. Data were analyzed using an unpaired t-test. Statistical significance is indicated as *P < 0.05, **P < 0.01, ***P < 0.001, ****P < 0.0001.

**Fig. S16** Comparison of cellular metabolites between MV4-11 and U937 cell lines. The data was explored on the Cancer Cell Line Encyclopedia (CCLE) dataset (https://depmap.org/portal/ccle/). The heat map indicated the comparison of metabolites in MV4-11 and U937 cells.

**Fig. S17** TMQ0153 activates apoptotic mitochondrial pathway and caspase-dependent cell death in AML cell lines. (**A**) Western blot analysis of Mcl-1, Bcl-xL, Bcl-2, and Bid proteins after 20 μM of TMQ0153 treatment at 1, 3, and 6 h. Etoposide (Eto; 30 μM for 6h) was used as a positive control. (**B**&**C**) Caspase activation was assessed by a Caspase activity assay kit after 20 μM TMQ0153 treatment for 6 h in MV4-11 (**B**) and U937 (**C**) cell lines. (**D**&**E**) MV4-11 (**D**) and U937 (**E**) cells were treated with TMQ0153 (20 μM) for 1 h, 3 h, and 6 h. Caspase 8, 3, and 9 were detected by western blot. Etoposide (Eto) 30 μM at 6 h was used as a positive control. (**F**) TMQ0153 (20 μM, 6 h)-mediated protein cleavage was prevented by Z-VAD (50 μM) pre-treatment for 1 h. Blots are representative of three independent experiments. All data represent the mean ± SD of at least three independent experiments. D; DMSO, T; TMQ0153. Data was analyzed using One-Way ANOVA and Tukey’s test. * *P* < 0.05, ***P* < 0.01, ****P* < 0.001, *****P* < 0.0001.

**Fig. S18.** Histological analysis from an acute toxicity study in six-week-old C57BL/6 mice receiving a single intraperitoneal injection of TMQ0153 at doses of 1, 10, 20, and 50 mg/kg. Key organs, including the heart, spleen, lungs, liver, and kidneys, were stained with hematoxylin and eosin (H&E) for histological evaluation.

**Fig. S19** In vivo evaluation of TMQ0153 in an MV4-11 xenograft mouse model. Six-week-old Balb/c nude mice were pre-treated with busulfan (20 mg/kg) one day prior to tumor cell injection. (**A**) The experimental setup is illustrated in the panel. After tumor volumes reached approximately 150–200 mm³, TMQ0153 (20 mg/kg) was administered intraperitoneally every three days. (**B**) Tumor weights at the time of sacrifice. (**C**) Body weight changes were monitored throughout the study. (**D**) Images of isolated tumors. (**E**) In a separate experiment, six-week-old Balb/c nude mice were exposed to X-ray irradiation (2.5 Gy) one day prior to tumor injection, as depicted in the panel. (**F**) TMQ0153 was administered at a higher dose (50 mg/kg) every three days. Tumor volumes were monitored over time. (**G**) Body weights were recorded regularly. (**H**&**I**) On the final day of the experiment (day 24), tumor volumes (**H**) and weights (**I**) were measured. (**J**) Mice survival data are presented. Statistical analysis was performed to assess treatment effects. Tumor weight data were analyzed using the Mann-Whitney test (**B**), while survival data were evaluated using the Log-rank (Mantel-Cox) test (**J**). Statistical significance is indicated as follows: **P* < 0.05, ***P* < 0.01, ****P* < 0.001, *****P* < 0.0001, and “ns” for non-significant comparisons relative to vehicle-treated mice.

**Fig. S20.** The in vivo anti-cancer effect of TMQ0153 in an MV4-11 xenograft study. Six-week-old Balb/c nude mice were exposed to X-ray irradiation (2.5 Gy) one day before tumor injection. (**A**) The experimental scheme is illustrated. TMQ0153 (35 mg/kg) was administered when tumors reached approximately 150–200 mm³. Individual (**B**) and average (**C**) body weights were monitored. (**D&E**) Individual (**D**) and average (**E**) tumor volumes were measured. Tumor volume (**F**) and weight (**G**) were assessed on the day of sacrifice (day 24). (**H**) Representative images of isolated tumors are shown. (**I**) Blood was collected from the heart, and serum was isolated for biochemical analysis of GTP, GOT, BUN, and CRE using FUJI DRI-CHEM. (**J**) Tumor tissues were stained for H&E, Ki-67, and cleaved caspase-3. Statistical significance was assessed as *P < 0.05, **P < 0.01, ***P < 0.001, ****P < 0.0001, and “ns” for non-significant comparisons relative to vehicle-treated mice. Data were analyzed using the Mann-Whitney test.

**Fig. S21** Anti-cancer effects of TMQ0153 and gilteritinib on MOLM-14 cells. (**A**&**B**) MOLM-14-luc cells were treated with increasing concentrations of TMQ0153 (**A**) or gilteritinib (**B**) for up to 72 h, and the cell viability and proliferation were assessed by trypan blue staining assay. (**C**) The effect of combination treatment (24 h) on cell viability and cell proliferation in MOLM-14-luc cells. (**D**) The synergistic effect was analyzed using Compusyn software (CI; combination index, Fa; fraction affected). (**E**) A colony formation assay was conducted after single or combination treatment in the MOLM-14-luc cells. Pictures are representative of three independent experiments. The number of colonies, total area of colonies, and average size are indicated. All data represent the mean ± SD in at least three independent experiments. Data were analyzed using one-way ANOVA and Dunnett’s (**A**&**B**) or Tukey test (**C**&**E**). **P* < 0.05, ***P* < 0.01, ****P* < 0.001, *****P* < 0.0001.

**Fig. S22** The effect of venetoclax and azacitidine in U937 cells. (**A**&**B**) U937 cells were treated with venetoclax (**A**) and azacitidine (**B**) at different concentrations and incubation times, and the cell viability was assessed by trypan blue staining assay. Cell proliferation was shown in the right panel. All data represent the mean ± SD of at least three independent experiments. Data were analyzed using One-Way ANOVA and Dunnett’s test. **P* < 0.05, ***P* < 0.01, ****P* < 0.001, *****P* < 0.0001.

**Fig. S23** The effect of venetoclax and azacitidine in U937-luc cells. (**A-C**) U937-luc cells were treated with increasing concentrations of TMQ0153 (**A**), venetoclax (**B**), and azacitidine (**C**) for up to 72 h, and cell proliferation was assessed by trypan blue staining assay. (**D**) Seahorse MitoStress Assay was conducted after 1 h of TMQ0153 (10 μM; TMQ), venetoclax (3 μM; Ven), and azacitidine (0.5 μM; Aza) treatment. (**E**) The cell viability was assessed after single or combination treatment for 48 h. All data represent the mean ± SD of at least three independent experiments. Data were analyzed using One-Way ANOVA and Dunnett’s test. **P* < 0.05, ***P* < 0.01, ****P* < 0.001, *****P* < 0.0001.

**Supplementary Tables**

**Table S1. Physicochemical properties of TMQ0153.**

| **Physicochemical Properties** | |
| --- | --- |
| Formula | C14H13ClN2O2 |
| Molecular weight | 276.72 g/mol |
| Num. heavy atoms | 19 |
| Num. arom. heavy atoms | 6 |
| Fraction Csp3 | 0.29 |
| Num. rotatable bonds | 1 |
| Num. H-bond acceptors | 3 |
| Num. H-bond donors | 1 |
| Molar Refractivity | 81.14 |
| TPSA | 52.90 Å² |
| **Lipophilicity** | |
| Log *P*_o/w_ (iLOGP) | 1.43 |
| Log *P*_o/w_ (XLOGP3) | 0.95 |
| Log *P*_o/w_ (WLOGP) | 1.40 |
| Log *P*_o/w_ (MLOGP) | 1.89 |
| Log *P*_o/w_ (SILICOS-IT) | 2.41 |
| Consensus Log *P*_o/w_ | 1.62 |
| **Water Solubility** | |
| Log *S* (ESOL) | -2.32 |
| Solubility | 1.32e+00 mg/ml ; 4.77e-03 mol/l |
| Class | Soluble |
| Log *S* (Ali) | -1.65 |
| Solubility | 6.22e+00 mg/ml ; 2.25e-02 mol/l |
| Class | Very soluble |
| Log *S* (SILICOS-IT) | -3.31 |
| Solubility | 1.35e-01 mg/ml ; 4.88e-04 mol/l |
| Class | Soluble |
| **Pharmacokinetics** | |
| GI absorption | High |
| BBB permeant | Yes |
| P-gp substrate | No |
| CYP1A2 inhibitor | No |
| CYP2C19 inhibitor | No |
| CYP2C9 inhibitor | No |
| CYP2D6 inhibitor | No |
| CYP3A4 inhibitor | No |
| Log *K*_p_ (skin permeation) | -7.31 cm/s |
| **Druglikeness** | |
| Lipinski | Yes; 0 violation |
| Ghose | Yes |
| Veber | Yes |
| Egan | Yes |
| Muegge | Yes |
| Bioavailability Score | 0.55 |
| **Medicinal Chemistry** | |
| PAINS | 0 alert |
| Brenk | 0 alert |
| Leadlikeness | Yes |
| Synthetic accessibility | 3.73 |

**Table S2. Cell lines used in the study**

| **Cancer type** | **Cell lines** | **FLT3** | **TP53** | **Rearrangements** | **FAB classification** | **Age/Gender** | **Reference** |
| --- | --- | --- | --- | --- | --- | --- | --- |
| **Acute myeloid leukemia**  **(AML)** | MV4-11 | ITD | wt | t(4;11)(q21;q23)  MLL-AF4 | M5 | 10y/Male | (6) |
|  | MOLM-14 | ITD | wt | MLL-AF9  KMT2A::MLLT3 fusion | M5a | 20y/Male | (7) |
|  | U937 | wt | Point mutation, abnormal splicing | t(10;11)(p13;q14)  CALM-AF10  PICALM-MLLT10 fusion | M5 | 37y/Male | (8) |
|  | THP-1 | wt | 26-base deletion | t(9;11)(p21;q23)  MLL-AF9 | M5 | 1y/Male | (9) |
|  | HL60 | wt | Deleted | PML-RARα | M2 | 35y/Female | (10) |
| **Chronic myeloid leukemia**  **(CML)** | K562 | wt | Frameshift mutation | t(15;17)(q21;q24) | M1 | 53y/Female | (11) |
|  | K562R |  |  | ABL point mutations |  |  | (12) |

**ITD: internal tandem duplication; wt: wild type; MLL: mixed-lineage leukemia; KMT2A: lysine methyltransferase 2A; CALM: Clathrin Assembly Lymphoid Myeloid; PICALM: Phosphatidylinositol-binding clathrin assembly protein; PML-RARα: promyelocytic leukemia-retinoic acid receptor alpha.**

**Table S3. Antibody list**

|  | **Target** | **Host** | **Provider** | **Cat. No.** |
| --- | --- | --- | --- | --- |
| **Primary antibodies** | PARP | Rabbit | Cell Signaling | #9542, RRID:AB_2160739 |
|  | Caspase-8 | Mouse |  | #9746, RRID:AB_2275120 |
|  | Caspase-9 | Rabbit |  | #9502, RRID:AB_2068621 |
|  | Mcl-1 | Rabbit |  | #4572S, RRID:AB_2281980 |
|  | DRP1 | Rabbit |  | #5391, RRID:AB_11178938 |
|  | MFN2 | Rabbit |  | #9482, RRID:AB_2716838 |
|  | Bcl-2 | Rabbit |  | #3498, RRID:AB_1903907 |
|  | Bid | Rabbit |  | #2002S, RRID:AB_10692485 |
|  | γH2AX | Rabbit |  | #9718S, RRID:AB_2118009 |
|  | Cleaved caspase-3 | Rabbit |  | #9661, RRID:AB_2341188 |
|  | Caspase-3 | Mouse | Santa Cruz Biotechnology | sc-56053, RRID:AB_781826 |
|  | FIS1 | Mouse |  | sc376447, RRID:AB_11149382 |
|  | MFN1 | Mouse |  | sc-166644, RRID:AB_2142616 |
|  | Bcl-xL | Mouse |  | sc-8392, RRID:AB_626739 |
|  | Cytochrome c | Mouse |  | sc-13156, RRID:AB_627385 |
|  | OPA1 | Mouse | BD Pharmingen | 612606, RRID:AB_399888 |
|  | β-actin | Mouse | Sigma Aldrich | 5441, RRID:AB_476744 |
|  | Ki-67 | Rabbit | Abcam | Ab16667 |
| **Secondary antibodies** | Anti-rabbit |  | Santa Cruz Biotechnology | sc-2357, RRID:AB_628497 |
|  | Anti-mouse |  | GeneTex | GTX213111-01,RRID:AB_10618076 |
|  | Anti-rabbit Alexa 488 |  | Invitrogen | A11034, RRID:AB_2576217 |

**
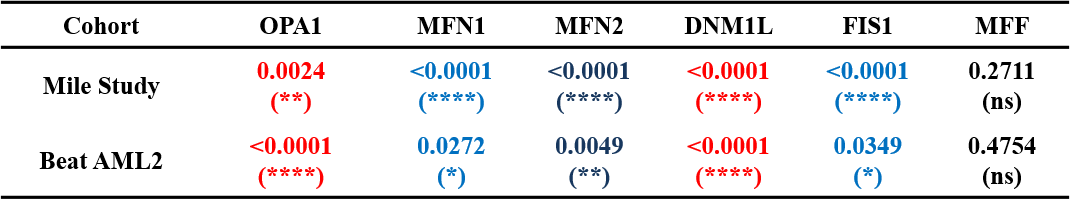
**

**Table S4. Mitofactor modulation in AML vs. Healthy BM cells.**

Red: significantly upregulated genes; blue: significantly downregulated genes; black: not significantly modulated genes.

**
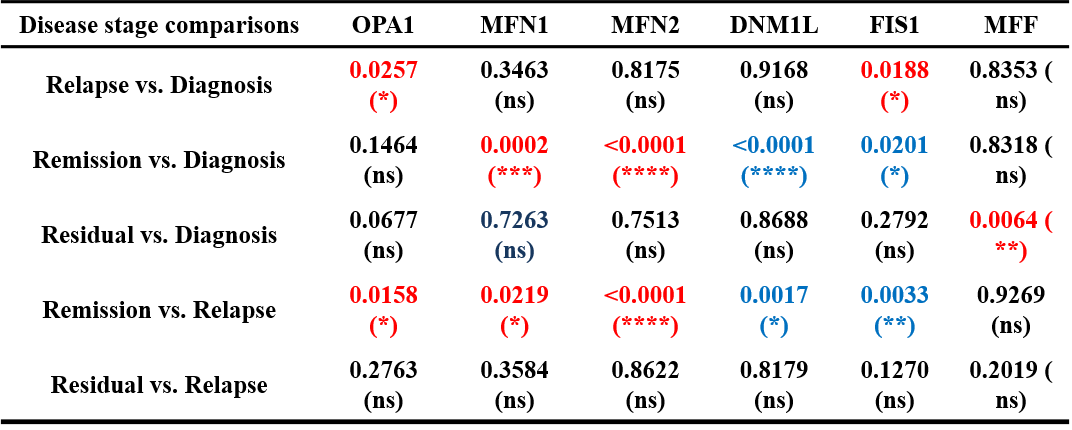
**

**Table S5. Disease stage comparisons of mitofactor modulation.**

Red: significantly upregulated genes; blue: significantly downregulated genes; black: not significantly modulated genes.

**Table S6. Mitofactor modulation significance at different disease stages vs. the overall median.**

**
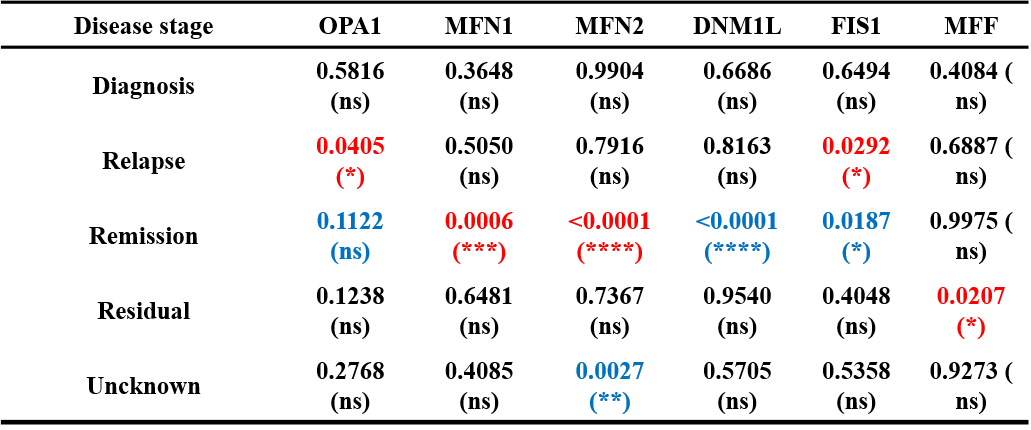
**

Red: significantly upregulated genes; blue: significantly downregulated genes; black: not significantly modulated genes.

**Table S7. Overall survival (months) of relapsing TARGET AML patients.**

**Supplementary Table S6**

**Table S8. The binding affinities between OPA1 and potential TMQ candidates, as well as their respective IC50 values**

| **Candidates** | | **Free Energy of Binding** | **IC_50_ (μM)** | |
| --- | --- | --- | --- | --- |
|  |  |  | **48 h** | **72 h** |
| **** | **TMQ0153** | **-7.506 kcal/mol** | **18.58±3.16** | **14.49±1.27** |
| **** | **TMQ0523** | **-6.276 kcal/mol** | **>50 (ND)** | **38.77±1.47** |
| **** | **TMQ057** | **-5.921 kcal/mol** | **>50 (ND)** | **>50 (ND)** |

ND: not determined.

**Table S9. Synergistic effect analysis by Compusyn software.**

| **TMQ0153 (μM)** | **Gilteritinib (nM)** | **Effect (Fa)** | **CI** |
| --- | --- | --- | --- |
| **1.0** | **1.0** | **0.17** | **1.30** |
| **1.0** | **5.0** | **0.29** | **0.49** |
| **5.0** | **1.0** | **0.33** | **0.45** |
| **5.0** | **5.0** | **0.53** | **0.13** |

**Table S10. Drug doses, administration method, and treatment schedule.**

| **Drugs** | **Dose** | **Administration method (13)** | **Schedule (13)** | **References** |  |
| --- | --- | --- | --- | --- | --- |
| TMQ0153 | 35 mg/kg | Intraperitoneal (i.p.) injection | every 72 h | (Supplementary Figure 20) | |
| Gilteritinib | 3 mg/kg | Oral administration | daily | (14) | |
| Venetoclax | 70 mg/kg | Oral administration | 5 days weekly | (15) | |
| Azacitidine | 1.5 mg/kg | Subcutaneous injection | 5 days weekly | (16) | |

**Table S11. CompuSyn analysis of 24-h treatment with TMQ0153, venetoclax, and azacitidine using U937-luc cells**

| **TMQ0153**  **(µM)** | **Venetoclax**  **(µM)** | **Azacitidine**  **(μM)** | **Effect (Fa)** | **CI** |
| --- | --- | --- | --- | --- |
| **10** | **1** | **0.5** | **0.59** | **0.15** |
| **10** | **1** | **1** | **0.68** | **0.09** |
| **10** | **3** | **0.5** | **0.73** | **0.06** |
| **10** | **3** | **1** | **0.62** | **0.12** |

**Supplementary References**

1. Waterhouse NJ, Trapani JA. A new quantitative assay for cytochrome c release in apoptotic cells. Cell Death Differ. 2003;10(7):853-5.

2. Jendele L, Krivak R, Skoda P, Novotny M, Hoksza D. PrankWeb: a web server for ligand binding site prediction and visualization. Nucleic Acids Res. 2019;47(W1):W345-W9.

3. Krivak R, Hoksza D. P2Rank: machine learning based tool for rapid and accurate prediction of ligand binding sites from protein structure. J Cheminform. 2018;10(1):39.

4. Abramson J, Adler J, Dunger J, Evans R, Green T, Pritzel A, et al. Accurate structure prediction of biomolecular interactions with AlphaFold 3. Nature. 2024;630(8016):493-500.

5. Mikhailovskii O, Izmailov SA, Xue Y, Case DA, Skrynnikov NR. X-ray Crystallography Module in MD Simulation Program Amber 2023. Refining the Models of Protein Crystals. J Chem Inf Model. 2024;64(1):18-25.

6. Lange B, Valtieri M, Santoli D, Caracciolo D, Mavilio F, Gemperlein I, et al. Growth factor requirements of childhood acute leukemia: establishment of GM-CSF-dependent cell lines. Blood. 1987;70(1):192-9.

7. Matsuo Y, MacLeod RA, Uphoff CC, Drexler HG, Nishizaki C, Katayama Y, et al. Two acute monocytic leukemia (AML-M5a) cell lines (MOLM-13 and MOLM-14) with interclonal phenotypic heterogeneity showing MLL-AF9 fusion resulting from an occult chromosome insertion, ins(11;9)(q23;p22p23). Leukemia. 1997;11(9):1469-77.

8. Sundstrom C, Nilsson K. Establishment and characterization of a human histiocytic lymphoma cell line (U-937). Int J Cancer. 1976;17(5):565-77.

9. Tsuchiya S, Yamabe M, Yamaguchi Y, Kobayashi Y, Konno T, Tada K. Establishment and characterization of a human acute monocytic leukemia cell line (THP-1). Int J Cancer. 1980;26(2):171-6.

10. Collins SJ, Gallo RC, Gallagher RE. Continuous growth and differentiation of human myeloid leukaemic cells in suspension culture. Nature. 1977;270(5635):347-9.

11. Lozzio CB, Lozzio BB. Human chronic myelogenous leukemia cell-line with positive Philadelphia chromosome. Blood. 1975;45(3):321-34.

12. Kim TM, Ha SA, Kim HK, Yoo J, Kim S, Yim SH, et al. Gene expression signatures associated with the in vitro resistance to two tyrosine kinase inhibitors, nilotinib and imatinib. Blood Cancer J. 2011;1(8):e32.

13. Diehl KH, Hull R, Morton D, Pfister R, Rabemampianina Y, Smith D, et al. A good practice guide to the administration of substances and removal of blood, including routes and volumes. J Appl Toxicol. 2001;21(1):15-23.

14. Marjoncu D, Andrick B. Gilteritinib: A Novel FLT3 Inhibitor for Relapsed/Refractory Acute Myeloid Leukemia. J Adv Pract Oncol. 2020;11(1):104-8.

15. Li D, Zhao S, Mao L, Jin J, Wang J. Salvage treatment in IDH1 mutated acute lymphoblastic leukemia with venetoclax plus methotrexate and pegaspargase: A case report. Genes Dis. 2023;10(6):2215-7.

16. El Khawanky N, Hughes A, Yu W, Myburgh R, Matschulla T, Taromi S, et al. Demethylating therapy increases anti-CD123 CAR T cell cytotoxicity against acute myeloid leukemia. Nat Commun. 2021;12(1):6436.
